# Using metabolomics to predict severe traumatic brain injury outcome (GOSE) at 3 and 12 months

**DOI:** 10.1186/s13054-023-04573-9

**Published:** 2023-07-22

**Authors:** Mohammad M. Banoei, Chel Hee Lee, James Hutchison, William Panenka, Cheryl Wellington, David S. Wishart, Brent W. Winston, the Canadian biobank, database for Traumatic Brain Injury (CanTBI) investigators, the Canadian Critical Care Translational Biology Group (CCCTBG), the Canadian Traumatic Brain Injury Research, Clinical Network (CTRC), Ari Joffe, Karen Barlow, Keith Yeates, Michael Esser, Brent Winston, Cheryl Wellington, Ivan Torres, Keith Walley, Noah Silverberg, Priscilla Carrion, Quynh Doan, Sophie Stukas, Susan Vercauteren, Will Panenka, Angela Aquino, Audas Lorelei, David Clarke, Kelly Martin, Adrienne Davis, Angela Colantonio, Anne Wheeler, Anne-Marie Guerguerian, Andrew Baker, Elaine Gilfoyle, Jamie Hutchison, Lili-Naz Hazrati, Robin Green, Shannon Scratch, Elisa Wilson, Arash Khosroawshahi, Catherine Farrell, Jacques Lacroix

**Affiliations:** 1 Department of Critical Care Medicine, University of Calgary, Alberta, Canada; 2 Department of Pediatrics and Critical Care and Neuroscience and Mental Health Research Program, SickKids and Interdepartmental Division of Critical Care and Institute for Medical Science, The University of Toronto, Toronto, ON Canada; 3 BC Mental Health and Substance Use Research Institute and the Department of Psychiatry, Faculty of Medicine, University of British Colombia, British Colombia, Canada; 4 Djavad Mowafaghian Centre for Brain Health, University of British Columbia, British Colombia, Canada; 5 Department of Biological Sciences, Computing Sciences and Medicine and Dentistry, University of Alberta, Alberta, Canada; 6 Department of Critical Care Medicine, Medicine and Biochemistry and Molecular Biology, University of Calgary, Health Research Innovation Center (HRIC), Room 4C64, 3280 Hospital Drive N.W., Calgary, AB T2N 4Z6 Canada; 7 Stollery Children’s Hospital, The University of Alberta, Alberta, Canada; 8 University of Queensland, Brisbane, Australia; 9 Alberta Children’s Hospital, University of Calgary, Alberta, Canada; 10 Foothill Medical Centre, The University of Calgary, Calgary, AB Canada; 11 University of British Columbia, Vancouver, BC Canada; 12 BC Children’s Hospital, The University of British Colombia, Vancouver, BC Canada; 13 St Paul’s Hospital, The University of British Colombia, Vancouver, BC Canada; 14 Queen Elizabeth II Health Sciences Centre, Halifax, NS Canada; 15 Hospital for Sick Children, Toronto, ON Canada; 16 Toronto Rehabilitation Institute, Toronto, ON Canada; 17 St Michael’s Hospital, Toronto, ON Canada; 18 Holland Bloorview, Toronto, ON Canada; 19 CHU Ste Justine, Montreal, QC Canada

**Keywords:** Severe TBI, Metabolomics, Outcome prediction, Prediction modelling

## Abstract

**Background:**

Prognostication is very important to clinicians and families during the early management of severe traumatic brain injury (sTBI), however, there are no gold standard biomarkers to determine prognosis in sTBI. As has been demonstrated in several diseases, early measurement of serum metabolomic profiles can be used as sensitive and specific biomarkers to predict outcomes.

**Methods:**

We prospectively enrolled 59 adults with sTBI (Glasgow coma scale, GCS ≤ 8) in a multicenter Canadian TBI (CanTBI) study. Serum samples were drawn for metabolomic profiling on the 1st and 4th days following injury. The Glasgow outcome scale extended (GOSE) was collected at 3- and 12-months post-injury. Targeted direct infusion liquid chromatography-tandem mass spectrometry (DI/LC–MS/MS) and untargeted proton nuclear magnetic resonance spectroscopy (^1^H-NMR) were used to profile serum metabolites. Multivariate analysis was used to determine the association between serum metabolomics and GOSE, dichotomized into favorable (GOSE 5–8) and unfavorable (GOSE 1–4), outcomes.

**Results:**

Serum metabolic profiles on days 1 and 4 post-injury were highly predictive (Q^2^ > 0.4–0.5) and highly accurate (AUC > 0.99) to predict GOSE outcome at 3- and 12-months post-injury and mortality at 3 months. The metabolic profiles on day 4 were more predictive (Q^2^ > 0.55) than those measured on day 1 post-injury. Unfavorable outcomes were associated with considerable metabolite changes from day 1 to day 4 compared to favorable outcomes. Increased lysophosphatidylcholines, acylcarnitines, energy-related metabolites (glucose, lactate), aromatic amino acids, and glutamate were associated with poor outcomes and mortality.

**Discussion:**

Metabolomic profiles were strongly associated with the prognosis of GOSE outcome at 3 and 12 months and mortality following sTBI in adults. The metabolic phenotypes on day 4 post-injury were more predictive and significant for predicting the sTBI outcome compared to the day 1 sample. This may reflect the larger contribution of secondary brain injury (day 4) to sTBI outcome. Patients with unfavorable outcomes demonstrated more metabolite changes from day 1 to day 4 post-injury. These findings highlighted increased concentration of neurobiomarkers such as N-acetylaspartate (NAA) and tyrosine, decreased concentrations of ketone bodies, and decreased urea cycle metabolites on day 4 presenting potential metabolites to predict the outcome. The current findings strongly support the use of serum metabolomics, that are shown to be better than clinical data, in determining prognosis in adults with sTBI in the early days post-injury. Our findings, however, require validation in a larger cohort of adults with sTBI to be used for clinical practice.

**Supplementary Information:**

The online version contains supplementary material available at 10.1186/s13054-023-04573-9.

## Introduction

Traumatic brain injury (TBI) is a neurologic injury resulting from an external mechanical force and is one of the most common causes of long-term neurological disability and death [1]. Worldwide, approximately 69 million people suffer TBI annually [2]. There are 5.3 and 7.7 million individuals living with TBI-related disability in the USA and European countries [1], respectively. Severe TBI has a mortality of 30–50% and 30% of survivors have severe neurologic sequelae [3–7]. The prognosis of TBI outcomes in the first days post-injury is difficult because of the high variability in the mechanisms of TBI, types of brain injury and multiple outcomes [8]. Assessing the severity and prognosis of brain injury, especially in the context of severe traumatic brain injury (sTBI), is an important concern in the field of medicine. Early prediction of outcomes is critical in guiding treatment decisions, providing appropriate care, and providing support to patients and their families. Clinical factors, neuroimaging findings and electrophysiological events (e.g., EEG) are associated with a large degree of uncertainty to predict TBI outcomes [9, 10]. Blood biomarkers (proteins and metabolites) have gained significant interest in sTBI investigations in recent years. They have the potential to improve diagnosis, help with prognosis, and aid in the management of sTBI with or without physiological parameters.

Prognostic models play an important role in helping clinicians make treatment decisions for patients with sTBI. These models can provide valuable information for predicting outcomes, which can help with critical care needs, rehabilitation plans, and support services for survivors and their caregivers.

Several studies have investigated the role of protein [11] and metabolite biomarkers in the prognosis of sTBI to predict the likelihood of an unfavorable and favorable outcome. The most studied protein biomarkers in all types of TBI include neurofilament light/or heavy (NFL/NFH), S100B, glial fibrillary acidic protein (GFAP), tau protein, neuron-specific enolase (NSE) and myelin basic protein (MBP) [11]. The serum and CSF levels of these biomarkers were used to predict the TBI outcome based on the Glasgow Outcome Scale (GOS) or Glasgow Outcome Scale Extended (GOSE) at different time points (3-, 6- and 12-months post-injury) [11]. Serial sampling has offered valuable insights to understand the kinetic profile of protein biomarkers and evaluate their trajectories that could help to increase biomarkers’ clinical prognostic value [11]. Metabolomics studies have shown potential insights into mechanisms of injury and may allow the development of sensitive and specific biomarkers for prognostic models [12]. However, few studies have investigated the potential use of metabolites in the prognosis of TBI, or in predicting the severity and stratification of all types of TBI. Of note, phospholipids, energy-related metabolites (lactate, glucose, and TCA cycle compounds), NAA and aromatic amino acids have been shown to correlate with TBI outcomes.[13–20].

In this study, we hypothesized that serum metabolites could predict the severe TBI outcome at 3- and 12 months post-injury. We aimed to determine the prognostic threshold and compare prognostic models using metabolomic biomarkers with those using clinical predictors. Therefore, we measured the concentration levels of metabolites in serum samples collected at 1 and 4 days after severe TBI.

## Materials and methods

### Patients’ characteristics and primary clinical information

Patients ≥ 18 years old with severe TBI (Glasgow coma scale ≤ 8) were enrolled prospectively at 3 hospitals in Vancouver, Calgary and Halifax, Canada. Serum samples were collected on days 1 (within the first 24 h after injury) and 4 post-injury using specific standard operating procedures (SOPs). Demographics, injury characteristics, neuroimaging (CT scan) and physiologic clinical variables were collected electronically as well as global neurological function at 3- and 12-months following injury using the Glasgow Outcome Scale-Extended (GOSE) and mortality at 3 months. All data were collected and cleaned by trained research coordinators and database engineers. The GOSE was dichotomized into favorable (GOSE 5–8) and unfavorable (GOSE 1–4) outcomes as a commonly used approach [21–24]. In this study, we also used the ordinal GOSE values in the prediction models, but this approach was less predictive and was more difficult to interpret than using a dichotomized GOSE approach, likely because of the relatively small sample size. The collected clinical variables included gender, age, GCS, ISS (injury severity score), intubation, hypoxemia, hypotension, loss of consciousness and Marshall CT scan classification that were used for the prediction of GOSE outcome at 3 and 12 months and mortality at 3 months.

### Metabolomics methods and quantification

Untargeted proton nuclear magnetic resonance (^1^H-NMR) spectroscopy and targeted direct injection, liquid chromatography-tandem mass spectrometry (DI/LC–MS/MS) were applied to identify and quantify serum metabolites on days 1 and 4 post sTBI. These two techniques were used to quantify a broad list of metabolites with few overlapping metabolites. We carried out a comprehensive targeted analysis of 130 and 58 metabolites using DI/LC–MS/MS and ^1^H-NMR, respectively, to measure serum metabolite concentrations on days 1 and 4 post-injury. More details about these methods are available in the Additional file 1.

### Statistical analysis

Multivariate analysis and machine learning methods were used to build prediction models using serum metabolites and clinical parameters for the prognosis of GOSE-based favorable versus unfavorable outcomes. For multivariate analysis (PCA and PLS-DA) and machine learning analysis (SIMPLS), we normalized (median fold normalization) and transformed (log transformation) the metabolomics data because they were not normally distributed and since different metabolites with large differences in concentration were used together [25]. We also used non-normalized and non-transformed data (raw data) for univariate analysis such as the Student’s t-test. The Shapiro–Wilk test confirmed that the majority of the raw data did not follow a normal distribution. Thus, the data was normalized and transformed for analysis. Principal component analysis (PCA) was applied as a multi-variable analysis method to examine the variability and trends of metabolic profiles and to detect outliers. Partial least squares discriminant analysis (PLS-DA), a type of machine learning, was used to build prognostic models. PLS-DA-based prognostic models were created based on the most differentiating metabolites with a variable important of the projection (VIP) value >|1.0| using SIMCA-P v15.0.2 (Sartorius Stedim Biotech, Umea, Sweden). We further analyzed whether clinical predictors or combining clinical predictors with metabolomics data can enhance the prognosis of outcomes. Statistically inspired modification of partial least squares (SIMPLS), an algorithm of the PLS method suitable for both nominal or continuous variables, was used to develop prediction models using only clinical predictors or combined clinical with metabolite variables for outcomes at 3 months, 12 months, and mortality at 3 months. Developed prognostication models were characterized by the metrics R^2^ (goodness of model fit), Q^2^ (goodness of prediction showing the predictability of the statistical model) [26], cross-validation p-value was determined and permutation testing (200 times) was used to check the R^2^ and Q^2^ values to prevent overfitting. Artificial neural network analysis (ANN), a machine learning method, was performed to predict one response variable (unfavorable and/or favorable separately) using a flexible function of input variables. JMP Pro 14.3.0 (SAS Institute Inc. USA) was used for SIMPLS and ANN analysis. MetaboAnalyst 4.0 (available at www.metaboanalyst.ca) was used for multivariate and univariate analysis. The area under the receiver operating curve (AUC), sensitivity, and specificity was obtained using a multivariate approach.

To build prognostic models of outcome using clinical factors, we first used univariate analysis followed by multivariable analysis and generated AUC information. Clinical factors with a *p* < 0.05 from the univariate analysis were included in the multi-variable models. More details regarding our methods of data and statistical analysis are available in the Additional file 1. In general, our approach is to remove any extreme outliers identified using PCA analysis. However, in this study, there were no extreme outliers among datasets to remove. Also, we excluded the metabolites that were below the limit of detection and metabolites with a lot of missing values. The terms “increased” and “decreased” values of the metabolites imply the relative changes in concentrations of metabolites between the unfavorable and favorable outcomes.

## Results

### Patient characteristics

**Fig. 1:**
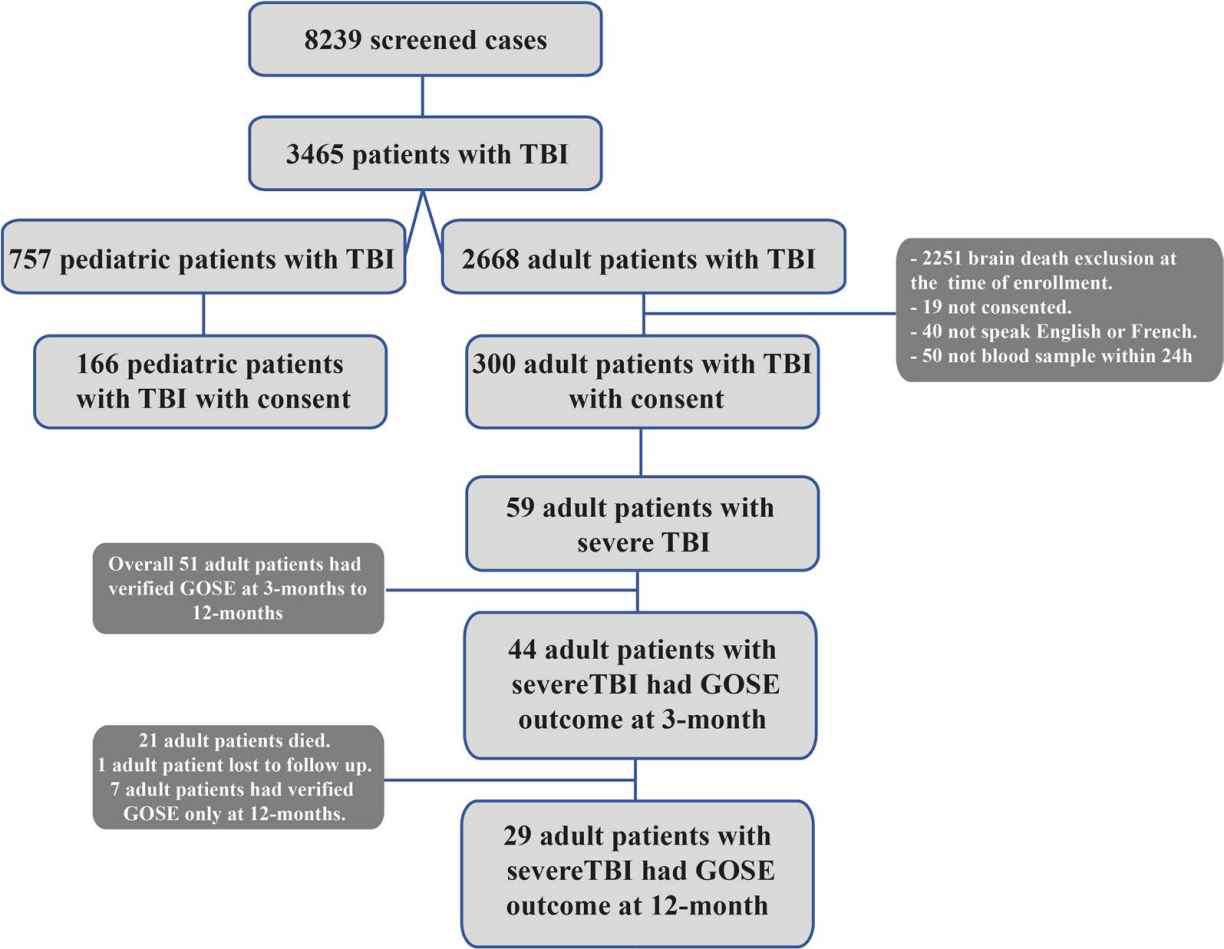
The patient flow chart reflects patients screened, enrolled in the CanTBI tissue bank and database and patients with measured GOSE outcomes at 3 and 12 months

A total of 8239 patients were screened in the CanTBI study (Fig. 1); 3465 patients screened positive for TBI (42%). After informed consent, 466 adult and pediatric patients with mild, moderate, and severe TBI were enrolled into the prospective CanTBI biobank and database for the TBI study. There were 300 adult patients with TBI and 59 of these patients (19.6%) were diagnosed with severe TBI (sTBI) and were included in this study. These 59 patients had a mean age of 50 years ± 20.6 (SD). For detailed patient and injury characteristics see Table 1. Figure 1 shows the patient flow chart with the numbers of patients recruited and follow-up data at 3- and 12-months post-injury (n = 51). Table 2 shows the differences in clinical variables between favorable and unfavorable outcomes at 3- and 12 months post-injury. Age and injury severity score (ISS) were significantly (*p* < 0.05) associated with unfavorable outcomes at 3 months, but not significant at 12 months. There was a significant difference in age and ISS between patients who died (n = 21) and those who survived (n = 23) at 3 months (Additional file 1: Table S1), which suggests that older age and higher ISS are associated with unfavorable outcomes. We then determined the cut points of ISS and age at ≥ 75 and ≥ 49, respectively, to separate non-survivors from survivors at 3 months. Also, the cut points were calculated for Marshall classification = 4 and GCS = 6 between non-survivors and survivors. These two variables were not statistically significantly different between the two cohorts but had a higher impact using multivariate data analysis.

**Table 1:** Patients’ characteristics, clinical information, GCS at admission, GOSE outcome distribution, CT findings, and Marshall CT Classification

Patients characteristics	Subcategory/unit	n = 59 severe TBI
Sex	Male/Female	48/11
Age	Mean (± SD)	50 ± 20.6
Weight	Mean (± SD)	82 ± 19.0
Admission type ER ICU	n (%)	19 (32.3) 40 (67.7)
Severity (ISS)	Mean (± SD)	43.3 ± 19
Intubated	Yes (%)*	40 (67.7)
Hypoxia	Yes (%)*	8 (13.5)
Hypotension	Yes (%)*	9 (15.2)
Paralytic agent	Yes (%)*	30 (50.8)
Loss of consciousness	Yes (%)*	40 (67.7)
GCS (total) GCS-Motor GCS-Eye GCS-Verbal	Mean (± SD)	5.46 ± 2.27 2.87 ± 2.07 1.54 ± 1.02 0.98 ± 0.71
GCS (categorized) GCS 3–4 GCS 5–6 GCS 7–8	n (%)**	26 (44) 6 (6.7) 26 (44)
GOSE 3-month Poor Good 6-month Poor Good 12-month Poor Good	n (%)^ŧ^	44 (74.5) 35 (59.3) 9 (15.2) 22 (37.2) 9 (15.2) 13 (22) 29 (49.7) 14 (23.7) 15 (25.4)
GOSE 1 & 2 (3 month)	n (%)	21 (35.5)
CT findings Diffuse Axonal Injury Mild Shift Skull Fracture Cerebral edema Contusion Intracranial hemorrhage Epidural hemorrhage Subdural hemorrhage Arachnoid hemorrhage	(Yes/No) ^ŧ ŧ^	35/7 14/26 28/14 10/32 18/24 26/16 5/37 30/12 32/10
Marshall score I II III IV V	n (%)	1(2.3) 23 (54.7) 6 (14.2) 5 (11.9) 7 (16.6)

*Shows the number of patients with the clinical information and the percentage of total patients, others included without clinical information and missing information. ** The number of patients (percentage of total) were included in the same GCS categorized level. ŧ the number of patients with GOSE data at the same time. ŧ ŧ the number of patients that had the same CT findings; the rest may include patients without CT findings or findings missing in the study

^*^Shows the number of patients that had intubation and mentioned physiological conditions. ** shows the number of patients in each categorized GCS group among all patients(n = 59). ^ŧ^ shows the number patients for unfavorable and favorable outcome at different time, ^ŧ ŧ^ shows the number of patients that had each brain damage captured on CT

**Table 2:** Patient demographics and clinical characteristics for unfavorable (GOSE 1–4) and favorable (GOSE 5–8) outcome groups at 3 and 12 months

Prediction of GOSE	3 Month			12 Month		
Patients characteristics and clinical information	unfavorable outcome (n = 35)	Favorable outcome (n = 9)	p value	Unfavorable outcome (n = 14)	Favorable outcome (n = 15)	p value
Sex (male/female)	30/5	6/3	0.42	11/3	13/2	0.82
Age (mean ± SD)	55.4 ± 20.4	40.5 ± 21.0	0.03	52.0 ± 18.7	38 ± 19.8	0.06
Weight (mean ± SD)	88.5 ± 19.5	76.4 ± 21.1	0.08	81.7 ± 22.6	79.3 ± 16.1	0.75
Injury severity score (ISS) (mean ± SD)	56.4 ± 22.6	35.1 ± 12.6	< 0.01	35.5 ± 12.5	36.4 ± 12.5	0.81
Admission-type ER ICU	13 (37.1%) 21 (60%)	2 (22.2%) 7 (77.7%)	0.36	4 (28.5%) 10 (71.4%)	4 (26.6%) 11 (73.3%)	0.58
Hypoxia (yes/no) *	8/22	0/9	0.07	3/8	1/14	0.38
Intubated (yes/no) *	21/13	7/2	0.61	11/3	10/5	0.77
Hypotension (yes/no) *	5/25	1/7	0.98	2/10	2/13	0.64
Paralytic-AGT (yes/No) *	16/17	6/1	0.40	6/7	9/4	0.32
Loss consciousness*	25/4	5/2	0.30	13/0	8/2	0.48
Location of injury ^ŧ^			0.70			0.52
Type of injury ^ŧ^			0.24			0.21
GCS (total) (mean ± SD)	5.3 ± 2.17	5.3 ± 2.5	0.95	4.5 ± 1.9	5.8 ± 2.3	0.11
GCS-motor GCS-eye (mean ± SD) GCS-verbal	2.9 ± 1.9 1.5 ± 1.1 1.0 ± 0.75	2.4 ± 2.2 1.0 ± 0.0 1.1 ± 0.78	0.54 0.14 0.94	2.28 ± 2.0 1.4 ± 0.99 0.71 ± 0.48	2.7 ± 2.1 1.6 ± 1.3 1.13 ± 0.74	0.57 0.68 0.08
GCS 3–4 GCS 5–6 (mean ± SD) GCS 7–8	15 (42.5%) 6 (14.1%) 14 (40%)	5 (55.5%) 0 4 (44.4%)	0.80	9 (64.2%) 1 (7.1%) 4 (28.5%)	6 (40%) 1 (6.6%) 8 (53.3%)	0.62
CT findings** Diffuse axonal injury Mid shift Skull fracture Cerebral edema Contusion Intracranial hemorrhage Epidural hemorrhage Subdural hemorrhage Arachnoid hemorrhage Marshall score I II III IV V	5/20 6/18 209/6 6/20 14/12 18/8 3/23 20/6 22/4 0 17 4 3 2	0/7 4/3 3/4 0/7 2/5 3/4 0/7 5/2 5/2 0 3 1 0 3	0.57 0.41 0.30 0.24 0.33 0.53 0.62 0.34 0.37 0.19	2/7 4/4 7/2 3/6 3/6 5/4 7/2 8/2 6/3 0 4 1 3 1	2/11 6/7 9/4 2/11 7/6 8/5 9/4 8/4 8/5 0 6 2 1 4	0.62 0.16 0.52 0.49 0.47 0.63 0.11 0.86 0.16 0.37

*The variables are based on the number of patients. ^ŧ^ These data included several variables that have not been shown in detail for each cohort. There was no significant difference for any type and location of injury between cohorts with favorable and unfavorable outcomes at 3- and 12 months post-injury

^*^Shows the number of patients that had intubation and mentioned physiological conditions in each unfavorable and favorable group. ^ŧ^ the details have not been shown due to several conditions and table limitation. ** shows the number of patients that had brain damage captured on CT for unfavorable and favorable group

### Identified, quantified metabolites

130 and 58 metabolites from different metabolite classes were identified and quantified using targeted DI/LC–MS/MS and untargeted ^1^H-NMR, respectively (Table S2-S3). Twenty-four of the 30 common metabolites measured by each technique had a similar trend of change, showing the accuracy of both techniques. See Additional file 1 for more details.

#### Metabolomics for the prognosis of 3- and 12-month outcomes of sTBI

Prediction models show that a serum metabolic biosignature can be used to prognosticate GOSE outcome at 3 and 12 months and the mortality outcome at 3 months.

**Fig. 2:**
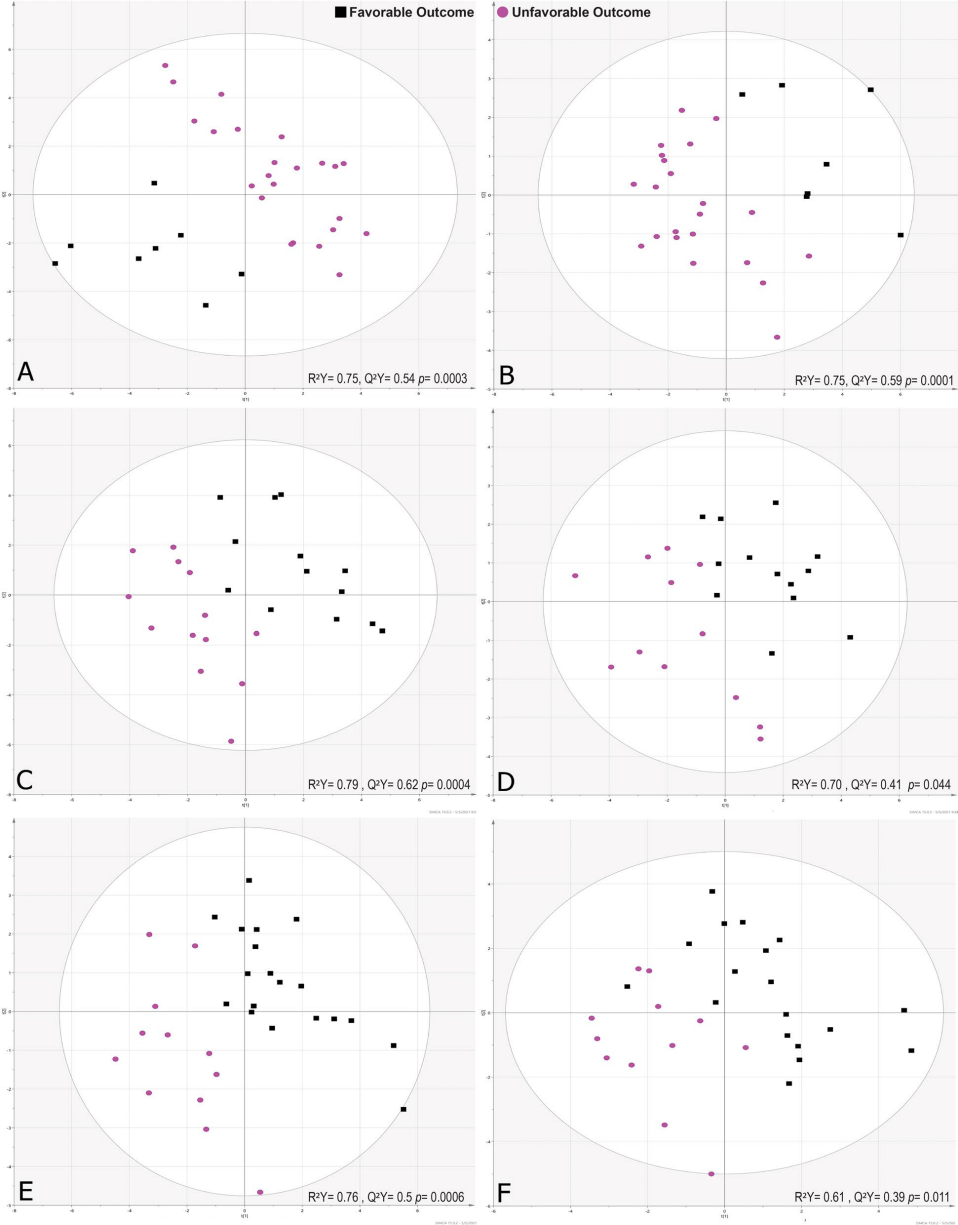
PLS-DA scatter plots: discrimination models show highly predictive (Q^2^) separation of patients with unfavorable outcome (purple filled circle) from the favorable outcome (black filled square) based on serum metabolomic profiling on day 4 and GOSE at 3 months. **A** DI/LC–MS/MS using 24 metabolites, **B** ^1^H-NMR using 9 metabolites. The high predictability is visualized by a good separation between the two cohorts and yielding a Q^2^ > 0.5. The model metrics for the day 4 DI/LC–MS/MS model and GOSE outcome at 3 months are R^2^Y = 0.75, Q^2^Y = 0.54 and p = 0.0003 and for day 4 ^1^H-NMR model and GOSE outcome at 3 months are R^2^Y = 0.75, Q2Y = 0.59 and p = 0.0001. Metabolomic profiling on day 4 for GOSE at 12 months. **C** DI/LC–MS/MS using 29 metabolites, **D** ^1^H-NMR using 9 metabolites. The metabolic profile on day 4 serum samples analyzed using DI/LC–MS/MS was more predictive (Q^2^ = 0.62) than ^1^H-NMR (Q^2^ = 0.41). GOSE outcome at 3 months is R^2^Y = 0.75, Q2Y = 0.59 and p = 0.0001. Metabolomic Mortality outcome at 3 months: non-survivor (purple filled circle) versus survivor outcome (black filled square). **E** DI/LC–MS/MS using 16 metabolites, Q^2^ = 0.50. **F** ^1^H-NMR using 16 metabolites. Q^2^ = 0.39. These Q^2^ values show a high predictability of metabolic profile on day 4 with DI/LC–MS/MS being better than ^1^H-NMR to predict mortality at 12 months and mortality outcome

Unsupervised PCA showed a relatively good grouping between cohorts with unfavorable and favorable outcomes using all metabolites detected in serum samples collected on days 1 and 4. PCA revealed a high level of variability (R^2^X > 0.5) of metabolites suggesting a differential biosignature between the two cohorts (Additional file 1: Fig S1–S3). Metabolic biosignatures obtained by DI/LC–MS/MS using samples on day 4 presented clearer groupings between unfavorable and favorable cohorts compared with ^1^H-NMR and samples on day 1. The PLS-DA-based analysis demonstrated a good predictive (Q^2^ > 0.5), highly significant (*p* < 0.001) and highly sensitive and specific (> 99%) prediction model to discriminate between patients with unfavorable and favorable outcomes using a serum metabolic biosignature on day 4 obtained by DI/LC–MS/MS (Table 3 and Fig. 2). Nonetheless, day 1 metabolic biosignatures were also significant predictors for GOSE outcomes (Additional file 1: Fig. S4). The permutation analysis (200 times permuted, not shown) verified that the models are valid and unlikely to be overfit. ANN indicated the higher predictability (AUC > 0.90) for the prognosis of GOSE outcome among patients with unfavorable outcomes compared to favorable outcomes at 3 months (Tables S4-S5). Also, ANN showed higher predictability (AUC > 0.90) for the prognosis of GOSE outcome among patients with favorable outcomes compared to unfavorable outcomes at 12-months. This was based primarily on DI/LC–MS/MS data on day 4. See Additional file 1 for more details.

**Table 3:** Prediction models’ characteristics show a higher predictability of metabolic profiles on day 4 than day 1 post-sTBI for 3- and 12-month GOSE and mortality at the 3-month outcome

Prognosis	Analytical platforms	Sampling time	R^2^	Q^2^	*p* value*	Sensitivity	Specificity	AUC	# Metabolites
Unfavorable versus favorable outcome 3-month	DI-MS/MS	Day 1	0.60	0.40	0.0004	93	100	0.99	26
		Day 4	0.75	0.54	0.0003	100	100	1.00	24
	^1^H-NMR	Day 1	0.47	0.25	0.017	72	100	0.92	10
		Day 4	0.75	0.59	0.0001	100	96	1.00	9
Unfavorable versus favorable outcome 12-month	DI-MS/MS	Day 1	0.88	0.58	0.0002	100	100	0.99	21
		Day 4	0.79	0.62	0.0004	100	100	0.98	29
	^1^H-NMR	Day 1	0.64	0.46	0.003	76	91	0.91	12
		Day 4	0.7	0.41	0.044	100	100	1.00	9
Mortality outcome	DI-MS/MS	Day 1	0.54	0.35	0.002	79	100	0.98	19
		Day 4	0.76	0.50	0.0006	100	100	1.00	16
	^1^H-NMR	Day 1	0.50	0.24	0.01	84	87	0.88	17
		Day 4	0.61	0.39	0.011	91	90	0.96	16

^*^p value < 0.05 was considered significant. Additionally, the metabolic profiles obtained by DI/LC–MS/MS are more predictive than ^1^H-NMR results. R^2^, the goodness of fit of the model; Q^2^, the goodness of prediction of the model: and AUC, the area under the receiver operating curve of the model

Further analyses were performed to investigate the relative correlation of the most differentiating metabolites between unfavorable and favorable outcomes for each prediction model. Different prediction models consisted of 9 to 26 metabolites that differentially contributed to the models (Additional file 1: Figs. S5–S16). A predictive metabolic biosignature to predict GOSE outcome at 3 months was characterized by an increase in lysoPCs, propionic acid (C3:1), stearic acid (C18), oleic acid (C18:1), linoleic acid (C18:2), and myristic acid (C14) on the 1st-day post-injury yielding an unfavorable outcome (Additional file 1: Figs. S5A and B). Also, a decrease in methionine-sulfoxide, glutamate, histidine, citrulline, isoleucine, glutamine, phenylalanine, and asparagine were associated with an unfavorable outcome on the 1st-day post-injury (Additional file 1: Figs. S5A & B). Interestingly, a predictive metabolic biosignature on day 4 (Additional file 1: Figs. S6 and S8) showed increased glutamate (excitotoxicity), propionic acid, linoleic acid, valeric acid (C5), indole acetic acid, ɑ-ketoglutaric acid, ɑ-aminoadipic acid, alanine, lysoPCs (18:2, 18:0 & 17:0), tyrosine, NAA, aspartate, and valine in those with an unfavorable outcome, while these metabolites were decreased on day 1 post-injury. For prognosis of GOSE at 12-months, patients with unfavorable outcome were characterized by increased lysoPCs (14:0, 20:3 & 28:1), short chain ACs (C5OH, C3, C0, C4), ornithine, sphingomyelin (16:1), valine, serine, leucine, lactate, and a decrease in trans-hydroxyproline, serine, serotonin, citrulline, spermine, methionine-sulfoxide, acetylornithine and medium-chain acylcarnitines on day 1 post-injury (Additional file 1: Figs. S9 and S11). In addition, a 12-month unfavorable outcome was associated with increased lysoPCs (28:1, 14:0), tryptophan, caproic acid (C6), oleic acid, tyrosine, creatinine, alanine, histidine, valine, and leucine on day 4 post-injury (Additional file 1: Figs. S10 and S12). To predict 3-month mortality, metabolomic analysis showed increased glucose, PCs (38:0aa, 40:6 ae), acylcarnitines (C3:1, C10:1, C14:1 C14, C10, C16:2 C8), betaine, 3-hydroxy isovalerate, citrate, O-phosphocholine, formate, fumarate, and pyruvate on day 1 in patients predicted to die by 3 months (Additional file 1: Figs S13 and S14). Those patients predicted to die showed decreased glutamine, and branched-chain amino acids, citrulline and histidine on day 1 (Additional file 1: Fig. S13 and S15). Increased ɑ-ketoglutaric acid, hippuric acid, indole acetic acid, ornithine tryptophan, ɑ-aminoadipic acid, PCs (38:0aa, 36:0aa), branched-chain amino acids, creatine, creatinine, tyrosine and threonine were found in those patients predicted to die based on the day 4 metabolic profile (Figs S14 and S16). Univariate T-test analysis showed remarkable similarities to PLS-based prediction models to identify predictive biomarkers (Additional file 1: Figs. S5–S16). See Additional file 1 for more details.

**Fig. 3:**
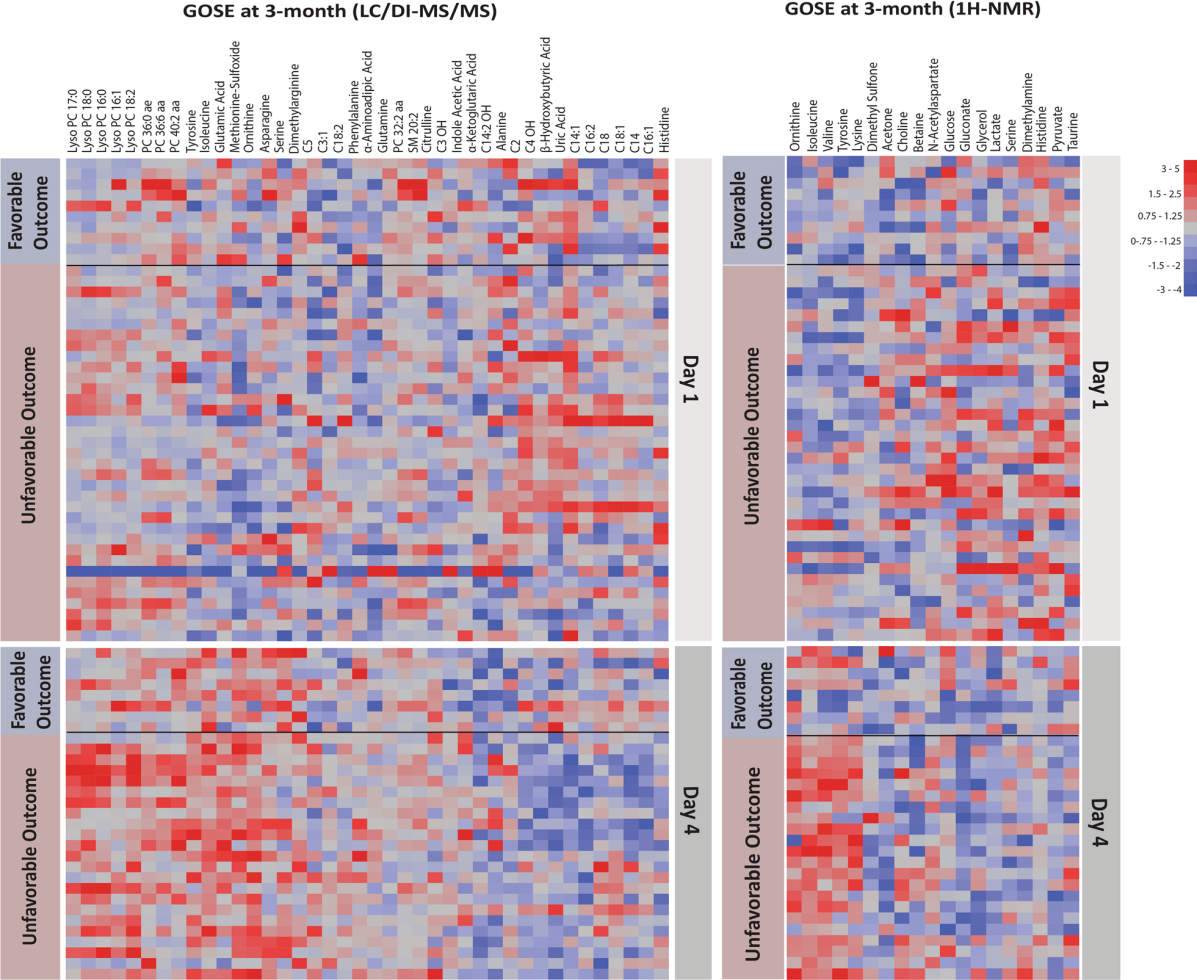
Heatmap metabolite plots show the metabolite alterations at day 1 and day 4 of the most differentiating metabolites to predict unfavorable and favorable outcomes at 3 months using DI/LC–MS/MS and ^1^H-NMR. The figure reveals the changes in metabolites for day 1 or day 4 used to prognosticate GOSE outcome at 3 months with increased changes seen in metabolites from day 1 to day 4 among patients with unfavorable outcomes. The heatmap key shows the normalized and transformed concentration for each metabolite. Since each metabolite has its own relative concentration in the cell plots the key shows the range of relative concentrations

Metabolite heatmap plots (Figs. 3, Additional file 1: Figs. S17–S18) directly visualized the metabolite alterations on the same days and from day 1 to day 4 for both cohorts with unfavorable and favorable outcomes. These results show a higher level of metabolite alterations from day 1 to day 4, particularly for predicting unfavorable outcomes compared to favorable outcomes. Overall, metabolite changes are enhanced among the patients with unfavorable outcomes. Lysophosphatidylcholines (16:0, 16:1, 17:0, 18:0 and 18:2) and lysophosphatidylcholines (14:0, 20:3, 28:1, and 18:0) showed an increase from day 1 to day 4 mostly in patients with unfavorable outcomes. Also, BCAAs, NAA, tyrosine, ornithine, and glutamate increased from day 1 to day 4 predominantly among the patients with unfavorable outcomes. Histidine, alanine, serine, citrulline, pyruvate and lactate decreased from day 1 to day 4 mainly among the patients with unfavorable outcomes.

A brief overview highlights that unfavorable outcome was associated with increased metabolites related to lipids and “anaerobic” metabolism and decreased metabolites related to serotonergic, polyamine metabolism and NMDA receptor integrity on day 1 post-injury. Increased metabolites related to neuroinflammation, excitotoxicity and brain injury-specific biomarkers were found on day 4 post-injury. Also, notable was an association of increased metabolites related to acylcarnitine metabolism and energy metabolism with mortality.

#### Clinical variables for the prognosis of GOSE outcome at 3 months, 12 months, and mortality

We investigated whether clinical variables could predict the outcome of sTBI at 3- and 12 months post-sTBI. SIMPLS analysis revealed the most differentiating clinical variables for predicting outcomes at 3 months (age, ISS, Marshall classification and hypoxemia) and 12 months (age, GCS, hypoxemia, and loss of consciousness). However, these clinical variables had low prediction capacity (Q^2^ < 0.16) and less sensitivity (66%), and specificity (86%) compared to metabolomics-based prediction (Table S6). SIMPLS analysis of clinical data revealed that age and ISS are useful predictors (Q^2^ = 0.37, AUC = 0.86) to prognosticate mortality. However, these clinical variables lack significant sensitivity and specificity (66%-83%) compared to metabolomics data (Additional file 1: Table S6 vs. Table 3 and Additional file 1: Table S7).

#### The combination of metabolomics and clinical variables for predicting GOSE outcome at 3- and 12-months post-injury

SIMPLS analysis demonstrated that clinical variables could moderately improve the performance of metabolomics-based prediction models to prognosticate only GOSE outcome at 3 months and mortality (Additional file 1: Table S7). For the prognosis of GOSE outcome at 12 months, clinical variables were found to minimally improve the metabolomics models (data not shown). However, age was an important clinical predictor of outcome among clinical variables, with a high level of contribution to prediction models, particularly for mortality. Consequently, Marshall classification (3 months outcome) and GCS (12 months outcome) remain important clinical variables (Additional file 1: Table S8). Although SIMPLS and PLS-DA use different algorithms, the two approaches showed overall similar predictabilities when metabolites were used to prognosticate sTBI outcomes, with only slight differences (as shown in Additional file 1: Table S7 vs. Tables S4-6). Importantly, permutation tests (not shown) verified the predictabilities of metabolite-based prediction models and were used to help prevent overfitting of the data.

## Discussion

The current findings show that metabolite alterations on days 1 and 4 post-sTBI were highly predictive and well-correlated with GOSE unfavorable and favorable outcomes at 3 and 12 months and importantly, may also be used as a promising prognostic tool to predict the worst GOSE outcome, i.e., death. The metabolic biosignatures on day 4 post-injury were more predictive and significant than on day 1 to prognosticate 3- and 12-month outcomes. From a total of 160 metabolites, multivariate analysis revealed that several metabolites contributed to the separation of groups with unfavorable versus favorable outcome, implying fundamental metabolic alterations with sTBI that allows one to predict the outcome with high sensitivity, specificity, and AUC. The higher predictability of serum metabolic biosignatures on day 4 for the prognosis of outcomes may reflect the contribution of secondary brain injury (more likely reflected by day 4 metabolites) in addition to primary brain injury (reflected by day 1 metabolites) that correlate with outcome. Considerable metabolite alterations from day 1 to day 4 among patients with unfavorable outcomes compared to favorable outcomes may well reflect damage to the brain identified by these metabolite changes. The current study demonstrated that subtle changes in the metabolic profiles correlate with known and unknown pathophysiological pathways that can be applied to predict 3- and 12-month outcomes. Despite the higher predictability of metabolic biosignatures obtained by DI/LC–MS/MS for the prognosis of the outcome, the ^1^H-NMR platform was able to significantly predict the outcome. This capability of ^1^H-NMR, a much less sensitive analytical platform than DI/LC–MS/MS, reveals there is measurable early detectable metabolite alteration associated with outcomes. Importantly, a remarkable similarity was found for the trends in changes in metabolites measured by both methodologies, showing a high level of accuracy of quantification using two different analytical platforms.

Metabolomics appears to be superior compared to patients’ demographics, clinical features, and CT findings in predicting GOSE outcome at 3- and 12-months post-injury. Notably, the combination of metabolomics with clinical and CT variables enhanced the metabolomics prognostication of sTBI outcome in the early days post-injury, though clinical and CT data only improved the metabolomics prediction models for the prognosis of GOSE outcome at 3 months, not 12 months. The addition of age, GCS, hypoxemia, injury severity score, and Marshall CT classification enhanced the performance of metabolomics-based prediction of outcome. Our results were similar to the IMPACT and CRASH studies [27] in their use of age, GCS motor, pupillary reactivity, CT classification, EDH (epidural hematoma), tSAH (subarachnoid hemorrhage), hypoxia, and hypotension [27]. European Brain Injury Consortium Core Data (EBIC) and Traumatic Coma Data Bank (TCDB) studies identified age, GCS motor, pupillary reactivity, hypoxia, hypotension and CT classification as the most important predictors of 6- months outcomes using multivariate analysis (AUC 0.83–0.89) [28]. We showed that age and ISS are the most differentiating prognostic variables for mortality, while the IMPACT prediction model revealed age, GCS motor score, pupillary reactivity, hypoxia, hypotension, basal cisterns narrowing, midline shift and tSAH as the most predictive variables for 14-day mortality [29]. Using a multimodal approach, physiological (ICP, MAP, CPP and pbtO2) and biochemical (pyruvate, lactate, glycine, glutamate, and glucose) parameters could predict sTBI outcome with approximately 90% accuracy [30]. Our study also demonstrates the importance of multivariate predictive and machine learning-based models versus simplified methods to determine predictive metabolites. A Bayesian networks approach previously showed an improvement in prediction models using variables that were not predictive in simplified models [31]. PLS-DA and SIMPLS have shown the power of multivariate methods to explore big and complex datasets with many variables and relatively small sample sizes [32].

The current study suggests that, as previously described, increased lysoPCs in patients with the unfavorable outcome may be correlated with microvascular barrier disruption, promotion of oligodendrocyte demyelination and pericyte loss and with induced inflammation [33]. Increased stearic acid (C18) and its derivatives (stearic acid, oleic acid, linoleic acid) and lysoPCs in those with the unfavorable outcome may correlate with docosahexaenoic acid (DHA) metabolism, a highly enriched brain lipid [34]. Increased CSF levels of lysoPCs and PCs were previously observed in non-survivors and survivors [13] respectively, in mild TBI patients compared to non-concussed controls [14]. Thomas et al*.* (2022) showed that choline phospholipids such as lysophosphatidylcholines, ether phosphatidylcholines and sphingomyelins were the strongest predictors of TBI outcome in association with some amino acids and sugars. The logistic regression model including 19 metabolites had an AUC of 0.83 (95% CI 0.77–0.89) to predict patient outcomes in TBI [15]. Within one day post-sTBI, increased energy-related metabolites (lactate, glucose, and TCA cycle compounds) have been observed in patients with unfavorable outcomes. The lactate/pyruvate ratio is well-recognized as a predictor for the prognosis of brain injuries such as apoptosis, cerebral anoxia, and anaerobic metabolism [16, 17]. Mitochondrial dysfunction due to brain injury pathogenesis may appear with alterations in brain bioenergetics such as non-oxygen-requiring energy pathways and lipid peroxidation [35]. Even in the presence of oxygen, mitochondrial dysfunction can restrict the use of glucose efficiently, hence damaged brain can shift the aerobic metabolism toward the use of lactate directly as a new fuel [36]. There was a correlation between elevated lactate with unfavorable outcomes in TBI, in association with reduced cerebral blood flow (CBF), elevated ICP, and ischemia [16, 17]. In our study, increased tryptophan, kynurenine, tyrosine, phenylalanine, and glutamate on day 4 post-injury may intriguingly imply a correlation between excessive excitotoxicity mechanisms [18] and aromatic amino acid metabolism [19] with unfavorable outcomes. Increased quinolinic acid, the final product of the tryptophan-kynurenine pathway, has been associated with the inflammatory response due to the infiltration of macrophages and the activation of microglia in the CNS [20]. Higher level of quinolinic acid among sTBI patients with unfavorable outcomes and mortality may indicate the possibility of elevated macrophage-derived (or microglia-derived) excitotoxins in the contribution of secondary injury to poor outcome [20, 37]. In addition, day 4 increased NAA and phenylalanine, two well-known neurotransmitters in patients with unfavorable outcomes, may be associated with the alteration of osmolality and the catecholaminergic mechanism of injury [38]. The current study also shows the association of day 1 hyperglycemia and increased lactate with poor outcomes. Hyperglycemia and hyperlactatemia have been previously shown to be potential predictors for the prognosis of unfavorable TBI outcomes [39–41]. Mondello et al*.* (2022) found that 4 glycans were potentially correlated with TBI outcomes whereas two glycans significantly increased in patients with unfavorable outcomes (GOSE ≤ 4) and 2 glycans increased in patients with favorable outcomes. This study also showed a correlation between the increased glycans and mass lesions and decompressive craniectomy [42]. While in agreement with earlier studies, our findings add new information to understanding complex metabolic phenotypes for the prognosis of outcomes of sTBI that can help address possible therapeutic targets. Hence the correlation of increased lysoPCs, saturated and unsaturated long-chain fatty acids and aromatic amino acids with unfavorable outcomes suggest that anti-lysophosphatidylcholines and fatty acids may help to decrease the demyelination [43], neuroinflammation and enhance mitochondrial functions [44]. The proper balance of aromatic amino acids may enhance neurochemical repairs and cognitive performance and help decrease ICP [45].

Current findings provide novel evidence of targeted metabolomic profiling for the prognosis of short- and long-term GOSE outcome using serum samples at days 1 and 4 post-injury. A combination of amino acids, organic acids, fatty acids, and clinical and CT findings as variables were found to prognosticate the GOSE outcome of sTBI among adult patients quite well. The current results show that the metabolite changes associated with severe brain injury at days 1 and 4 can predict outcomes at 3 and 12 months. It is believed that daily serial sampling from day 1 to day 7 and day 14 or even later will provide more descriptions of metabolite changes and metabolic phenotypes to determine the outcome of sTBI in addition to understanding metabolite biomarker trajectories during the progression of brain damage, neuroplastic and therapeutic interventions.

Our findings support the notion that metabolomics is a powerful tool for understanding the complexity of the metabolic network of post-TBI pathogenic changes and that it may be useful to follow clinically relevant biomarkers that are monitored over time. These biomarkers may be used to evaluate the effectiveness of interventions or treatments supporting personalized treatments based on an individual's specific metabolic phenotype. Stimulation or inhibition of specific metabolic pathways may be the pharmacological approaches that can change the level of certain metabolites leading to reduced injury or a faster recovery resulting from utilizing specific selected treatment tools.

Targeted analysis of a limited number of metabolites in combination with more rapid (bedside-based) and improved analytical methods, especially mass spectrometry, will enable new tools to be utilized for discussions in clinical settings. This may dramatically reduce the time spent and the costs involved in making wise clinical decisions. The application of point-of-care devices and microfluidics can facilitate monitoring metabolite biomarkers for diagnostic, prognostic, and therapeutic purposes.

Limitations of the current study include: a relatively small sample size, not all patients had GOSE outcomes measured (several died or lost to follow-up), and there was a skewed cohort toward males (not uncommon in TBI studies). A larger and more gender-balanced cohort will be needed to validate our findings. The use of dichotomized GOSE, rather than using the full ordinal GOSE scale for our outcome models, was done because the small sample size would not allow the use of the full ordinal GOSE scale for statistical predictability. A larger study allowing the use of the full ordinal GOSE scale may allow for underestimating the role of other metabolites in the prediction of outcome and may help uncover any nonlinearity between metabolites and GOSE outcome [46]. Sliding dichotomous outcome assessment is an alternative to the traditional dichotomous outcome assessment that may be associated with an increase in precision and prevent the risk of making overgeneralizations. It can provide a more precise and comprehensive way to understand the complex relationships in sTBI prognosis in clinical trials. It can help researchers and clinicians better understand the diversity and individual differences leading to an improved prognosis of outcomes [24, 47].

It is controversial what role blood and blood product transfusion in trauma care plays in the metabolites found in serum. In this study, 15 patients had blood or blood product transfusion between 1 to 4 units. The transfusion was performed for 8 out of 15 patients after day 1 post-injury and for 2 patients after day 4 post-injury (thus not affecting measured metabolites), however, the impact on serum metabolites is uncertain for those samples collected after transfusion. Some patients were lost to follow-up in this study. How this affected the results of this study is unknown, however, further analysis of patient’s demographics and clinical symptoms on admission showed a random effect of those patients lost to follow-up (i.e., there was no systematic loss to follow-up noted). Despite these limitations, our study shows great promise in using metabolomics to evaluate sTBI, particularly for prognostic assessment. In this study, the majority of patients (56%, Additional file 1: Tables S9 and S10) with sTBI did not have polytrauma. However, we did not exclude polytrauma patients due to the small sample size. This may add some heterogeneity to the data, but we were still able to see significant differences in metabolites that correlate closely with GOSE outcome. There was no separate analysis of the patients with polytrauma versus the patients with an isolated head injury. Polytrauma poses a challenge to TBI biomarker discovery due to the complexity and heterogeneous nature of the injuries involved. Polytrauma in TBI is associated with heterogeneity in the injury profile due to different injury severity, including orthopedic injuries, thoracic trauma, and abdominal injuries. These heterogeneities may influence the release and potency of different TBI-specific biomarkers (including metabolites). Understanding the impact of polytrauma on the expression and kinetics of TBI biomarkers is crucial for effective research and monitoring [48]. Polytrauma associated with confounding factors can affect biomarkers that are strongly related to several disease processes such as inflammation or tissue damage and the interpretation of results in severe TBI research. These variables are not the primary focus of this study but may affect both biomarker levels and outcomes of interest. It is important to consider and address these factors to ensure the accuracy and reliability of biomarker testing in sTBI. These confounding factors include the various factors of TBI, such as the cause of the injury (accidental falls and motor vehicle accidents), the location of the injury, and the characteristics of the patient (age, sex, and pre-existing conditions). Time from injury is important to capture biomarker levels in TBI that change over time [49]. Certain biomarkers become more important during different stages of injury and recovery. Confounding factors such as comorbidities and co-occurring injuries can exacerbate TBI pathophysiology and alter the biomarker profiles. Finally, various factors unrelated to TBI, such as environmental exposures, genetic variations, and lifestyle (smoking, alcohol consumption, and diet) may also influence biomarker profiles [50].

Metabolic profiling of sTBI patient samples beyond the first 4 days may potentially enhance the predictability of metabolomics to prognosticate outcome and may provide more definitive information about molecular changes post-sTBI, especially in those who have a favorable outcome of sTBI. Also, applying an untargeted mass spectrometry approach may help identify more known and unknown metabolites that may be correlated with sTBI prognosis and help to define the mechanisms of injury more clearly in sTBI (for both primary and secondary injury).

In this study, the prognostication models showed highly predictive and significant separation between sTBI patients with unfavorable and favorable outcomes using serum metabolomics with remarkable similarities between two different metabolomics analytical platforms while the patient’s demographics and clinical variables were not strong independent predictors of GOSE outcome. Importantly, the information derived from metabolomics and prediction models may be used to stratify patients with sTBI that can be applied in future clinical trials, especially therapeutic trials as a means of prognostic enrichment. Targeted DI/LC–MS/MS (including multiple lipid metabolites) appears to be superior to ^1^H-NMR to predict sTBI outcome and this information may be useful for future studies.

## Conclusion

In summary, the best prognostic metabolomics models to predict GOSE outcomes at 3- and 12-months revealed increased glycolytic metabolites, hyperglycemia, and lactate on day 1, increased aromatic amino acids (tryptophan, tyrosine, and phenylalanine) on day 4, metabolites involved in excitotoxicity (increased glutamate), increased neuroinflammation metabolites (increased lysoPCs and kynurenine) on both days 1 and 4, increased neurobiomarkers (increased NAA and tyrosine), decreased ketone bodies, decreased urea cycle metabolites and degradation of branched-chain amino acids (BCAA) on day 4.

## Supplementary Information

**Additional file 1.** Includes clarification of methods as well as additional data tables and figures.

## Data Availability

Data are available on request.

## Abbreviations

H-NMR: Proton Nuclear Magnetic Resonance
ANN: Artificial neural network analysis
AUC: Area under the curve
CT scan: Computed tomography scan
DI/LC–MS/MS: Targeted direct infusion liquid chromatography-tandem mass spectrometry
GCS: Glasgow coma scale
GOS: Glasgow outcome scale
GOSE: Glasgow outcome scale extended
ISS: Injury severity score
LysoPCs: Lysophosphatidylcholines
NAA: N-Acetylaspartate
PCs: Phosphatidylcholines
PCA: Principal component analysis
PLS-DA: Partial least Square- Discriminant analysis
SIMPLS: Statistically inspired modification of partial least squares
sTBI: Severe traumatic brain injury
TBI: Traumatic brain injury
VIP: Variable important in the projection
