# Supplementary material for: Using metabolomics to predict severe traumatic brain injury outcome (GOSE) at 3 and 12 months
Source: Crit Care. 2023 Jul 22;27:295. doi: 10.1186/s13054-023-04573-9 (PMC10363297; doi:10.1186/s13054-023-04573-9)
Supplement: Supplementary file 1 — Additional file 1. Includes clarification of methods as well as additional data tables and figures. [file 13054_2023_4573_MOESM1_ESM.docx]

Using **metabolomics to predict severe traumatic brain injury outcome (GOSE) at 3-and 12 months**

Mohammad M. Banoei^1^ Ph.D., Chel Hee Lee^1^ Ph.D., James Hutchison^2^ M.D , William Panenka^3^ M.D., Cheryl Wellington^4^ Ph.D., David S. Wishart^5^ Ph.D., Brent W. Winston^1,6^ M.D., on behalf of the Canadian biobank and database for Traumatic Brain Injury (CanTBI) investigators*, the Canadian Critical Care Translational Biology Group (CCCTBG) and the Canadian Traumatic Brain Injury Research and Clinical Network (CTRC)

^1^ Department of Critical Care Medicine, University of Calgary, Alberta, Canada., [mmbanoei@ucalgary.ca](mailto:mmbanoei@ucalgary.ca).

^1^ Department of Critical Care Medicine, University of Calgary, Alberta, Canada., [chelhee.lee@ucalgary.ca](mailto:chelhee.lee@ucalgary.ca)

^2^Departments of Pediatrics and Critical Care and Neuroscience and Mental Health Research Program, SickKids and Interdepartmental Division of Critical Care and Institute for Medical Science, and the University of Toronto, Toronto, Ontario, Canada. Jamie.hutchison@sickkid.ca

^3^BC Mental Health and Substance Use Research Institute and the Department of Psychiatry, Faculty of Medicine, University of British Colombia. [will.panenka@ubc.ca](mailto:will.panenka@ubc.ca).

^4^Djavad Mowafaghian Centre for Brain Health, University of British Columbia, Canada; cheryl.wellington@ubc.ca

^5^Departments of Biological Sciences, Computing Sciences and Medicine and Dentistry, University of Alberta, Alberta, Canada. dwishart@ualberta.ca

^6^Departments of Critical Care Medicine, Medicine and Biochemistry and Molecular Biology, University of Calgary, Calgary, Alberta, Canada. Tel: +1(403) 220-4331, Fax: (403) 283-1267, [bwinston@ucalgary.ca](mailto:bwinston@ucalgary.ca).

Supplemental

**Address correspondence to:**

Brent W. Winston, MD, Departments of Critical Care Medicine, Medicine and Biochemistry and Molecular Biology, University of Calgary, Health Research Innovation Center (HRIC), Room 4C64, 3280 Hospital Drive N.W., Calgary, Alberta, Canada, T2N 4Z6.

Tel: (403) 220-4331

Fax: (403) 283-1267

Email: [bwinston@ucalgary.ca](mailto:bwinston@ucalgary.ca)

*The CanTBI Investigators are listed at the end of the supplemental material

Key Words: severe TBI, metabolomics, outcome prediction, prediction modelling

**Materials and Methods**

**Patients Participants**

A total of 466 adult and pediatric patients with mild, moderate and severe forms of TBI were entered into the CanTBI study and database. All patients were admitted to critical care units, and/or emergency departments and/or assessed in concussion clinics at participating centers. There were both pediatric and adult arms to the CanTBI study. In the adult arm, the inclusion criteria for adults severe TBI (sTBI) included:

- 1. Patient ≥18 yrs with acute severe TBI.
- 2. Glasgow coma scale (GCS) ≤ 8.
- 3. Patient had at least 1 research blood sample drawn within 24 hours + 6 hours from TBI.
- 4. Patient/substitute decision maker can speak and read English and/or French.
- 5. Patient/substitute decision maker has a fixed address.
- 6. Obtaining informed consent from patient or designated legal surrogate either directly or in a delayed fashion.

Exclusion criteria consisted of:

- 1. Patient had a severe neurodevelopmental disorder pre-injury.
- 2. Patient has a confirmed or suspected brain death at the time of enrollment determined by the attending physician.
- 3. Patient has a terminal illness, expected to live less than 12 months from TBI.
- 4. Patient has ongoing neurologic deficit from a previous TBI or other acquired brain injury (e.g. stroke).
- 5. Patient had a cardiac event that potentially caused a TBI.
- 6. Patient/substitute decision maker is unwilling to participate in study follow-up.

Biological samples including whole blood, serum, plasma, buffy coat, CSF and brain material from any biopsy or from operative procedure that were collected in the CanTBI study at various dates and times over 28 days (as per the CanTBI Protocol). All biological samples were collected and handled as per predefined CanTBI SOPs with the goal of sample collection to freezer within 2 hours. Demographic and clinical data were collected from individuals including age at the time of TBI, sex, cause of TBI, pre-hospital events, GCS score, Abbreviated Injury Score (AIS), Injury severity score (ISS), clinical monitoring, medication and medical interventions. Extra information such as socioeconomic status, education and past medical history (prior concussion, migraine, psychiatric history, neurological history) were collected and assessed by an expert team for relevance. Details of lab and neuroimaging and neurophysiology were documented. Entered patients in the study participated in a battery of questionnaires and performance-based cognitive and behavioral assessments that focused on global outcome, TBI-related symptoms and quality of life at time points predetermined based on the severity of the injury. Glasgow Outcome Scale Extended (GOSE)/ Extended Pediatric (GOS-EP) and Extended Pee Wee GOS (GOS-E P-WEE) for pediatric patients, Rivermead Post Concussion Symptom Questionnaire (RPSQ), Brief Test of Adult Cognition (BTACT), Pediatric Quality of Life Questionnaire (PedsQL), Health Behavior Inventory (HBI), and Patient-Reported Outcomes Measurement Information System (PROMIS) were the primary and supplementary TBI outcomes collected from patients. In this study we focused on GOSE outcome at 3- and 12-months post injury and mortality at 3 months.

**Proton Nuclear Magnetic Resonance (^1^H-NMR) Spectroscopy**

Untargeted one dimensional (1D) ^1^H-NMR spectroscopy was used to identify and quantify the serum metabolites on days 1 and 4 post-sTBI samples using a 600 MHz Bruker Ultrashield Plus NMR spectrometer (Bruker BioSpin Ltd., Canada) at the University of Calgary. To extract the metabolites, 200 μl of serum from each patient sample was ultrafiltered using 3 KDa NanoSep microcentrifuge filters that filters small molecules < 3 KDa for analysis. DSS (4,4-dimethyl-4-silapentane-1-sulfonic acid) was used as an internal reference compound to quantify individual compounds.(1) 10 µl sodium azide (NaN3) was added to all filtrates to inhibit bacteria growth in the sample. The final volume of samples was adjusted to 400 µl by adding D2O followed by adjusting pH to 7.0 ± 0.04 at room temperature.(2) The NMR spectroscopy was obtained in 1D spectra using the pre-saturation pulse sequence (noesypr1d) with an optimal water suppression program and a mixing time of 100 milliseconds (ms).(2, 3) NMR acquisition was shimmed and calibrated based on the DSS peak at 0.0 ppm when the half-height line width of the DSS peak being less than 1.5 Hz. NMR spectra were obtained using 1028 scans and the spectra were then zero-filled and Fourier transformed to 128K points. All NMR spectra were corrected for line broadening, phasing, baseline correction based on the DSS peak at 0.0 ppm using a Topspin software program (Bruker BioSpin Ltd., Canada).(4) ChenomX NMR Suite 7.1 software (ChenomX Inc., Edmonton, Alberta, Canada) was used to process and profile the NMR spectra for the identification and quantification of metabolites. In the processing module, NMR spectra were manually phased followed by baseline correction, and the water peak region was removed from the spectra. Processed NMR spectra were then transferred to the profiler module. Untargeted profiling was performed in a semi-manual approach to quantify metabolites based on the DSS concentration at mM or mg/dl. All spectra were randomly ordered for untargeted profiling to avoid progressive or systematic bias. See Table S3 for a list of metabolites quantified by ^1^H-NMR metabolites. For more details, we have previously described the ^1^H-NMR spectroscopy analysis.(5)

**Direct infusion/liquid chromatography tandem mass spectrometry (DI/LC-MS/MS)**

Targeted, quantitative DI/LC-MS/MS was performed on days 1 and 4 post-sTBI serum samples using an ABI 4000 Q-Trap (Applied Biosystems/MDS Sciex) mass spectrometer. A targeted list of metabolites used in this study consisted of 130 metabolites including lipids, amino acids, biogenic amines and organic acids plus other metabolites (see Table S2 for a list of quantified metabolites). Reverse-phase liquid chromatography-tandem Mass Spectrometry (LC-MS/MS) was used to quantify amino acids, biogenic amines, and organic acids. Direct infusion tandem mass spectrometry (DI-MS/MS) was applied to quantify glycerophospholipids (lysophosphatidylcholines (lysoPCs) and phosphatidylcholines (PCs), acylcarnitines (Cs), and sphingomyelins (SMs).

To quantify organic acids, 150 µl of ice-cold methanol was added to thawed 50 µl serum samples followed by adding 10 µl of isotope-labelled standards. To precipitate proteins, the mixtures were kept in -20 °C overnight, and were centrifuged at 13,000×g for 20 min. 50 µl of supernatant extracts were added to a 96-well plate followed by adding 3-nitrophenylhydrazine reagent and were incubated for 2 hours. Before LC-MS/MS, 2mg/ml of Butylated hydroxytoluene was added to the extract.

To quantify amino acids and lipids, 10 µl of samples were added to a 96-well plate and samples were dried using a nitrogen stream. Phenyl-isothiocyanate reagent was used to derivatize the compounds. Samples in the plate were incubated and dried using an evaporator. Extraction solvent (300 µl) was added to the samples followed by centrifugation to drive the analytes to the lower part of the 96-well plate. Formic acid (0.2%) in water and formic acid (0.2%) in acetonitrile was used in dilution. The isotope-labeled internal standards and other standards were used to quantify each metabolite in the list using multiple reaction monitoring (MRM) pairs.

For LC-MS/MS analyses, chromatography was performed using an Agilent reversed-phase Zorbax Eclipse XDB C18 column (3.0 mm × 100 mm, 3.5 μm particle size, 80 A pore size) with a Phenomenex (Torrance, CA, USA) Security Guard C18 pre-column (4.0 mm × 3.0 mm) was used to quantify the amino acids and biogenic amines. The parameter for chromatography was set up as follows: mobile phase A was 0.2% (v/v) formic acid in the water, and mobile phase B was 0.2% (v/v) formic acid in acetonitrile. The gradient parameters were t = 0 min, 0% B; t = 0.5 min, 0% B; t = 5.5 min, 95% B; t=6.5 min, 95% B; t = 7.0 min, 0% B; and t = 9.5 min, 0% B. The chromatography column was set as 50 ºC. 10 µl of samples were injected into the column with the flow rate at 300 µl/min. The organic acid chromatography was set up as follows, mobile phase A was 0.01% (v/v) formic acid in the water, and mobile phase B was 0.01% (v/v) formic acid in methanol. The gradient parameters were t = 0 min, 30% B; t = 2.0 min, 50% B; t = 12.5 min, 95% B; t=12.5 min, 100% B; t = 13.5 min, 100% B; and t = 13.6 min, and finally 30% B for 4.4 min. The column was set at 40 ºC. 10 ul of samples were injected into the column with flow rate at 300 µl/min.

For DI-MS/MS analyses, samples were directly injected into the mass analyzer from the autosampler. The mobile phase was set by mixing 60 µl of formic acid, 10 ml of water and 290 ml of methanol. The flow rate was t=0 min, 30 µl/min; t=1.6 min, 30 µl/min; t= 2.4 min, 200 µl/min; t=2.8 min, 200 ul/min and t= 3.0 min, 30 µl/min. 20 µl of samples were injected into MS. The lipid concentration was measured semi-quantitatively using a single point calibration of representative metabolites obtaining by a linear regression.

For quantification of metabolites, standard calibration (seven points) was obtained for each of the organic acids, amino acids, and biogenic amines. The signal ion intensity of metabolites was corrected to the corresponding internal standards followed by calculating the concentration using the quadric regression with a 1/x^2^ weighting

The first row of the plate is devoted to 1 blank, 3 zero samples, 1 standard, and 3 quality controls. The samples were delivered to the mass spectrometer using direct infusion.(5) MetIQ software was used to control the entire assay workflow, from sample registration to automated calculation of metabolite concentrations to the export of data into other data analysis programs. A targeted profiling scheme was used to quantitatively screen for known small molecule metabolites using multiple reaction monitoring, neutral loss, and precursor ion scans. Analyst 1.6.2 and MulitQuant 3.0.3 software was used for the quantification of metabolites concentration. Details of the DI/LC-MS/MS metabolite detection, measurement and analysis have been previously described in more detail.(5).

**Data Analysis**

PCA was performed initially to find the trends, similarity, clustering, and outliers (technical and biological outliers). PCA was performed as an unsupervised analysis to examine the metabolomics data before applying supervised analyses including partial least square discriminant analysis (PLS-DA), statistically inspired modification of partial least squares analysis (SIMPLS) and artificial neural network (ANN) analysis.

For MVA, the sensitivity, specificity, and AUC were calculated for PLS-DA models using prediction analysis and multivariate misclassification. Following a standard protocol, the prediction models were selected and verified based on performance parameters R^2^Y (or R2, goodness of fit), Q^2^Y (or Q2, goodness of prediction) and *p* value (level of significance) through a cross-validation (CV) method. CV was performed based on the leave-one-out cross validation (LOOCV) to assessing generalizability of the results using an independent data set. These parameters are assigned for assessing the reliability, predictability and significance level of a model.(6) The prediction models were built using the most differentiating metabolites based on a variable importance in projection (VIP) level >|1.0|. Additionally, in order to minimize the metabolites in a model but still be predictable, the best prediction models were selected according to the criteria including the highest Q^2^, with a significant p-value, with sensitivity and specificity > 85% and with an AUC > 0.90. This approach did not change the topmost important metabolites but dropped the less important metabolites in the prediction models. Permutation tests were performed using 200-times testing and this was applied to each prediction model to verify the Q^2^ value and help ensure the data was not overfit. Coefficient plots were applied to illustrate the most differentiating metabolites obtained by the prediction models (PLS-DA). The Coefficient plot, by default, displays the coefficients referring to scaled and centered data for a given response, with 95% confidence intervals derived from jack-knifing. Statistically inspired modification of PLS (SIMPLS), an alternative approach to PLS regression,(7) was performed to build prediction models using clinical data and for the combination of clinical and metabolite variables. Also, artificial neural network (ANN) and predictor screening analysis were applied to extract more information from metabolomics datasets and clinical variables as well as internal validation of prediction models obtained by PLS-DA and SIMPLS. ANN, as a supervised nonlinear approach, was used to classify metabolomics data particularly for model data where the relations or functions are not known. In this study, ANN was a suitable complementary method to PLS analysis due to identification of a subset of the variables with maximal explanatory power. ANN provided an interpretable description of biological data using prediction models obtained by training and validation subsets.(8) ANN was performed through launching two types of prediction models: training, and validation models using the most differentiating metabolites (VIP>|1.0|) obtained by PLS. Partition analysis (PA) was performed to find the relationship between the clinical variables and GOSE outcomes at 3 and 12 months. The algorithm of PA finds all possible splits of the clinical variables to best predict GOSE outcomes. PA can classify the patients using cutoff points of each clinical variable with either continuous or ordinal values. Cross-validation ANOVA (CV-ANOVA) and permutation test (200 times) analyses were performed as internal validation and to verify the predictability of the models.

**The prognosis of the unfavorable GOSE outcome cohort showed different predictabilities at 3 and 12 months.**

ANN revealed that the prediction of unfavorable GOSE outcome was more accurate (AUC>0.97) than the prediction of favorable GOSE outcome according to training and validation sets (Table S4). A higher level of predictability for the prognosis of unfavorable GOSE outcome was observed using day 4 samples and the DI/LC-MS/MS dataset compared to the day 1 dataset. Of note, the DI/LC-MS/MS dataset was better than the ^1^H-NMR dataset in predicting unfavorable GOSE outcome at 3 months. While the prognosis of favorable GOSE outcome was more predictive than the prognosis of unfavorable outcome at 12 months using both training and validation sets in ANN analysis of ^1^H-NMR dataset (Table S5). Further analysis showed that the prognosis of GOSE outcome at 12 months overall was slightly less predictive than the prognosis of GOSE outcome at 3 months (Tables S7-S8). Overall, the prognosis of 12-month GOSE outcome was less predictable than the prognosis of 3-month GOSE outcome.

**Identified, quantified metabolites using DI/LC-MS/MS and ^1^H-NMR.**

We were able to quantify 130 and 58 metabolites using targeted DI/LC-MS/MS and untargeted ^1^H-NMR, respectively (Tables S2-S3). The quantified metabolites by the DI/LC-MS/MS platform included 75 lipids (glycerophospholipids, acylcarnitines, sphingomyelins), 22 amino acids, 23 biogenic amines, 17 organic acids, and several compounds from different metabolite classes. In addition, the quantified metabolites by ^1^H-NMR included 22 amino acids, 20 organic acids, 4 sugars and 12 biogenic acids for a total of 58 metabolites. Though there are several common metabolites between DI/LC-MS/MS and ^1^H-NMR methods, the approaches to identify and quantify metabolites were completely different between the techniques. Both techniques were quantitative analyses in this study, but the quantification of metabolites was based on the ion intensities of metabolite fragmentations and the physical-chemistry of the hydrogen atom (proton ^1^H) in intact metabolites for DI/LC-MS/MS and ^1^H- NMR, respectively. Further analysis showed that 80% (24 out of 30) of the overlapping metabolites followed a similar trend of change that proves the accuracy of both techniques.

**Patients’ demographics, clinical information and CT findings between non-survivors and survivors’ cohorts.**

There was a significant difference in age and ISS between patients who died (n=21) and those who survived (n=23) at 3 months (Table S1), with older age and higher ISS associated with unfavorable outcome.

**Characterization of metabolite biosignature for the prognosis of GOSE outcome**

A large number of metabolites contributed to the highly predictive (Q^2^>0.5) and significant separation (AUC>0.99) between cohorts with unfavorable and favorable GOSE outcomes at 3 and 12 months as well as non-survivors vs. survivors at 3 months (Figs. S13-S15). Nonetheless, further analyses showed that one may be able to decrease the number of metabolites and still build reasonable predictive (Q^2^>0.4) and accurate (AUC>0.90) models as shown in tables S4-S6. As the metabolites used in the models are decreased, there is an associated lower sensitivity (<80), specificity (<80), and AUC (<0.75) (data not shown). The following metabolic biosignature characterizations are based on the best prediction models.

**3-month prognosis**. Unfavorable outcome was characterized by an increase in lysophosphatidylcholine (lysoPCs), propionic acid, stearic acid, oleic acid, linoleic acid, myristic acid, choline, glycerol, glucose, lactate, pyruvate, tryptophan, homocysteine, and ketone bodies (2-hydroxybutyric acid, acetoacetate, and acetone) on the 1^st^ day post-injury, while glutamate, phenylalanine, tyrosine, kynurenine, NAA, aspartate, and branched chained amino acids (valine, leucine, and isoleucine) increased from the 1^st^ day to the 4^th^ day post-injury (Figs. S5-S8).

**12-month prognosis**. Unfavorable outcome was characterized by an increase in lysophosphatidylcholines (lysoPCs), short chain acylcarnitines (ACs), palmitic acid, oleic acid, linoleic acid, lactate, gluconate, branched chain amino acids, carnitine, glycerol and alanine and a decrease in spermine, methionine-sulfoxide, glutamate, ketone bodies, hydroxyisovalerate compounds, and dimethylamine on day 1 after injury. On day 4 after injury there was an increase in lysoPCs, tryptophan, caproic acid, lauric acid, [lauroleic](https://en.wikipedia.org/w/index.php?title=9-Dodecenoic_acid&action=edit&redlink=1) acids, oleic acid, tyrosine, branched chain amino acids, and ornithine and a decrease in spermine, spermidine, PCs, most medium and long chain ACs and serotonin in patients with unfavorable outcome vs. the patients with favorable GOSE outcome at 12 months (Figs S9-S12).

**Prognosis of Mortality**. Non-survivors were characterized by an increase in glucose, PCs, long chain acylcarnitines (oleic acid, linoleic acid, palmitoleic acid, myristolinoleic acid, lauroleic acid, capric acid, and myristoleic acid), TCA cycle metabolites, tryptophan, tyrosine, and ketone bodies on days 1 and 4 post-injury. There was a decrease in short chain acylcarnitines, glutamine and betaine that were correlated with a non-survival outcome on day 4 post injury (Figs. S13-S16).

**References**

1. Wishart DS, Knox C, Guo AC, Eisner R, Young N, Gautam B, et al. HMDB: a knowledgebase for the human metabolome. Nucleic Acids Research. 2009;37(Database):D603-D10.

2. Weljie AM, Newton J, Mercier P, Carlson E, Slupsky CM. Targeted profiling: quantitative analysis of 1H NMR metabolomics data. Anal Chem. 2006;78(13):4430-42.

3. Nicholson JK, Foxall PJ, Spraul M, Farrant RD, Lindon JC. 750 MHz 1H and 1H-13C NMR spectroscopy of human blood plasma. Anal Chem. 1995;67(5):793-811.

4. Weljie AM, Dowlatabadi R, Miller BJ, Vogel HJ, Jirik FR. An Inflammatory Arthritis-Associated Metabolite Biomarker Pattern Revealed by1H NMR Spectroscopy. Journal of Proteome Research. 2007;6(9):3456-64.

5. Banoei MM, Vogel HJ, Weljie AM, Yende S, Angus DC, Winston BW. Plasma lipid profiling for the prognosis of 90-day mortality, in-hospital mortality, ICU admission, and severity in bacterial community-acquired pneumonia (CAP). Critical care (London, England). 2020;24(1):461.

6. Eriksson L, Trygg J, Wold S. CV-ANOVA for significance testing of PLS and OPLS® models. Journal of Chemometrics. 2008;22(11-12):594-600.

7. de Jong S. SIMPLS: An alternative approach to partial least squares regression. Chemometrics and Intelligent Laboratory Systems. 1993;18(3):251-63.

8. Goodacre R, Vaidyanathan S, Dunn WB, Harrigan GG, Kell DB. Metabolomics by numbers: acquiring and understanding global metabolite data. Trends Biotechnol. 2004;22(5):245-52.

| GOSE outcome 3-month | GOSE 1  (non-survivors) | GOSE >1  (survivors) | *p-*value |
| --- | --- | --- | --- |
| Sex (Male/Female) | 17/4 | 19/4 | 0.978 |
| Age (mean ± SD) | 61.5 ± 18.6 | 42.8 ± 20.4 | 0.005 |
| Weight (mean ± SD) | 90.6 ± 19.5 | 83.4 ± 20.9 | 0.197 |
| Severity (mean ± SD) | 56.5 ± 22.6 | 35.1 ± 12.6 | <0.0001 |
| Admission-type  ER  Hospital Ward  ICU | 8 (38%)  0  12 (57%) | 7 (30%)  0  16 (69.5%) | 0.213 |
| Hypoxia (Yes/No)* | 4/15 | 4/16 | 0.374 |
| Intubated (Yes/No)* | 11/9 | 17/4 | 0.650 |
| Hypotension (Yes/No)* | 3/14 | 2/18 | 0.844 |
| Paralytic-AGT (Yes/No)* | 9/10 | 13/8 | 0.837 |
| Loss Consciousness* | 12/4 | 18/2 | 0.250 |
| Location of Injury ^ŧ^ |  |  | 0.611 |
| Type of Injury ^ŧ^ |  |  | 0.105 |
| GCS (total) (mean ± SD) | 5.5 ± 2.2 | 5.2 ± 2.2 | 0.732 |
| GCS-Motor  GCS-Eye (mean ± SD)  GCS-Verbal | 3.2 ± 1.8  1.4 ± 0.88  1.25 ± 0.85 | 2.4 ± 2.0  1.5 ± 1.1  0.85 ± 0.57 | 0.241  0.878  0.204 |
| GCS 3-4  GCS 5-6 (mean ± SD)  GCS 7-8 | 8 (38%)  4 (19%)  8(38%) | 12 (52%)  1 (4.3%)  10 (43.4%) | 0.491 |
| CT Findings**  Diffuse Axonal Injury (yes/no)  Mid Shift (yes/no)  Skull Fracture (yes/no)  Cerebral Edema (yes/no)  Contusion (yes/no)  Intracranial Hemorrhage (yes/no)  Epidural Hemorrhage (yes/no)  Subdural Hemorrhage (yes/no)  Arachnoid Hemorrhage (yes/no)  Marshall Score  I  II  III  IV  V | 3/13  4/11  12/4  4/12  8/8  11/5  0/6  13/3  15/1  0  10  3  1  2 | 2/14  6/9  10/6  2/14  7/9  9/7  3/13  11/5  12/4  0  9  2  2  3 | 0.715  0.803  0.624  0.380  0.609  0.669  0.251  0.528  0.098  0.711 |

**Table S1.** Patients’ demographics, clinical information and CT findings between non-survivor and survivor cohorts. There were no patients with a GOSE of 2 so the second column represents all survivors. *Shows the number of patients that had intubation and mentioned physiological conditions in each non-survivor and survivor group. ŧ the details have not been shown due to several conditions and table limitation. ** shows the number of patients that had brain damage captured on CT for non-survivor and survivor group. p-value < 0.05 was considered significant.

|  | **Metabolite** |  | **Metabolite** |  | **Metabolite** |
| --- | --- | --- | --- | --- | --- |
| 1 | Asymmetric dimethylarginine | 45 | C3:1 (Propenoylcarnitine) | 89 | PC acyl-alkyl (ae) C40:6 |
| 2 | total dimethylarginine | 46 | C3 (Propionylcarnitine) | 90 | PC ae C36:0 |
| 3 | alpha-Aminoadipic acid | 47 | C4:1 (Butenylcarnitine) | 91 | SM (OH) C14:1 |
| 4 | Creatinine | 48 | C4 (butyrylcarnitine) | 92 | SM (OH) C16:1 |
| 5 | Dopamine | 49 | C3-OH (hydroxyPropionylcarnitine) | 93 | SM (OH) C22:1 |
| 6 | Kynurenine | 50 | C5:1 (Tiglylcarnitine) | 94 | SM (OH) C22:2 |
| 7 | Methionine sulfoxide | 51 | C5 (Valerylcarnitine) | 95 | SM (OH) C24:1 |
| 8 | Hydroxyproline (t4-OH-Pro) | 52 | C4-OH (C3-DC) (Hydroxybutyrylcarnitine) | 96 | SM C16:0 |
| 9 | Phenylethylamine | 53 | C6:1 (Hexenoylcarnitine) | 97 | SM C16:1 |
| 10 | Putrescine | 54 | C6 (C4:1-DC) (Hexanoylcarnitine) | 98 | SM C18:0 |
| 11 | Sarcosine | 55 | C5-OH (C3-DC-M) (hydroxyvalerylcarnitine) | 99 | SM C18:1 |
| 12 | Serotonin | 56 | C5:1-DC (Glutaconylcarnitine) | 100 | SM C20:2 |
| 13 | Spermidine | 57 | C5-DC (C6-OH)(Glutarylcarnitine) | 101 | lysoPC a C14:0 |
| 14 | Spermine | 58 | C8 (Octanoylcarnitine) | 102 | lysoPC a C16:0 |
| 15 | Taurine | 59 | C5-M-DC (methylglutarylcarnitine) | 103 | lysoPC a C16:1 |
| 16 | Tyramine | 60 | C9 (Nonaylcarnitine) | 104 | lysoPC a C17:0 |
| 17 | Alanine | 61 | C7-DC (pimelylcarnitine) | 105 | lysoPC a C18:0 |
| 18 | Arginine | 62 | C10:2 (decadienylcarnitine) | 106 | lysoPC a C18:1 |
| 19 | Asparagine | 63 | C10:1 (Decenoylcarnitine) | 107 | lysoPC a C18:2 |
| 20 | Aspartate | 64 | C10 (Decanoylcarnitine) | 108 | lysoPC a C20:3 |
| 21 | Citrulline | 65 | C12:1 (Dodecenoylcarnitine) | 109 | lysoPC a C20:4 |
| 22 | Glutamate | 66 | C12 (dodecanoylcarnitine) | 110 | lysoPC a C24:0 |
| 23 | Glutamine | 67 | C14:2 (Tetradecadienylcarnitine) | 111 | lysoPC a C26:0 |
| 24 | Glycine | 68 | C14:1 (tetradecenoyl carnitine) | 112 | lysoPC a C26:1 |
| 25 | Histidine | 69 | C14 (tetradecanoylcarnitine) | 113 | lysoPC a C28:0 |
| 26 | Isoleucine | 70 | C12-DC (dodecanedioylcarnitine) | 114 | lysoPC a C28:1 |
| 27 | Leucine | 71 | C14:2-OH (hydroxytetradecadienylcarnitine) | 115 | Lactic acid |
| 28 | Lysine | 72 | C14:1-OH (Hydroxytetradecenoyl carnitine) | 116 | beta-Hydroxybutyric acid |
| 29 | Methionine | 73 | C16:2 (Hexadecadienylcarnitine) | 117 | alpha-Ketoglutaric acid |
| 30 | Ornithine | 74 | C16:1 (Hexadecenoylcarnitine) | 118 | Citric acid |
| 31 | Phenylalanine | 75 | C16 (Hexadecanoylcarnitine) | 119 | Butyric acid |
| 32 | Proline | 76 | C16:1-OH (Hydroxyhexadecenoylcarnitine) | 120 | HPHPA |
| 33 | Serine | 77 | C16-OH (hydroxyhexadecanoylcarnitine) | 121 | para-Hydroxyhippuric acid |
| 34 | Threonine | 78 | C18:2 (Octadecadienylcarnitine) | 122 | Succinic acid |
| 35 | Tryptophan | 79 | C18:1 (Octadecenoylcarnitine) | 123 | Fumaric acid |
| 36 | Tyrosine | 80 | C18 (Octadecanoylcarnitine) | 124 | Pyruvic acid |
| 37 | Valine | 81 | C18:1-OH (Hydroxyoctadecenoylcarnitine) | 125 | Isobutyric acid |
| 38 | Betaine | 82 | PC diacyl (aa) C36:6 | 126 | Hippuric acid |
| 39 | Choline | 83 | PC aa C32:2 | 127 | Methylmalonic acid |
| 40 | Creatine | 84 | PC aa C38:0 | 128 | Homovanillic acid |
| 41 | Methylhistidine | 85 | PC aa C38:6 | 129 | Indole acetic acid |
| 42 | Homocysteine | 86 | PC aa C40:1 | 130 | Uric acid |
| 43 | C0 (Carnitine) | 87 | PC aa C40:2 |  |  |
| 44 | C2 (Acetylcarnitine) | 88 | PC aa C40:6 |  |  |

**Table S2**. A full list of all quantified metabolites using DI/LC-MS/MS.

|  | **Metabolite** |  | **Metabolite** |
| --- | --- | --- | --- |
| 1 | 2-Aminobutyrate | 30 | Glutamate |
| 2 | 2-Hydroxybutyrate | 31 | Glutamine |
| 3 | 2-Hydroxyisovalerate | 32 | Glycerol |
| 4 | 2-Oxoglutarate | 33 | Glycine |
| 5 | 2-Oxoisocaproate | 34 | Histidine |
| 6 | 3-Hydroxybutyrate | 35 | Hypoxanthine |
| 7 | 3-Hydroxyisovalerate | 36 | Isobutyrate |
| 8 | 3-Methyl-2-oxovalerate | 37 | Isoleucine |
| 9 | 4-Hydroxybutyrate | 38 | Isopropanol |
| 10 | Acetate | 39 | Lactate |
| 11 | Acetoacetate | 40 | Leucine |
| 12 | Acetone | 41 | Lysine |
| 13 | Adipate | 42 | Mannose |
| 14 | Alanine | 43 | Methionine |
| 15 | Arginine | 44 | N-Acetylaspartate |
| 16 | Asparagine | 45 | N-Acetyl tyrosine |
| 17 | Aspartate | 46 | O-Phosphocholine |
| 18 | Betaine | 47 | Ornithine |
| 19 | Carnitine | 48 | Phenylalanine |
| 20 | Choline | 49 | Proline |
| 21 | Citrate | 50 | Pyruvate |
| 22 | Creatine | 51 | Serine |
| 23 | Creatinine | 52 | Succinate |
| 24 | Dimethyl sulfone | 53 | Taurine |
| 25 | Dimethylamine | 54 | Threonine |
| 26 | Formate | 55 | Tyrosine |
| 27 | Fumarate | 56 | Urea |
| 28 | Gluconate | 57 | Valine |
| 29 | Glucose | 58 | Beta-Alanine |

**Table S3**. A list of all quantified metabolites using ^1^H-NMR.


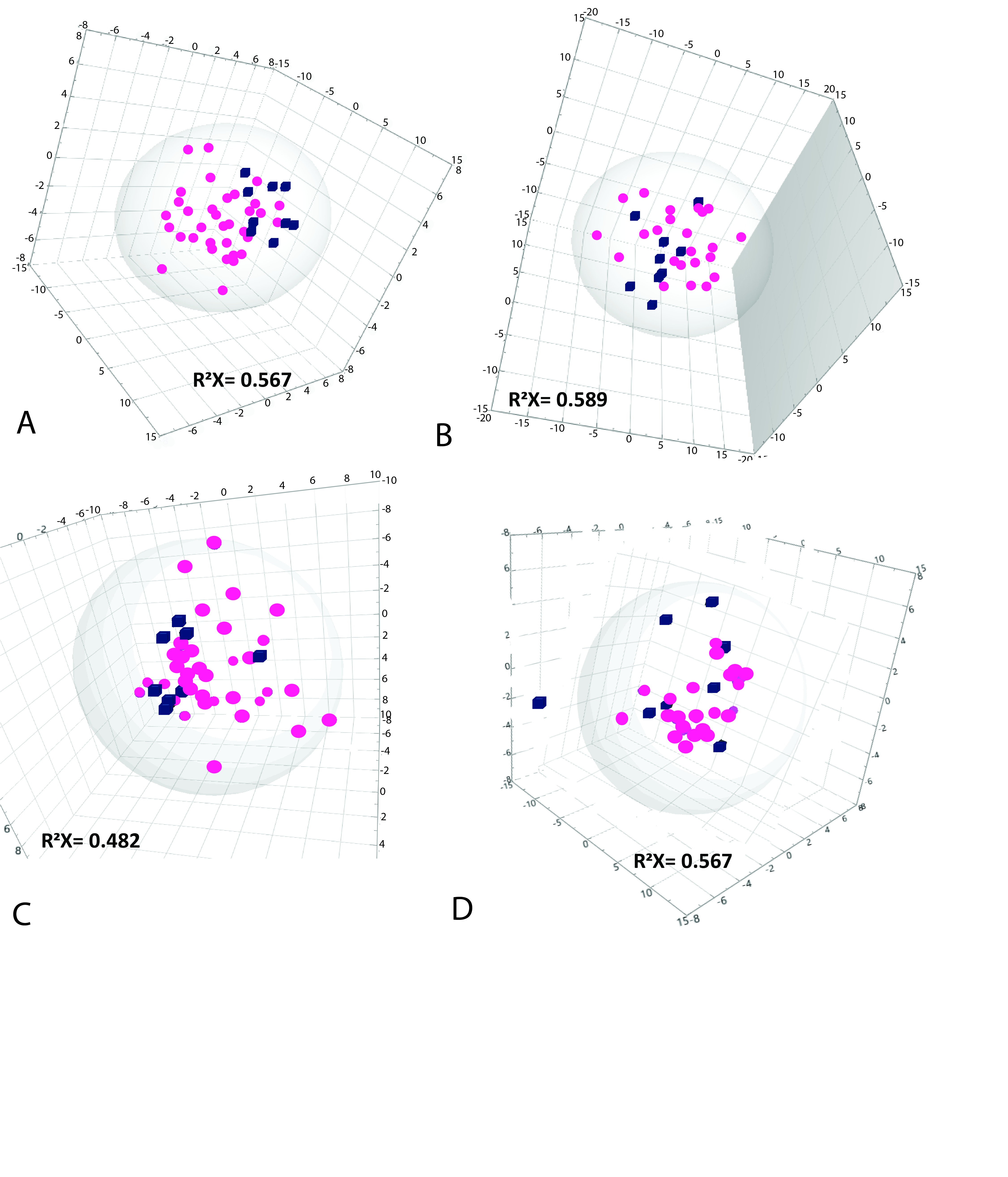


**Figure S1**. PCA scatterplot analysis shows a high variability of metabolic biopattern among 3-month unfavorable (●) and favorable (⯀) outcome cohorts: A: DI/LC-MS/MS day 1 (R^2^X = 0.567), B: DI/LC-MS/MS day 4 (R^2^X = 0.589), C: ^1^H-NMR Day 1 (R^2^X = 0.482), and D: ^1^H-NMR Day (R^2^X = 0.567). X-, Y- and Z-axis reflect PC1, PC2 and PC3, respectively. Clearer grouping is observed between the two cohorts using metabolic profiling on day 1 and day 4 post-injury based on the DI/LC-MS/MS than that of ^1^H-NMR. The highlighted circle reflects the Hoteling T^2^ 95% confidence interval.


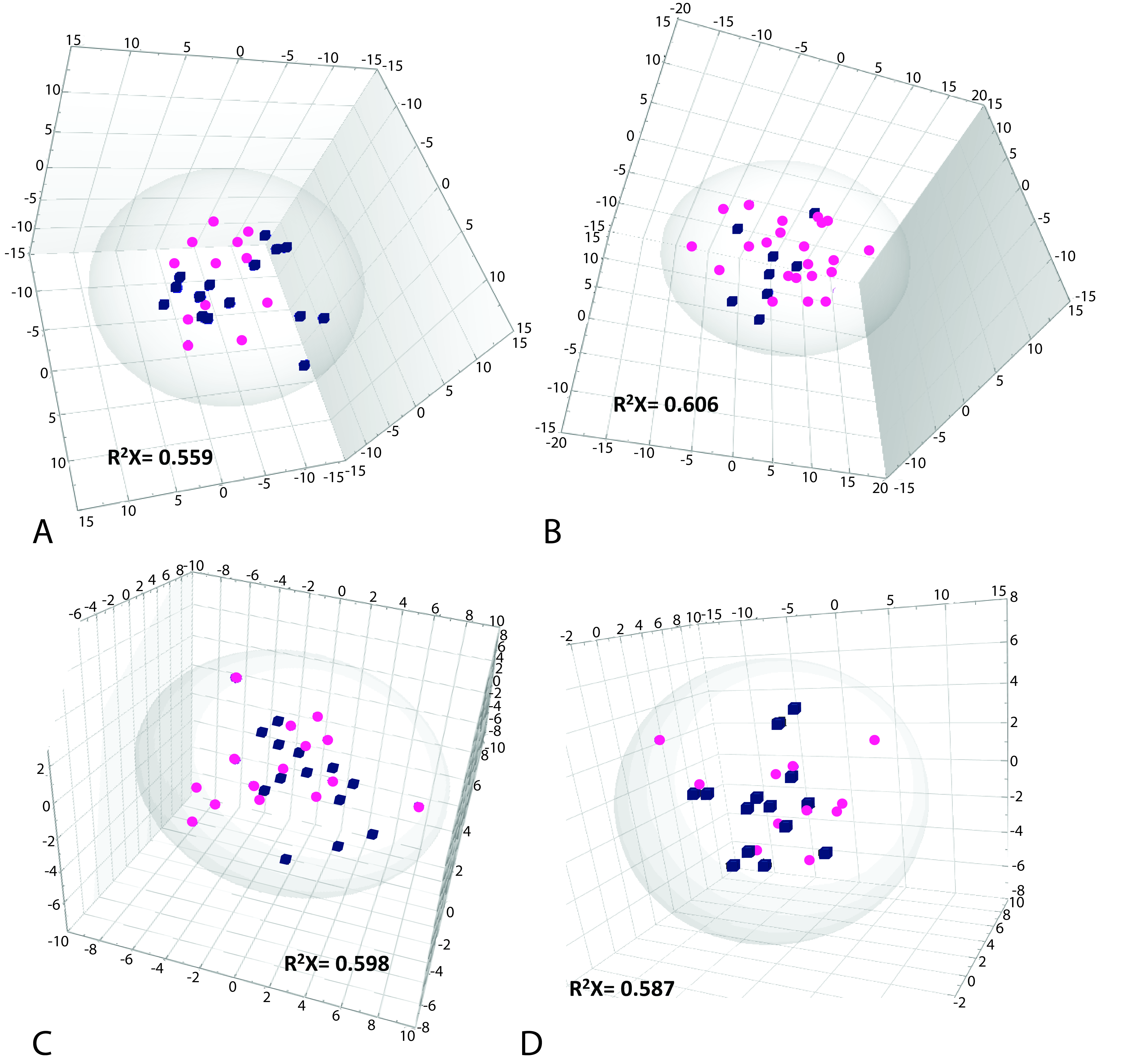


**Figure S2**. PCA scatterplot analysis shows a high variability of metabolic biopattern among 12-month unfavorable (●) and favorable (⯀) GOSE outcome cohorts: A: DI/LC-MS/MS day 1 (R^2^X = 0.559), B: DI/LC-MS/MS day 4 (R^2^X = 0.606), C: ^1^H-NMR Day 1 (R^2^X = 0.598), and D: ^1^H-NMR day 4 (R^2^X = 0.587). X-, Y- and Z-axis reflect PC1, PC2 and PC3, respectively. PCA plots show a better grouping among the two cohorts on day 4 post-injury data based on the DI/LC-MS/MS analysis compared to day 1 post-injury DI/LC-MS/MS analysis and compared to the ^1^H-NMR analysis. The highlighted circle reflects the Hoteling T^2^ 95% confidence interval.

**
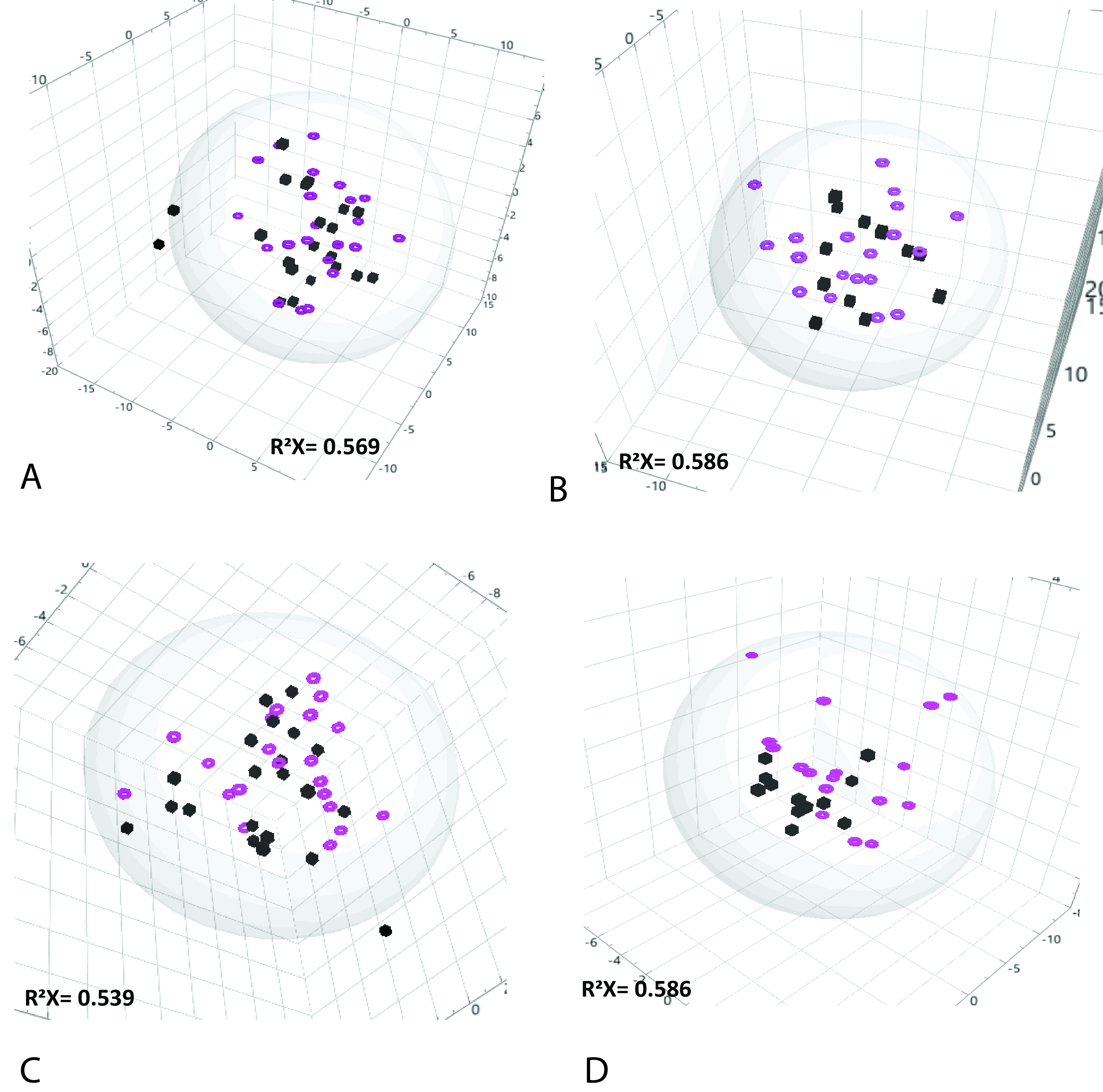
**

**Figure S3**. PCA scatterplot analysis shows a high variability of metabolic biopattern among patients who died (non-survivors) (●) and those who survived (⯀) at 3 months: A: DI/LC-MS/MS day 1 (R^2^X = 0.569), B: DI/LC-MS/MS day 4 (R^2^X = 0.586), C: ^1^H-NMR Day 1 (R^2^X = 0.539), and D: ^1^H-NMR Day 4 (R^2^X = 0.586). X-, Y- and Z-axis reflect PC1, PC2 and PC3, respectively. The highlighted circle reflects the Hoteling T^2^ 95% confidence interval.





**Figure S4.** PLS-DA scatterplots show a good discrimination between unfavorable (●) and favorable (⯀) GOSE outcome cohorts at 3 months **on day 1 post-injury**. **GOSE-3 months.** **A**: DI/LC-MS/MS, **B**: ^1^H-NMR. The predictability of separation of the two cohorts is higher for the DI/LC-MS/MS data (Q^2^= 0.393) compared to ^1^H-NMR (Q^2^= 0.21). So, the separation of the two cohorts is more predictive using metabolic profiling on day 1 of DI/LC-MS/MS than ^1^H-NMR. **GOSE-12 months.** **C**: DI/LC-MS/MS, **D:** ^1^H-NMR. The predictability of separation of the two cohorts is higher for the DI/LC-MS/MS data (Q^2^= 0.552) compared to ^1^H-NMR (Q^2^= 0.443) for GOSE at 12 months. **Mortality outcome,** **E:** DI/LC-MS/MS using 31 metabolites, **F:** ^1^H-NMR using 17 metabolites. The predictability of separation of the two cohorts is higher for the DI/LC-MS/MS data (Q^2^= 0.492) compared to the ^1^H-NMR data (Q^2^= 0.298) for mortality (non-survivors (●) vs survivors (⯀)) at 3 months. Separation of the two cohorts is more predictive using metabolic profiling on day 1 data using DI/LC-MS/MS than it is using ^1^H-NMR data at 3 months. The highlighted circle reflects the Hoteling T^2^ 95% confidence interval.


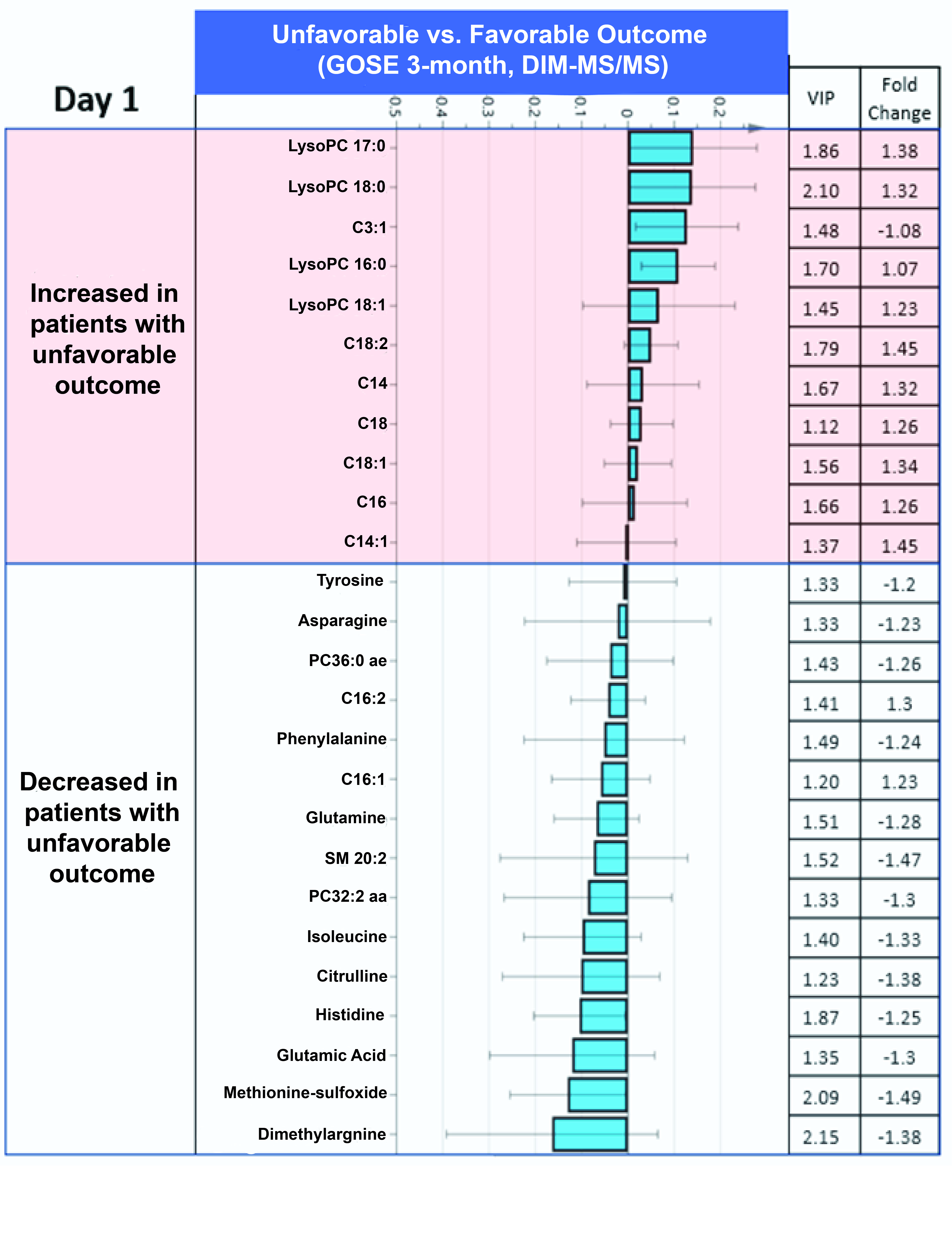


**Figure S5A.** The coefficient plot illustrates relative concentration correlation of the metabolite alterations between the two cohorts with unfavorable and favorable GOSE outcome at 3 months on day 1 post-injury samples based on the DI/LC-MS/MS dataset. VIP score and fold change are also displayed for each metabolite.

| **GOSE 3-month (Day 1) DIMS-MS** | | | | | |
| --- | --- | --- | --- | --- | --- |
| **Name** | **Mean (SD) of Favorable outcome (µM)** | **Mean (SD) of Unfavorable Outcome (µM)** | **p-value** | **Fold Change** | **Unfavorable/**  **Favorable** |
| LYSOC17:0 | 0.530 (0.091) | 0.732 (0.241) | 0.0003 | 1.38 | UP |
| LYSOC16:0 | 38.088 (9.033) | 48.442 (14.023) | 0.0422 | 1.27 | Up |
| LYSOC18:0 | 8.556 (2.083) | 12.521 (4.367) | 0.0662 (W) | 1.32 | Up |
| C18 | 0.024 (0.005) | 0.032 (0.012) | 0.0662 (W) | 1.32 | Up |
| C18:2 | 0.032 (0.010) | 0.046 (0.023) | 0.0295 (W) | 1.45 | Up |
| Histidine | 96.527 (22.009) | 77.035 (18.290) | 0.009 | -1.25 | Down |
| Glutamine | 467.018 (72.297) | 364.854 (105.588) | 0.0092 | -1.28 | Down |
| Methionine | 18.537 (2.669) | 15.130 (5.773) | 0.0152 | -1.23 | Down |
| Phenylalanine | 77.266 (20.639) | 62.451 (16.749) | 0.0292 | -1.24 | Down |
| Glutamic acid | 65.982 (21.544) | 50.805 (17.553) | 0.0327 | -1.3 | Down |
| Tyrosine | 35.836 (7.295) | 29.787 (7.877) | 0.0433 | -1.2 | Down |
| Methionine-sulfoxide | 0.901 (0.302) | 0.605 (0.256) | 0.0065 (W) | -1.49 | Down |
| Isoleucine | 63.284 (16.785) | 47.666 (22.718) | 0.0180 (W) | -1.33 | Down |
| Asparagine | 30.707 (6.731) | 24.935 (9.329) | 0.0180 (W) | -1.23 | Down |
| Threonine | 72.222 (15.831) | 63.244 (37.829) | 0.0273 (W) | -1.14 | Down |
| Leucine | 130.653 (38.222) | 103.716 (55.160) | 0.0466 (W) | -1.26 | Down |
| PC322AA | 3.378 (1.151) | 2.607 (0.821) | 0.0466 (W) | -1.3 | Down |
| SM20:2 | 0.900 (0.454) | 0.611 (0.239) | 0.0479 (W) | -1.47 | Down |


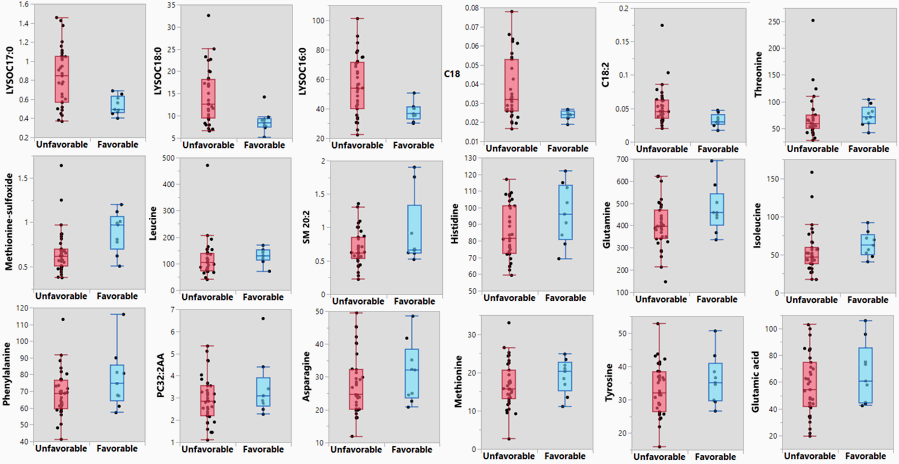


**Fig S5B**. Univariate analysis (t-test analysis) shows the significant (p< 0.05) metabolites between the two cohorts with unfavorable and favorable GOSE outcome at 3 months on day 1 post-injury samples based on the DI/LC-MS/MS dataset. The lower plot displays the same metabolites using dot and whisker plots showing the specific concentrations of each of the metabolites. Y-axis shows concentration in µM. (W) p-value is calculated by the Wilcoxon Mann Whitney test, the rest of the p-value are calculated with t-test.

**
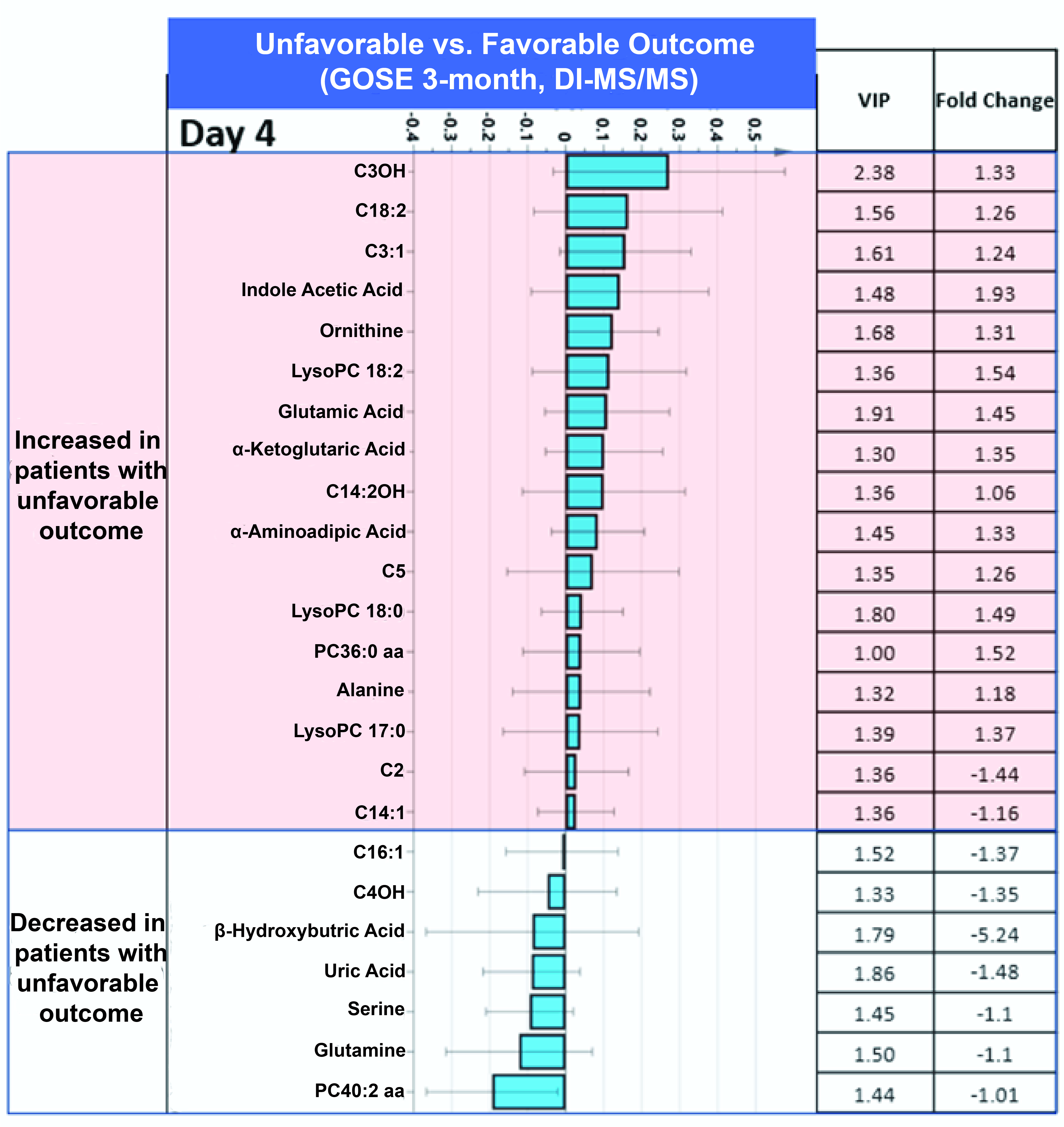
**

**Figure S6A.** The coefficient plot illustrates the relative concentration correlation of the metabolite alterations between the two cohorts with unfavorable and favorable GOSE outcome at 3 months on day 4 post-injury samples based on the DI/LC-MS/MS dataset. VIP score and fold change are also displayed for each metabolite.

| **GOSE 3-month (Day 4) DIMS-MS** | | | | | |
| --- | --- | --- | --- | --- | --- |
| **Name** | **Mean (SD) of Favorable (**µM.) | **Mean (SD) of Unfavorable (**µM.) | **p-value** | **Fold Change** | **Unfavorable/**  **Favorable** |
| Uric acid | 174.750 (59.974) | 102.108 (43.449) | 0.0009 | 1.71 | Down |
| Glutamine | 440.799 (90.105) | 354.923 (79.954) | 0.0169 | 1.24 | Down |
| Serine | 93.096 (19.529) | 74.917 (17.059) | 0.0181 | 1.24 | Down |
| SM 22:2 OH | 5.905 (0.508) | 5.177 (1.256) | 0.0296 | 1.14 | Down |
| Betaine | 33.416 (14.184) | 24.632 (8.770) | 0.0475 | 1.36 | Down |
| Glycine | 183.912 (42.079) | 155.385 (55.625) | 0.0481 (W) | 1.18 | Down |
| C14:1 | 0.096 (0.030) | 0.075 (0.043) | 0.0481 (W) | 1.27 | Down |
| C16:1 | 0.047 (0.027) | 0.031 (0.012) | 0.0481 (W) | 1.52 | Down |
| PC36:0 aa | 2.919 (0.693) | 3.979 (1.686) | 0.0197 | -1.36 | UP |


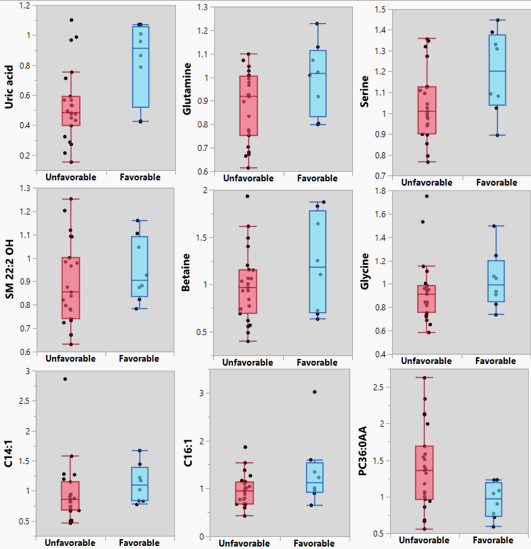


**Fig S6B**. Univariate analysis (t-test analysis) shows the significant (p< 0.05) metabolites between the two cohorts with unfavorable and favorable GOSE outcome at 3 months on day 4 post-injury samples based on the DI/LC-MS/MS dataset. The lower plot displays the same metabolites using dot and whisker plots showing the specific concentrations of each of the metabolites. Y-axis shows concentration in µM. (W) p-value is calculated by the Wilcoxon Mann Whitney test, the rest of the p-value are calculated with t-test.


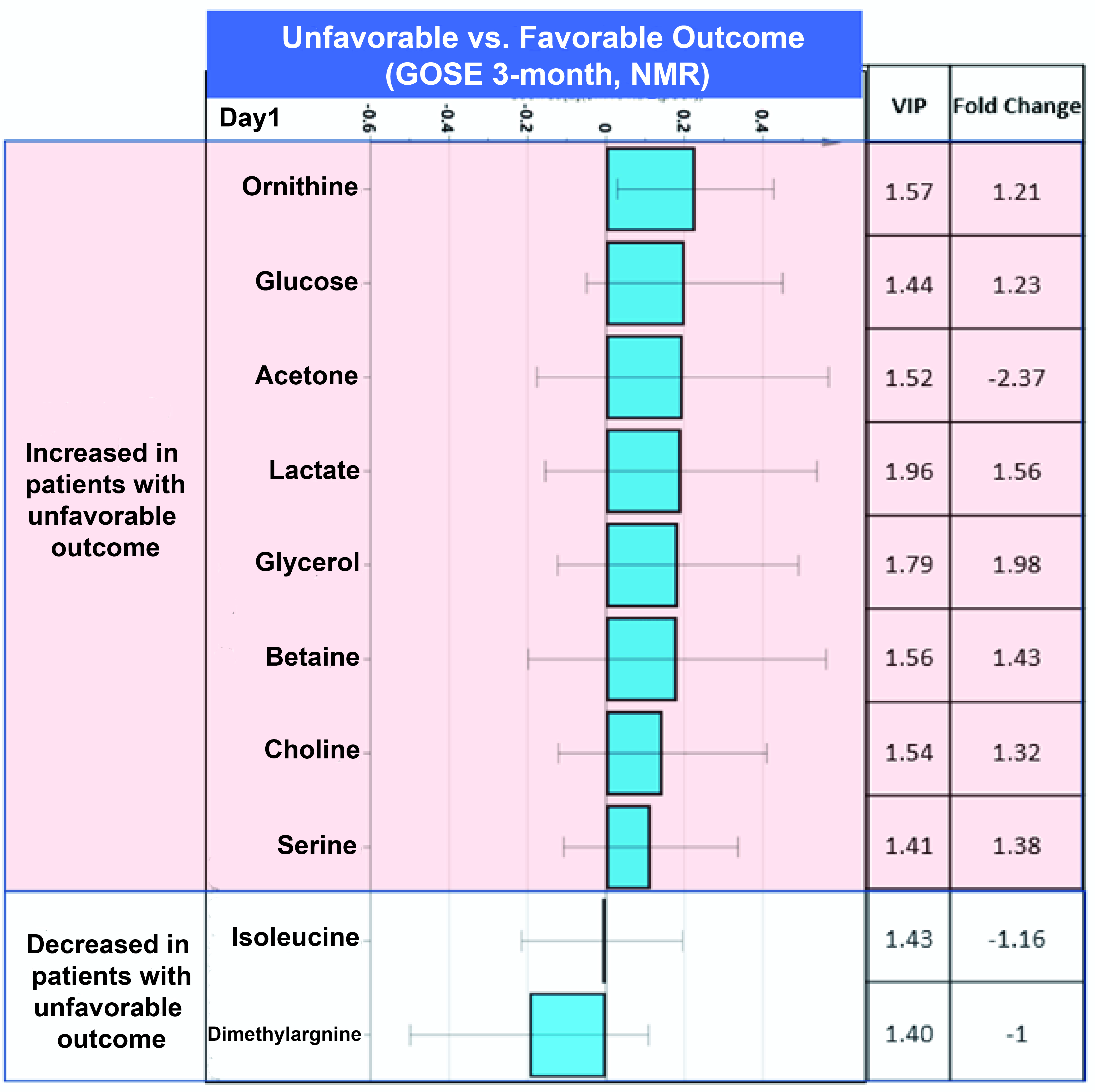


**Figure S7A.** The coefficient plot illustrates the relative concentration correlation of the metabolite alterations between the two cohorts with unfavorable and favorable GOSE outcome at 3 months on day 1 post-injury samples based on the ^1^H-NMR dataset. VIP score and fold change are also displayed for each metabolite. Only metabolites with a VIP score of > 1 regardless of sign are shown.

| GOSE 3-month (Day 1) NMR | | | | | |
| --- | --- | --- | --- | --- | --- |
| Name | **Mean (SD) of Favorable**  (µM) | **Mean (SD) of Unfavorable**  **(**µM) | **p-value** | **Fold Change** | **Unfavorable/**  **Favorable** |
| Glycerol | 2.583 (1.228) | 5.102 (3.593) | 0.0115 (W) | -1.98 | Up |
| Lactate | 30.611 (8.837) | 47.671 (19.590) | 0.0138 (W) | -1.56 | Up |
| Serine | 1.624 (0.463) | 2.234 (1.027) | 0.0232 (W) | -1.38 | Up |
| Glycine | 1.903 (0.440) | 2.716 (1.376) | 0.0345 (W) | -1.43 | Up |
| Betaine | 0.815 (0.323) | 1.169 (0.619) | 0.0402 (W) | -1.43 | Up |
| Choline | 0.144 (0.034) | 0.190 (0.065) | 0.0402 (W) | -1.32 | Up |


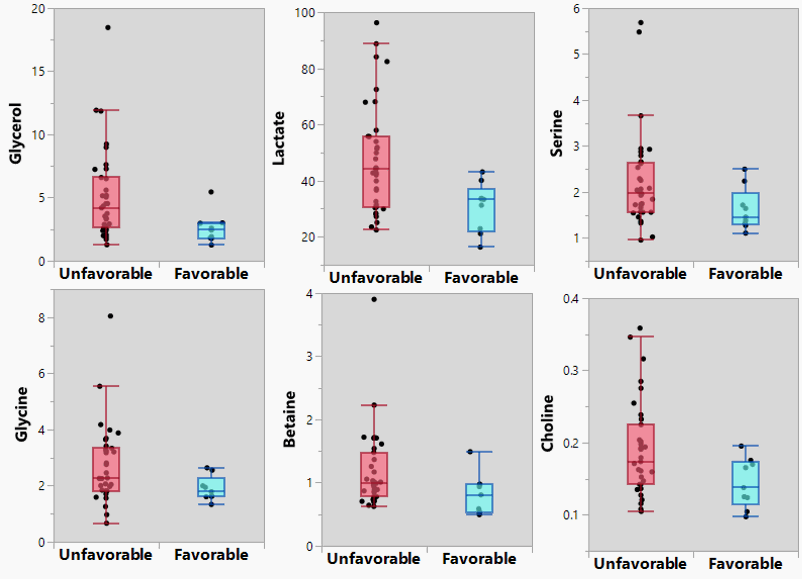


**Fig S7B**. Univariate analysis (t-test analysis) shows the significant (p< 0.05) metabolites between the two cohorts with unfavorable and favorable GOSE outcome at 3 months on day 1 post-injury samples based on the NMR dataset. The lower plot displays the same metabolites using dot and whisker plots showing the specific concentrations of each of the metabolites. Y-axis shows concentration in µM. (W) p-value is calculated by the Wilcoxon Mann Whitney test, the rest of the p-value are calculated with t-test.


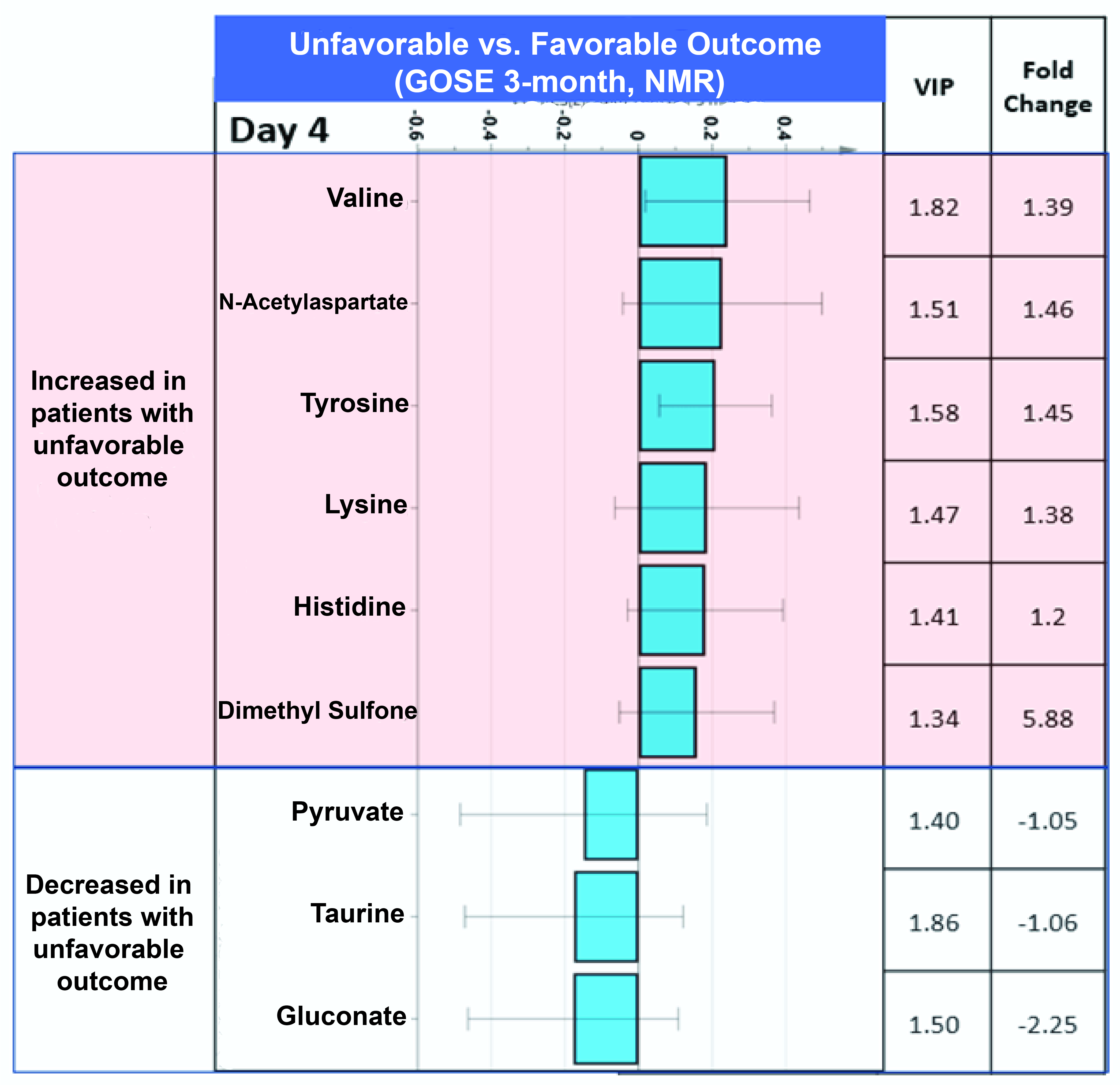


**Figure S8A.** The coefficient plot illustrates the relative concentration correlation of the metabolite alterations between the two cohorts with unfavorable and favorable GOSE outcome at 3 months on day 4 post-injury samples based on the ^1^H-NMR dataset. VIP score and fold change are also displayed for each metabolite. Only metabolites with a VIP score of > 1 regardless of sign are shown.

| GOSE 3-month (Day 4) NMR | | | | | |
| --- | --- | --- | --- | --- | --- |
| **Name** | **Mean (SD) of Favorable (**µM) | **Mean (SD) of Unfavorable (**µM) | **p-value** | **Fold Change** | **Unfavorable / Favorable** |
| Lactate | 19.621 (5.153) | 26.251 (3.743) | 0.0006 | -1.34 | Up |
| Valine | 4.350 (1.500) | 6.056 (1.080) | 0.0018 | -1.39 | Up |
| N-Acetylaspartate | 0.517 (0.211) | 0.754 (0.204) | 0.0093 | -1.46 | Up |
| Arginine | 1.967 (0.602) | 3.101 (1.176) | 0.0152 | -1.58 | Up |
| Lysine | 3.325 (1.027) | 4.583 (1.419) | 0.03 | -1.38 | Up |
| 2-Aminobutyrate | 1.283 (0.477) | 1.774 (0.554) | 0.035 | -1.38 | Up |
| Choline | 0.143 (0.048) | 0.195 (0.062) | 0.0406 | -1.37 | Up |
| Adipate | 0.133 (0.029) | 0.168 (0.065) | 0.0497 | -1.27 | Up |
| Tyrosine | 2.147 (0.789) | 3.113 (0.688) | 0.0030 (W) | -1.45 | Up |
| Gluconate | 2.038 (1.507) | 0.906 (0.585) | 0.0099 (W) | 2.25 | Down |
| Histidine | 1.161 (0.389) | 1.388 (0.235) | 0.0134 (W) | -1.2 | Up |
| Glutamate | 1.409 (0.501) | 2.475 (1.468) | 0.0237 (W) | -1.76 | Up |
| Urea | 0.434 (0.213) | 1.009 (0.864) | 0.0237 (W) | -2.33 | Up |
| Isoleucine | 1.637 (0.562) | 2.085 (0.602) | 0.0270 (W) | -1.27 | Up |
| Alanine | 3.529 (1.538) | 4.811 (1.147) | 0.0349 (W) | -1.36 | Up |
| Leucine | 2.788 (0.816) | 3.610 (1.135) | 0.0446 (W) | -1.29 | Up |


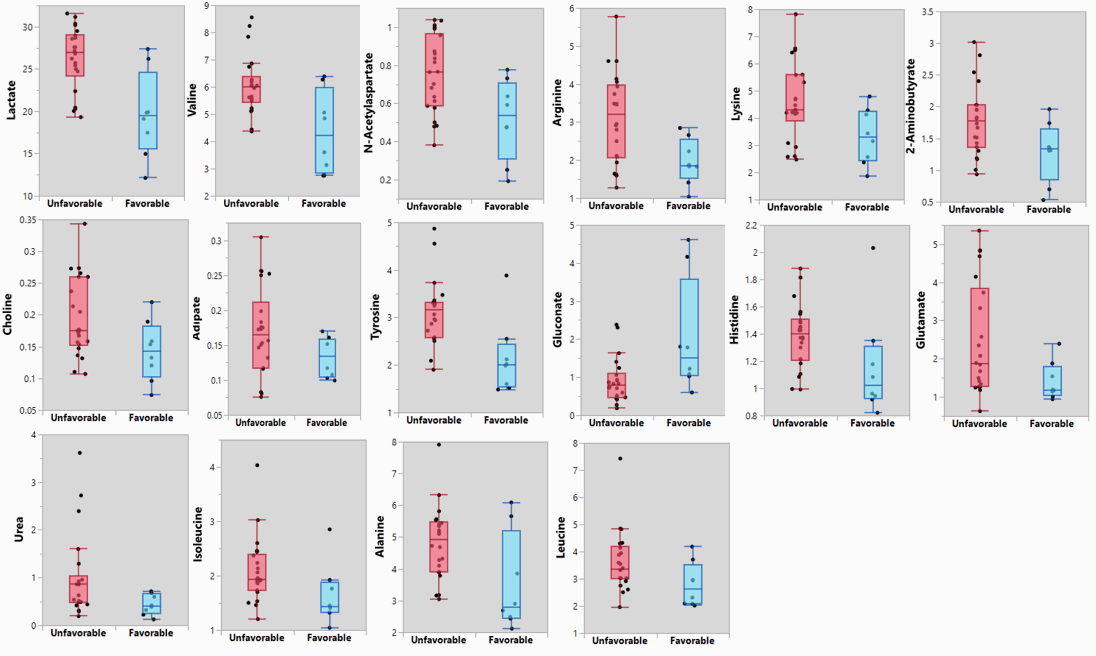


**Fig S8B**. Univariate analysis (t-test analysis) shows the significant (p< 0.05) metabolites between the two cohorts with unfavorable and favorable GOSE outcome at 3 months on day 4 post-injury samples based on the NMR dataset. The lower plot displays the same metabolites using dot and whisker plots showing the specific concentrations of each of the metabolites. Y-axis shows concentration in µM. (W) p-value is calculated by the Wilcoxon Mann Whitney test, the rest of the p-value are calculated with t-test.


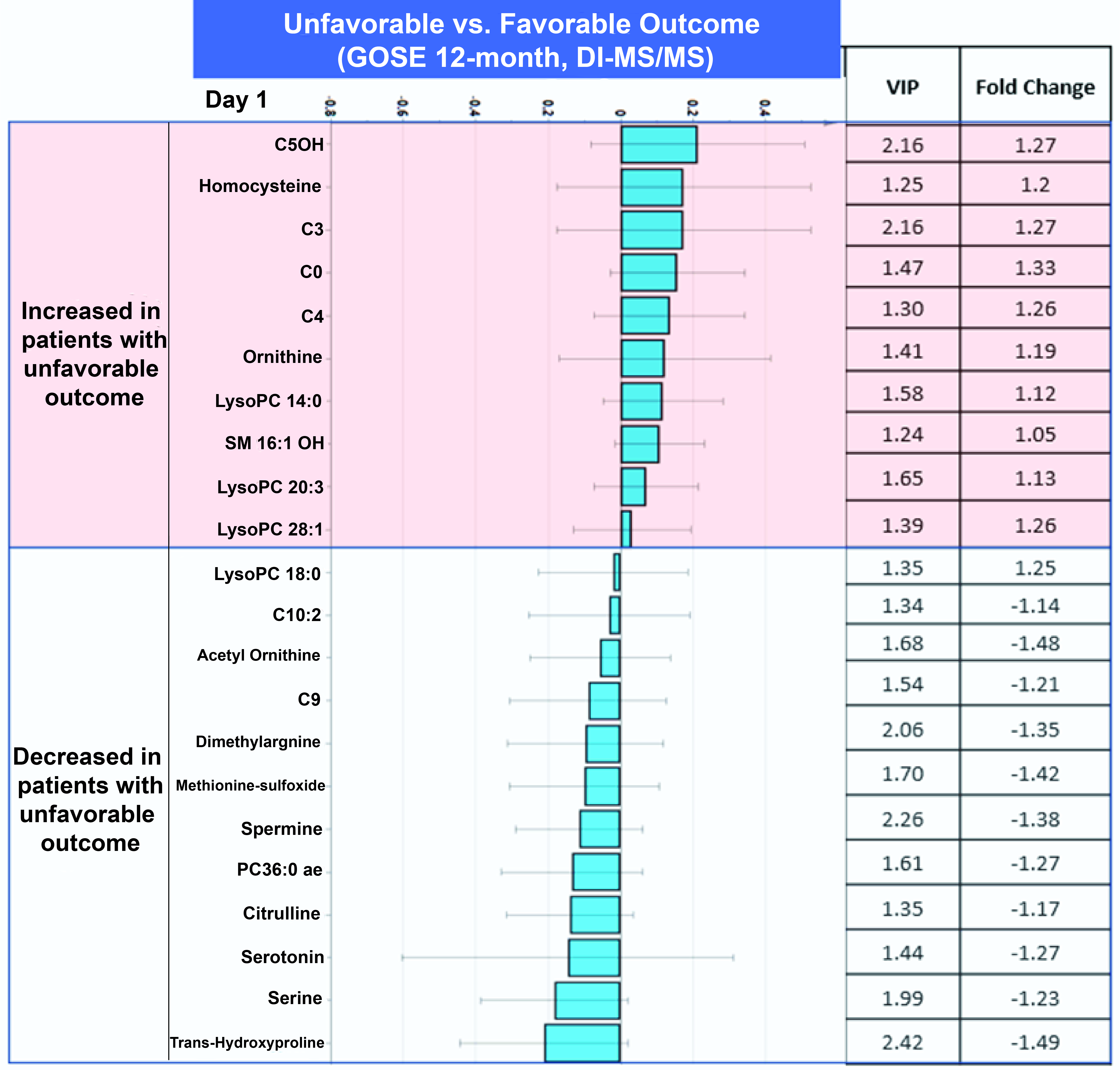


**Figure S9A.** The coefficient plot illustrates relative concentration correlation of the metabolite alterations between the two cohorts with unfavorable and favorable GOSE outcome at 12 months on day 1 post-injury samples based on the DI/LC-MS/MS dataset. VIP score and fold change are also displayed for each metabolite.

| GOSE 12-month (Day 1) DIMS-MS | | | | | |
| --- | --- | --- | --- | --- | --- |
| **Name** | **Mean (SD) of Favorable (**µM) | **Mean (SD) of Unfavorable (**µM) | **p-value** | **Fold Change** | **Unfavorable/**  **Favorable** |
| Trans-Hydroxyproline | 5.250 (1.661) | 3.521 (0.957) | 0.0018 | -1.49 | Down |
| Methionine-sulfoxide | 0.814 (0.328) | 0.573 (0.193) | 0.0221 | -1.42 | Down |
| Acetyl-ornithine | 0.568 (0.269) | 0.383 (0.160) | 0.0315 | -1.48 | Down |
| Dimethylarginine A | 0.394 (0.140) | 0.292 (0.102) | 0.0318 | -1.35 | Down |
| Serine | 74.190 (17.235) | 60.344 (16.826) | 0.0374 | -1.23 | Down |
| Spermine | 0.203 (0.082) | 0.147 (0.030) | 0.0027 (W) | -1.38 | Down |
| PC360AE | 0.897 (0.263) | 0.708 (0.193) | 0.0470 (W) | -1.27 | Down |


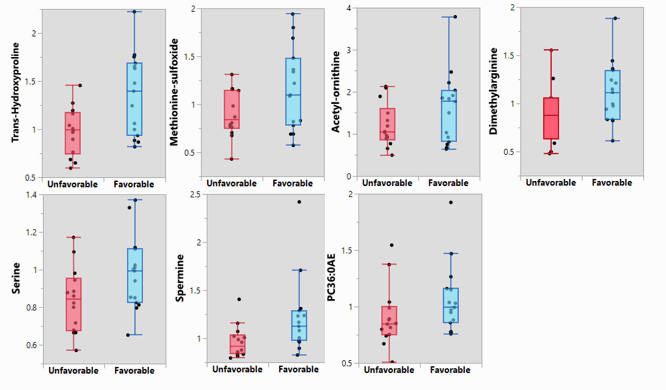


**Fig S9B**. Univariate analysis (t-test analysis) shows the significant (p< 0.05) metabolite between the two cohorts with unfavorable and favorable GOSE outcome at 12 months on day 1 post-injury samples based on the DIMS/MS dataset. The lower plot displays the same metabolites using dot and whisker plots showing the specific concentrations of each of the metabolites. Y-axis shows concentration in µM. (W) p-value is calculated by the Wilcoxon Mann Whitney test, the rest of the p-value are calculated with t-test.


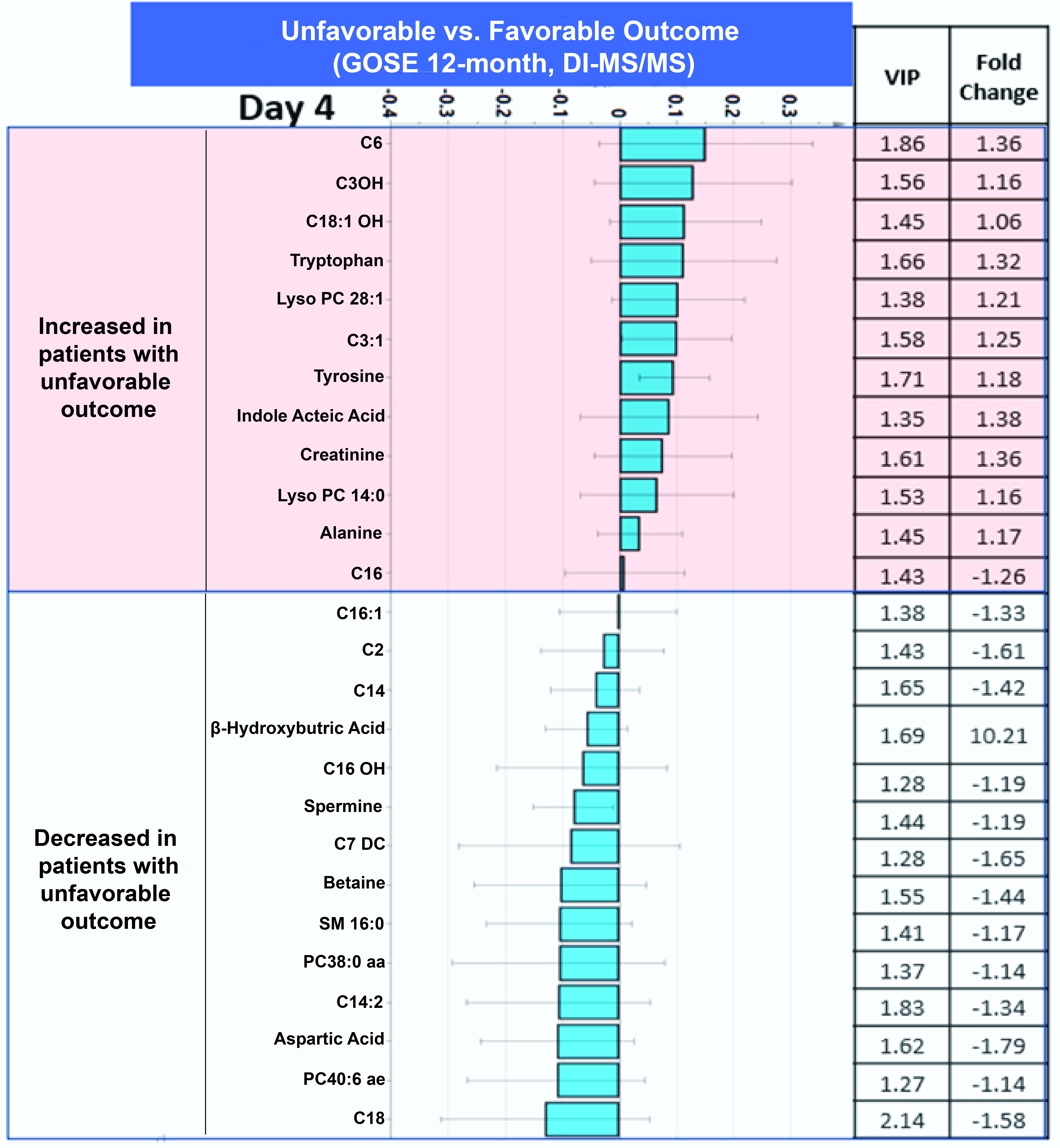


**Figure S10A.** The coefficient plot illustrates relative concentration correlation of the metabolite alterations between the two cohorts unfavorable and favorable GOSE outcome at 12 months on day 4 post-injury samples based on the DI/LC-MS/MS dataset. VIP score and fold change are also displayed for each metabolite.

| **GOSE 12-month (Day 4) DIMS-MS** | | | | | |
| --- | --- | --- | --- | --- | --- |
| **Name** | **Mean (SD) of Favorable (**µM) | **Mean (SD) of Unfavorable (**µM) | **p-value** | **Fold Change** | **Unfavorable/**  **Favorable** |
| Tyrosine | 39.250 (7.928) | 46.480 (8.290) | 0.0362 | 1.18 | Up |
| Creatinine | 126.029 (56.501) | 171.078 (47.437) | 0.041 | 1.36 | Up |
| Betaine | 36.109 (14.187) | 25.021 (8.153) | 0.0238 | -1.44 | Down |
| C18 | 0.039 (0.017) | 0.024 (0.009) | 0.0055 (W) | -1.58 | Down |
| C142 | 0.039 (0.007) | 0.029 (0.017) | 0.0066 (W) | -1.34 | Down |
| Aspartic acid | 12.717 (7.980) | 7.108 (3.371) | 0.0457 (W) | -1.79 | Down |


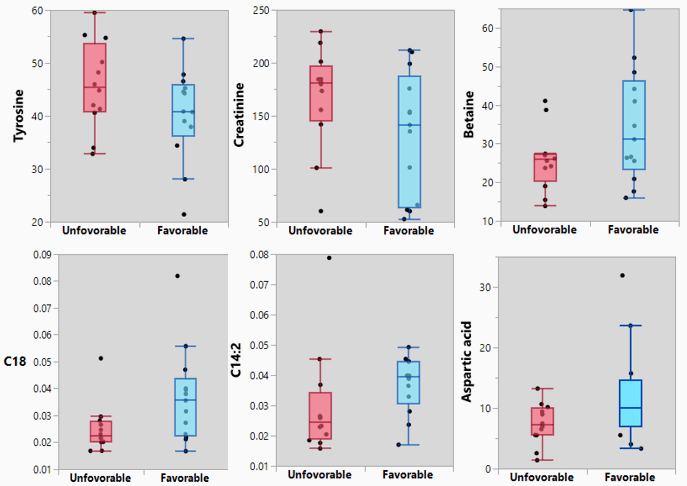


**Fig S10B**. Univariate analysis (t-test analysis) shows the significant (p< 0.05) metabolite between the two cohorts with unfavorable and favorable GOSE outcome at 12 months on day 4 post-injury samples based on the DIMS/MS dataset. The lower plot displays the same metabolites using dot and whisker plots showing the specific concentrations of each of the metabolites. Y-axis shows concentration in µM. (W) p-value is calculated by the Wilcoxon Mann Whitney test, the rest of the p-value are calculated with t-test.

------------------------------------------------------------------------------------------------------------------------------------


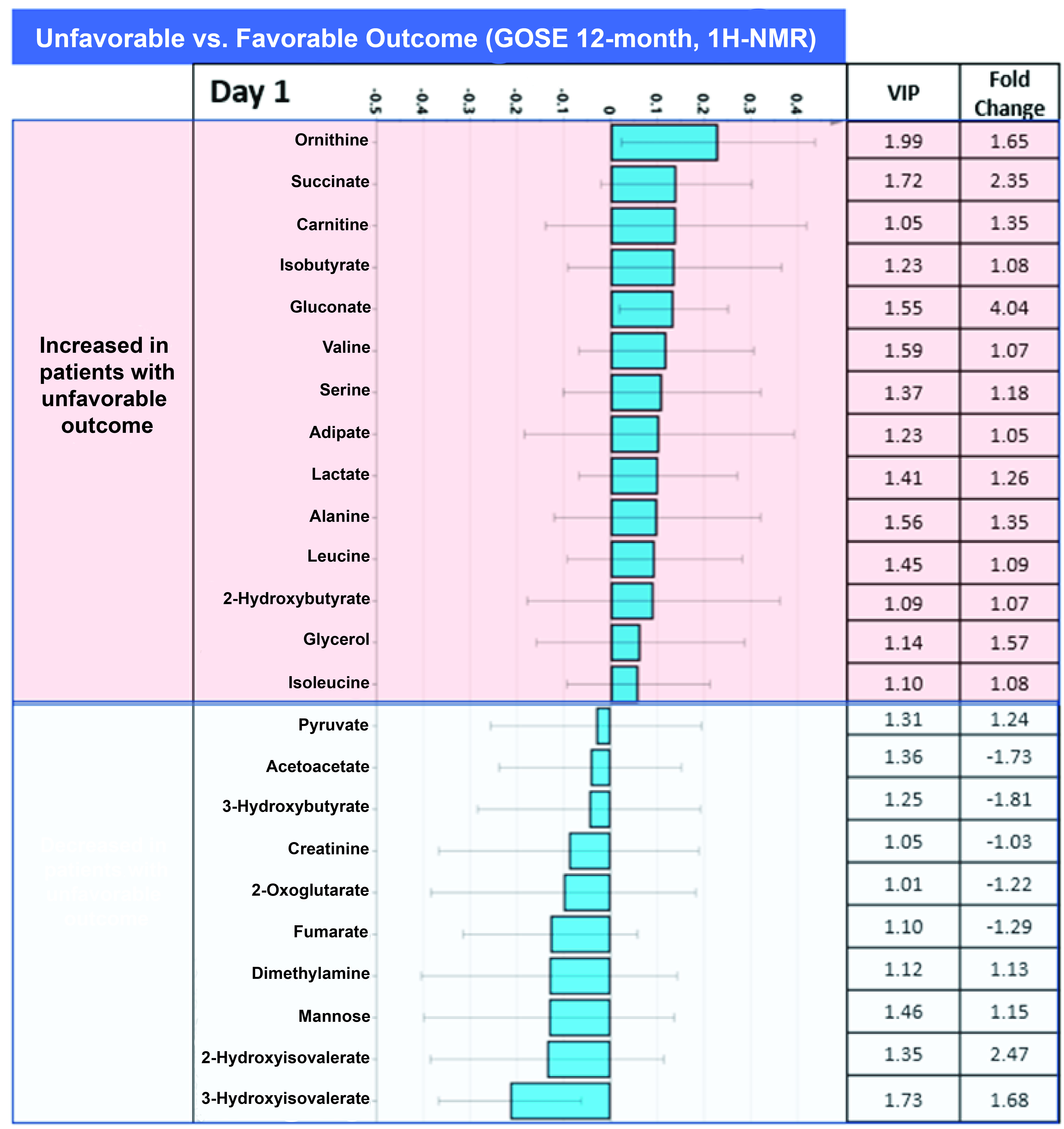


**Figure S11A.** The coefficient plot illustrates relative concentration correlation of the metabolite alterations between the two cohorts with unfavorable and favorable GOSE outcome at 12 months on day 1 post-injury samples based on the ^1^H-NMR dataset. VIP score and fold change are also displayed for each metabolite.

| **GOSE 12-month (Day 1) NMR** | | | | | |
| --- | --- | --- | --- | --- | --- |
| **Name** | **Mean (SD) of Favorable (**µM) | **Mean (SD) of Unfavorable (**µM) | **p-value** | **Fold Change** | **Unfavorable/**  **Favorable** |
| Alanine | 4.450 (1.081) | 5.999 (2.521) | 0.0483 | -1.35 | Up |
| 3-Hydroxyisovalerate | 0.048 (0.026) | 0.029 (0.012) | 0.0328 (W) | -1.68 | Down |
| Ornithine | 0.719 (0.276) | 1.183 (0.799) | 0.0367 (W) | 1.65 | UP |


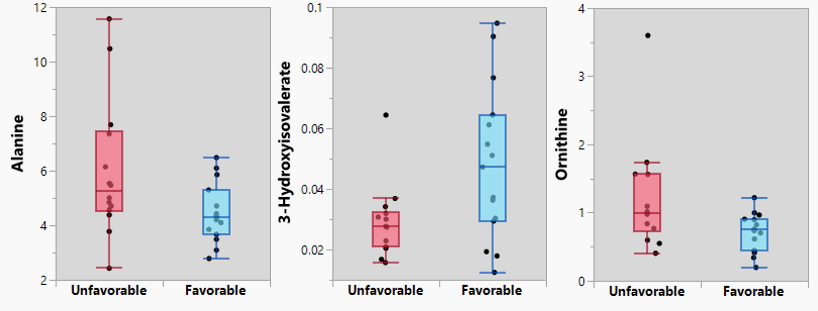


**Fig S11B**. Univariate analysis (t-test analysis) shows the significant (p< 0.05) metabolites between the two cohorts with unfavorable and favorable GOSE outcome at 12 months on day 1 post-injury samples based on the NMR dataset. The lower plot displays the same metabolites using dot and whisker plots showing the specific concentrations of each of the metabolites. Y-axis shows concentration in µM. (W) p-value is calculated by the Wilcoxon Mann Whitney test, the rest of the p-value are calculated with t-test.


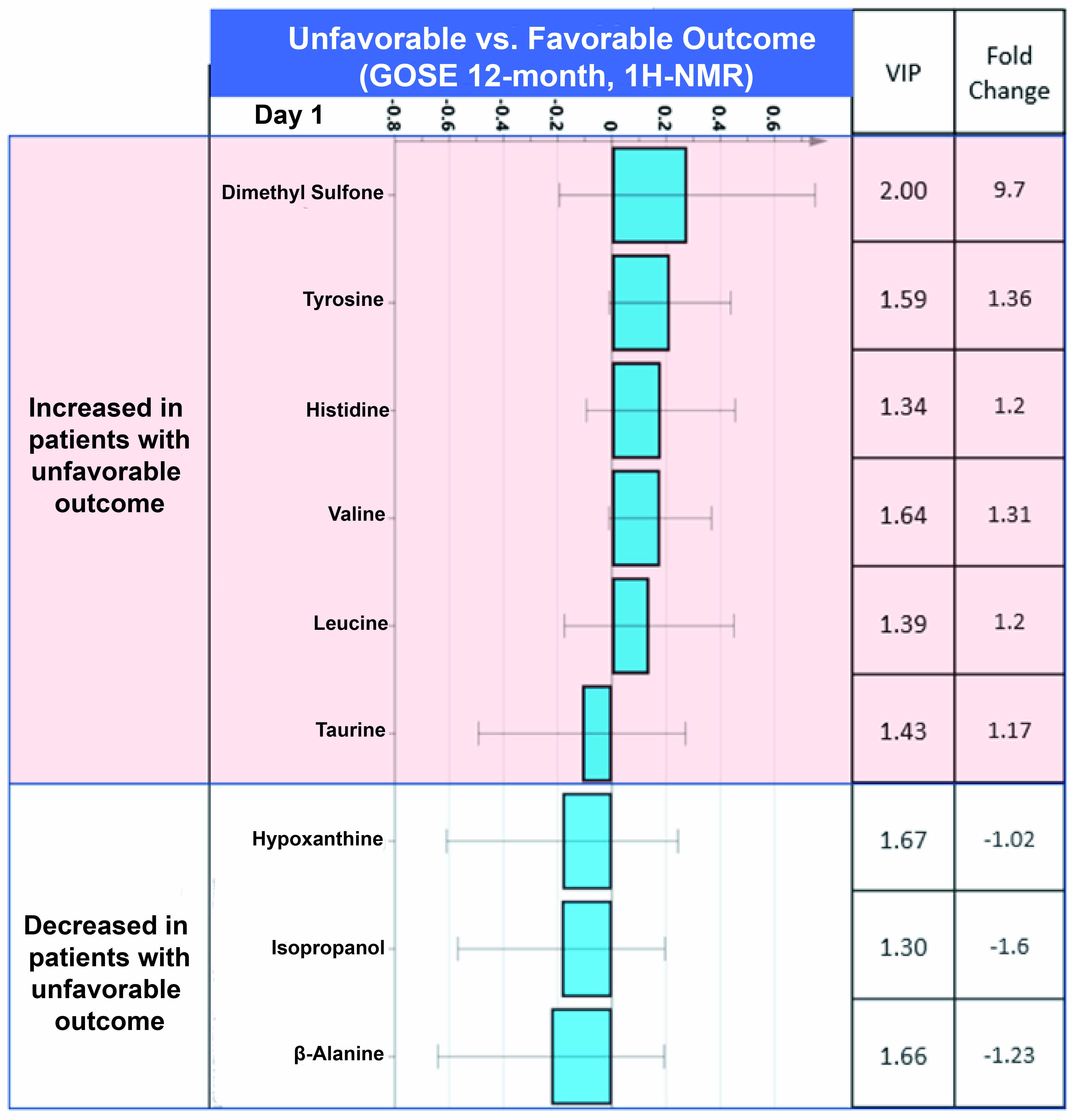


**Figure S12A.** The coefficient plot illustrates relative concentration correlation of the metabolite alterations between the two cohorts with unfavorable and favorable GOSE outcome at 12 months on day 4 post-injury samples based on the ^1^H-NMR dataset. VIP score and fold change are also displayed for each metabolite.

| GOSE 12-month (Day 4) NMR | | | | | |
| --- | --- | --- | --- | --- | --- |
| **Name** | **Mean (SD) of Favorable (**µM) | **Mean (SD) of Unfavorable (**µM) | **p-value** | **Fold Change** | **Unfavorable / Favorable** |
| Tyrosine | 2.299 (0.680) | 3.137 (0.850) | 0.0156 | 1.36 | Up |
| Valine | 4.464 (1.181) | 5.851 (1.445) | 0.0193 | 1.31 | Up |
| Alanine | 3.903 (1.398) | 5.320 (1.661) | 0.0374 | 1.36 | Up |
| Ornithine | 1.149 (0.495) | 1.588 (0.478) | 0.0443 | 1.38 | Up |
| Dimethyl sulfone | 0.059 (0.026) | 0.571 (1.041) | 0.0178 (W) | 9.7 | Up |


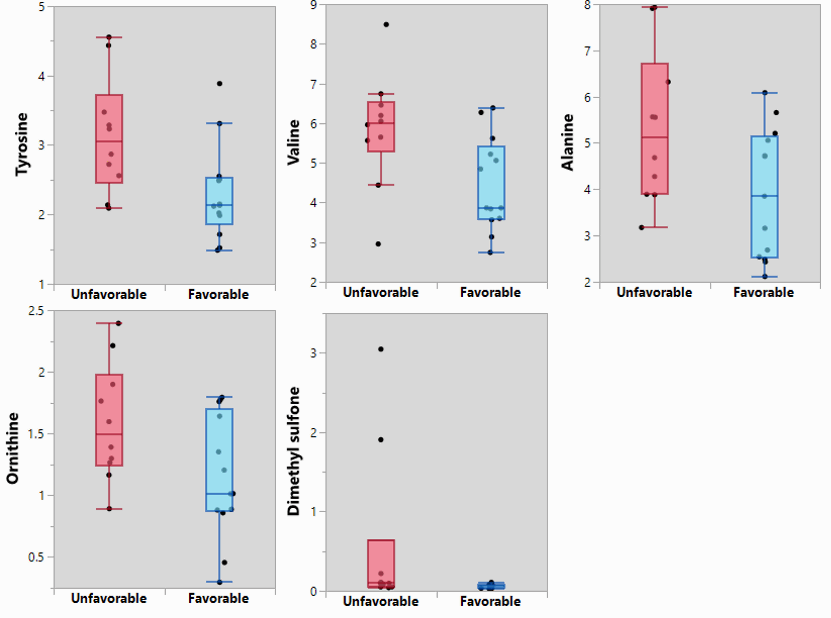


**Fig S12B**. Univariate analysis (t-test analysis) shows the significant (p< 0.05) metabolite between the two cohorts with unfavorable and favorable GOSE outcome at 12 months on day 4 post-injury samples based on the NMR dataset. The lower plot displays the same metabolites using dot and whisker plots showing the specific concentrations of each of the metabolites. Y-axis shows concentration in µM. (W) p-value is calculated by the Wilcoxon Mann Whitney test, the rest of the p-value are calculated with t-test.


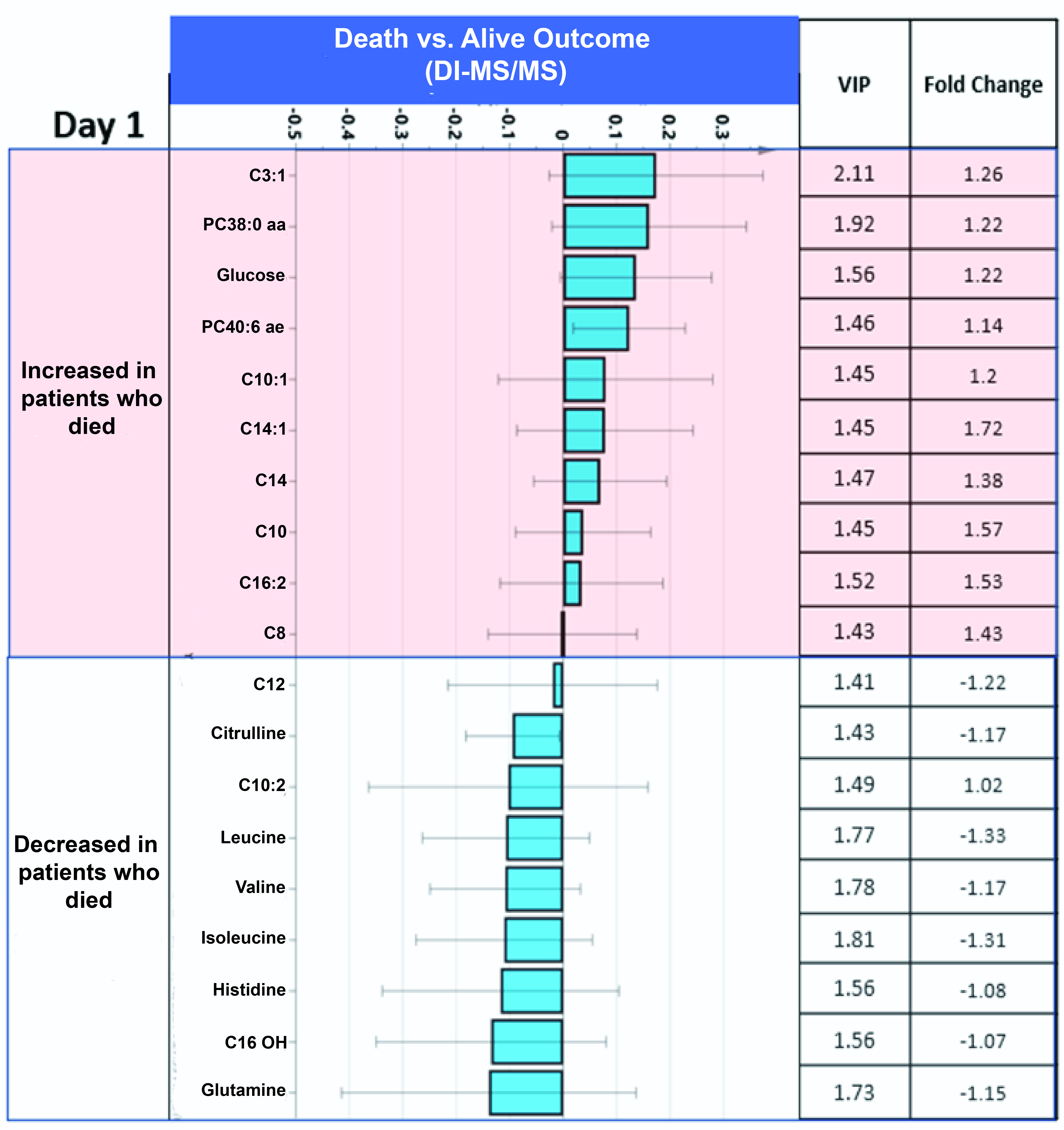


**Figure S13A.** The coefficient plot illustrates relative concentration correlation of the metabolite alterations between non-survivor and survivor cohorts at 3 months on day 1 post-injury based on the DI/LC-MS/MS data. VIP score and fold change are also displayed for each metabolite.

| **Mortality outcome (Day 1) DIMS-MS** | | | | | |
| --- | --- | --- | --- | --- | --- |
| **Name** | **Mean (SD) of Alive (**µM) | **Mean (SD) of Died (**µM) | **p-value** | **Fold Change** | **Alive/Died** |
| Isoleucine | 59.398 (23.850) | 41.511 (16.616) | 0.0066 | 1.43 | Up |
| Glutamine | 426.519 (101.926) | 341.101 (96.682) | 0.0068 | 1.25 | Up |
| Histidine | 87.192 (22.533) | 74.265 (15.755) | 0.0345 | 1.17 | Up |
| C3:1 | 0.042 (0.013) | 0.050 (0.011) | 0.0532 | -1.17 | Down |
| Valine | 156.222 (50.515) | 121.700 (37.588) | 0.0177 (W) | 1.28 | Up |
| Leucine | 127.249 (60.615) | 89.486 (34.529) | 0.0261 (W) | 1.42 | Up |
| Citrulline | 20.039 (8.801) | 15.305 (7.297) | 0.0278 (W) | 1.31 | Up |
| PC38:0AA | 1.164 (0.351) | 1.290 (0.285) | 0.0364 (W) | -1.11 | Down |


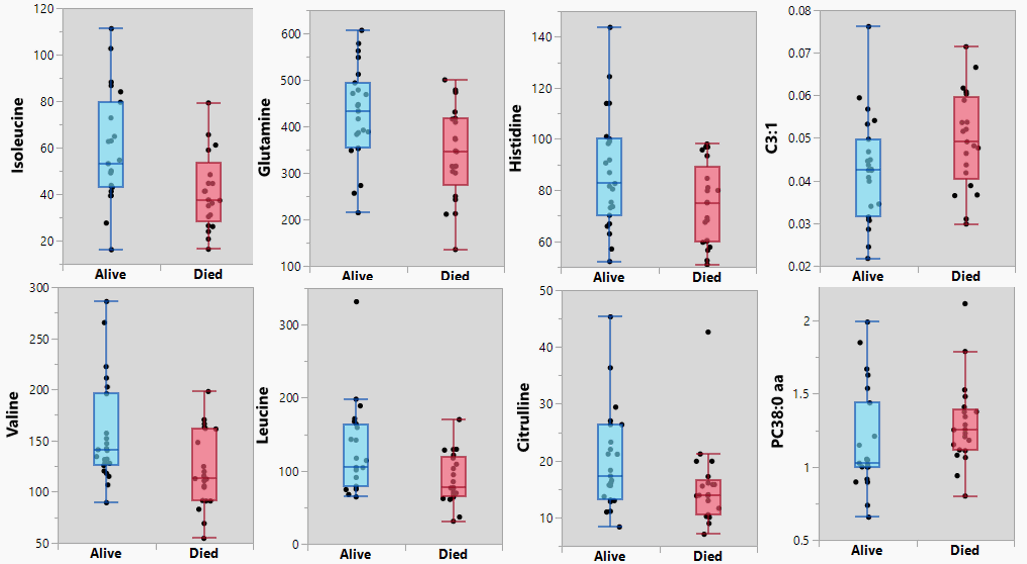


**Fig S13B**. Univariate analysis (t-test analysis) shows the significant (p< 0.05) metabolite between non-survivor and survivor cohorts at 3 months on day 1 post-injury based on the DI/LC-MS/MS data. The lower plot displays the same metabolites using dot and whisker plots showing the specific concentrations of each of the metabolites. Y-axis shows concentration in µM. (W) p-value is calculated by the Wilcoxon Mann Whitney test, the rest of the p-value are calculated with t-test.

-------------------------------------------------------------------------------------------------------------------------------------


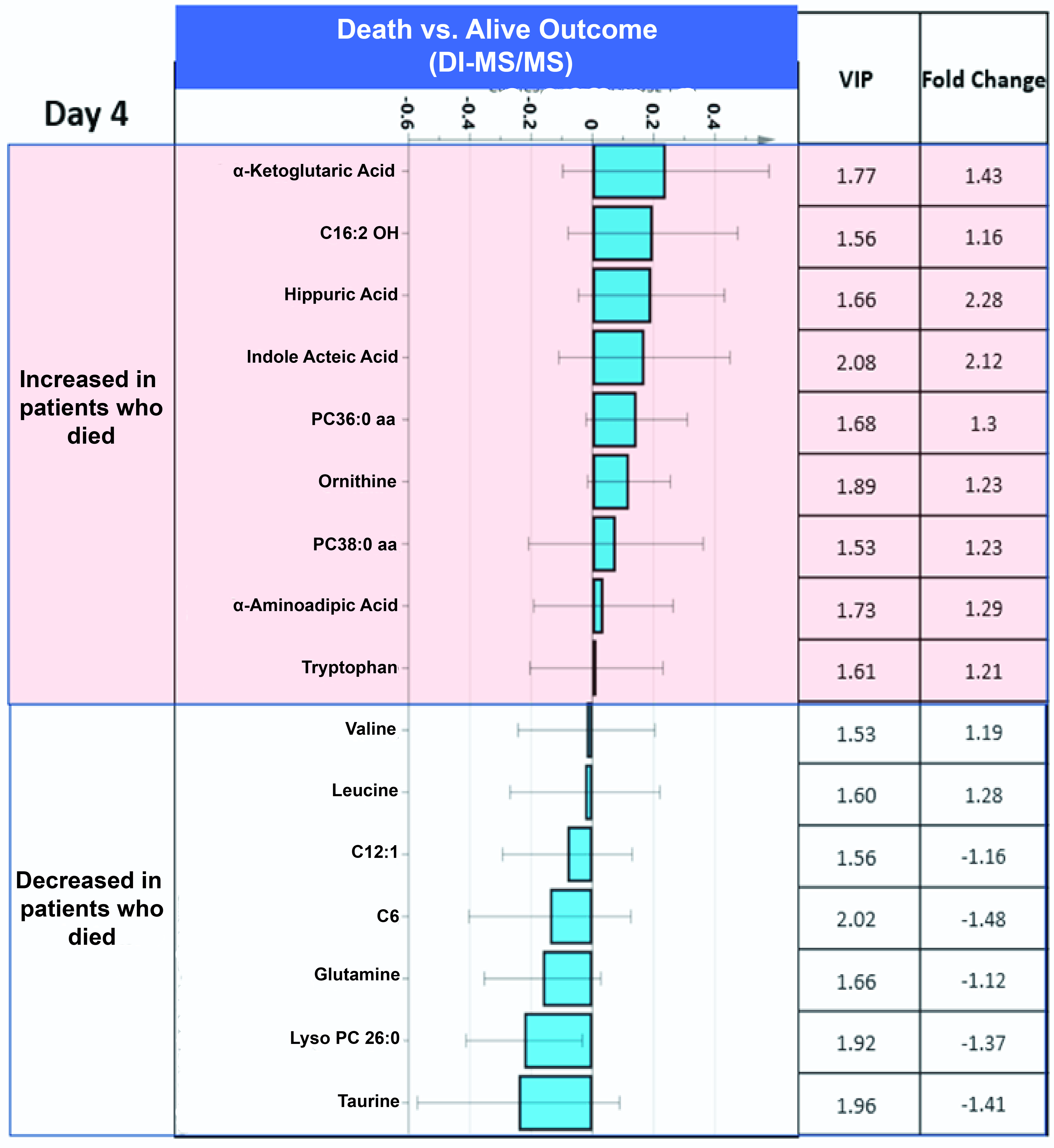


**Figure S14A.** The coefficient plot illustrates relative concentration correlation of the metabolite alterations between non-survivor and survivor cohorts at 3 months-based day 4 post injury DI/LC-MS/MS data. VIP score and fold change are also displayed for each metabolite.

| Mortality outcome (Day 4) DIMS-MS | | | | | |
| --- | --- | --- | --- | --- | --- |
| **Name** | **Mean (SD) of Alive (**µM) | **Mean (SD) of Died (**µM) | **p-value** | **Fold Change** | **Died/Alive** |
| Taurine | 50.190 (16.576) | 32.495 (15.675) | 0.0062 | 1.54 | Down |
| Glutamine | 410.051 (84.099) | 324.887 (74.218) | 0.0076 | 1.26 | Down |
| LYSOC26:0 | 0.511 (0.181) | 0.334 (0.152) | 0.0088 | 1.53 | Down |
| C6 | 0.086 (0.046) | 0.052 (0.016) | 0.0009 (W) | 1.66 | Down |
| C12:1 | 0.223 (0.070) | 0.171 (0.058) | 0.0227 (W) | 1.31 | Down |
| Creatinine | 141.578 (62.375) | 97.911 (65.780) | 0.0317 (W) | 1.45 | Down |
| C14:1 | 0.086 (0.032) | 0.071 (0.052) | 0.0353 (W) | 1.21 | Down |
| C8 | 0.239 (0.224) | 0.141 (0.039) | 0.0392 (W) | 1.7 | Down |
| Glycine | 176.007 (54.428) | 141.751 (46.064) | 0.0435 (W) | 1.24 | Down |
| C10 | 0.169 (0.126) | 0.114 (0.036) | 0.0435 (W) | 1.48 | Down |


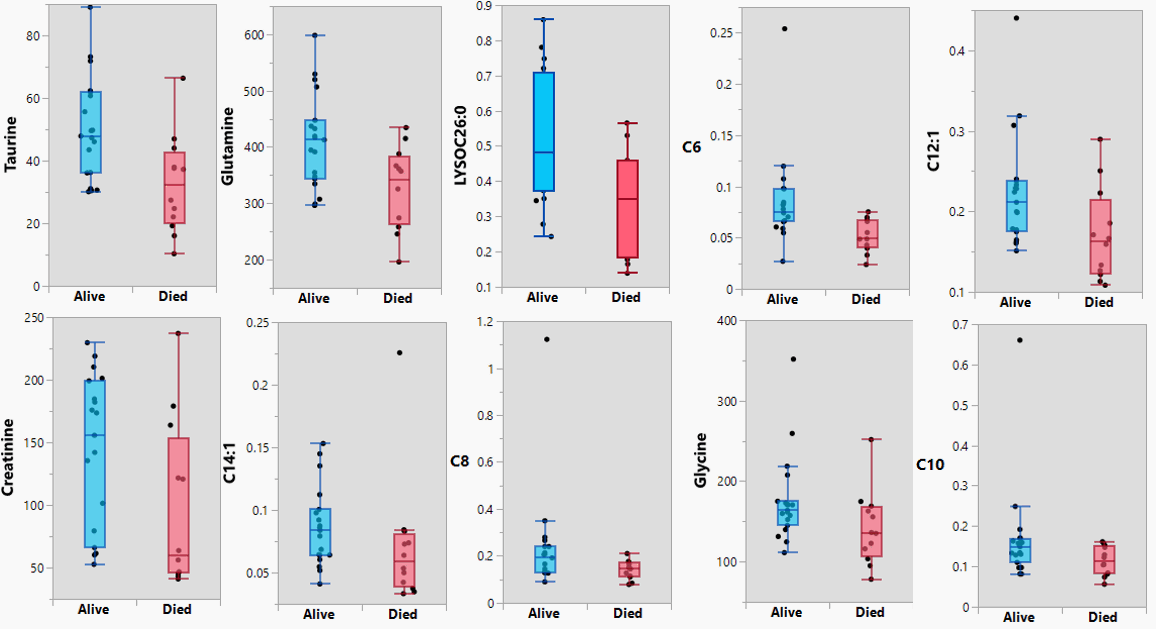


**Fig S14B**. Univariate analysis (t-test analysis) shows the significant (p< 0.05) metabolite between non-survivor and survivor cohorts at 3 months on day 4 post-injury based on the DI/LC-MS/MS data. The lower plot displays the same metabolites using dot and whisker plots showing the specific concentrations of each of the metabolites. Y-axis shows concentration in µM. (W) p-value is calculated by the Wilcoxon Mann Whitney test, the rest of the p-value are calculated with t-test.

-------------------------------------------------------------------------------------------------------------------------------------


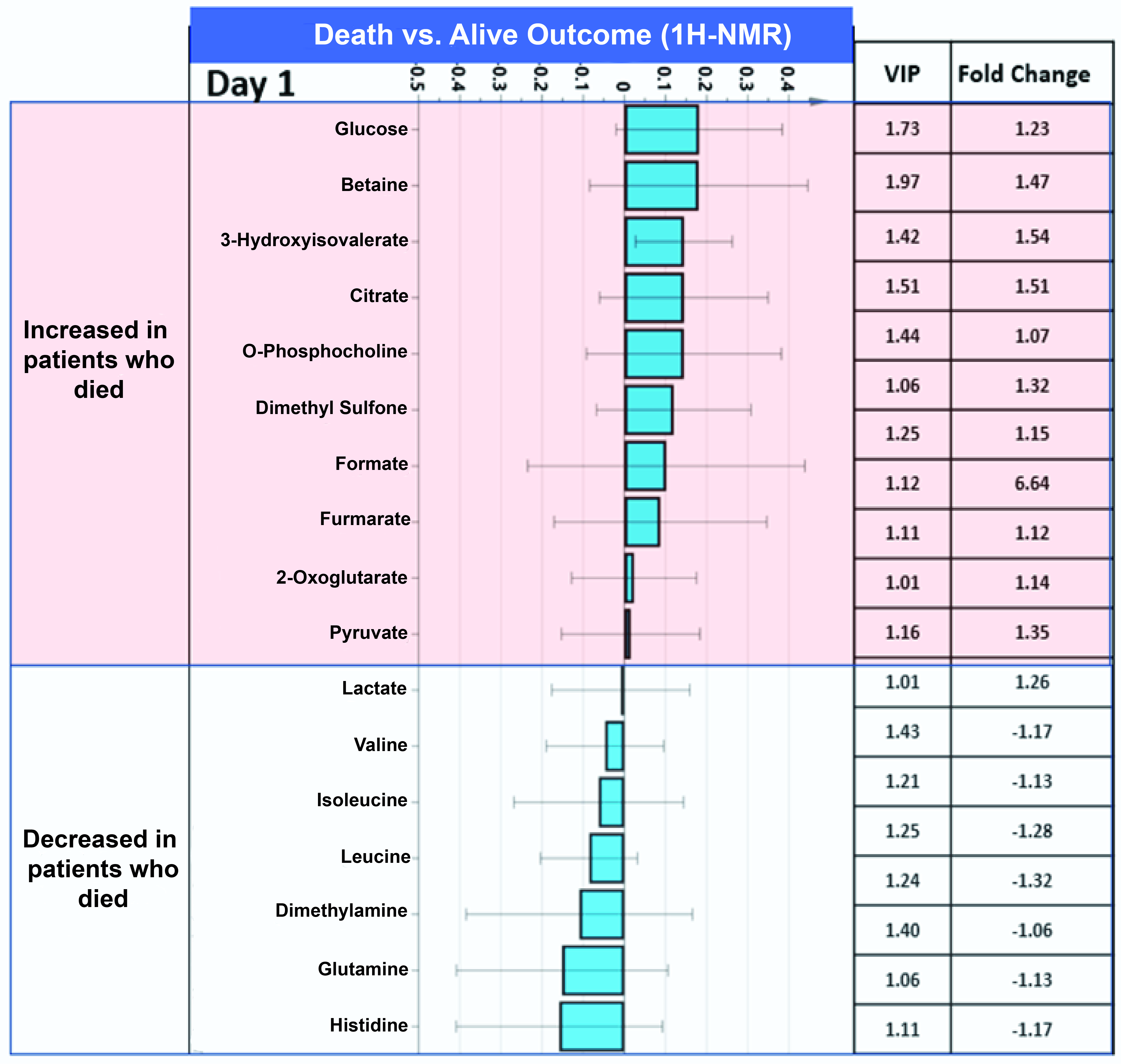


**Figure S15A.** The coefficient plot illustrates relative concentration correlation of the metabolite alterations between non-survivor and survivor cohorts at 3 months-based day 1 post injury ^1^H-NMR data. VIP score and fold change are also displayed for each metabolite.

| **Mortality outcome (Day 1) NMR** | | | | | |
| --- | --- | --- | --- | --- | --- |
| **Name** | **Mean (SD) of Alive (**µM) | **Mean (SD) of Died (**µM) | **p-value** | **Fold Change** | **Died/Alive** |
| Glucose | 261.897 (75.982) | 322.188 (92.781) | 0.0226 | -1.23 | Up |
| Fumarate | 0.032 (0.014) | 0.044 (0.018) | 0.0255 | -1.35 | Up |
| Isoleucine | 1.303 (0.538) | 0.991 (0.358) | 0.0177 (W) | 1.32 | Down |
| Betaine | 0.896 (0.309) | 1.315 (0.733) | 0.0230 (W) | -1.47 | Up |
| Leucine | 2.468 (0.983) | 1.927 (0.753) | 0.0401 (W) | 1.28 | Down |
| Citrate | 4.176 (1.657) | 5.500 (2.384) | 0.0477 (W) | -1.32 | Up |


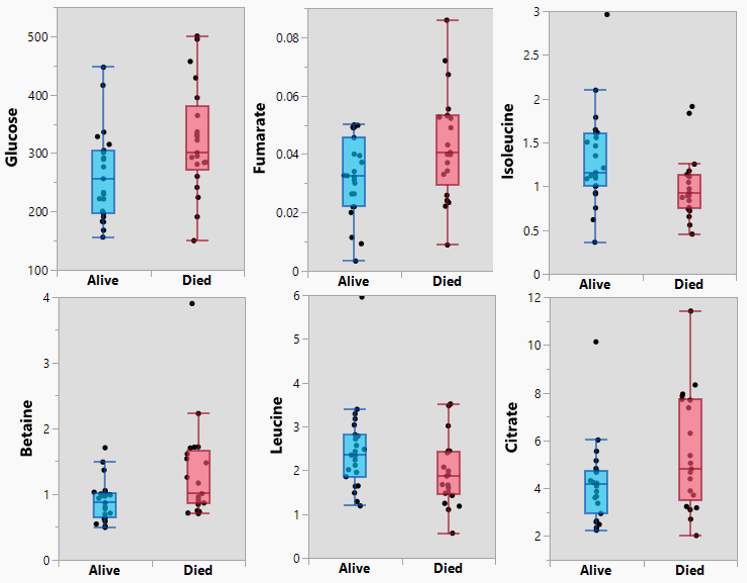


**Fig S15B**. Univariate analysis (t-test analysis) shows the significant (p< 0.05) metabolite between non-survivor and survivor cohorts at 3 months on day 1 post-injury based on the NMR data. The lower plot displays the same metabolites using dot and whisker plots showing the specific concentrations of each of the metabolites. Y-axis shows concentration in µM. (W) p-value is calculated by the Wilcoxon Mann Whitney test, the rest of the p-value are calculated with t-test.


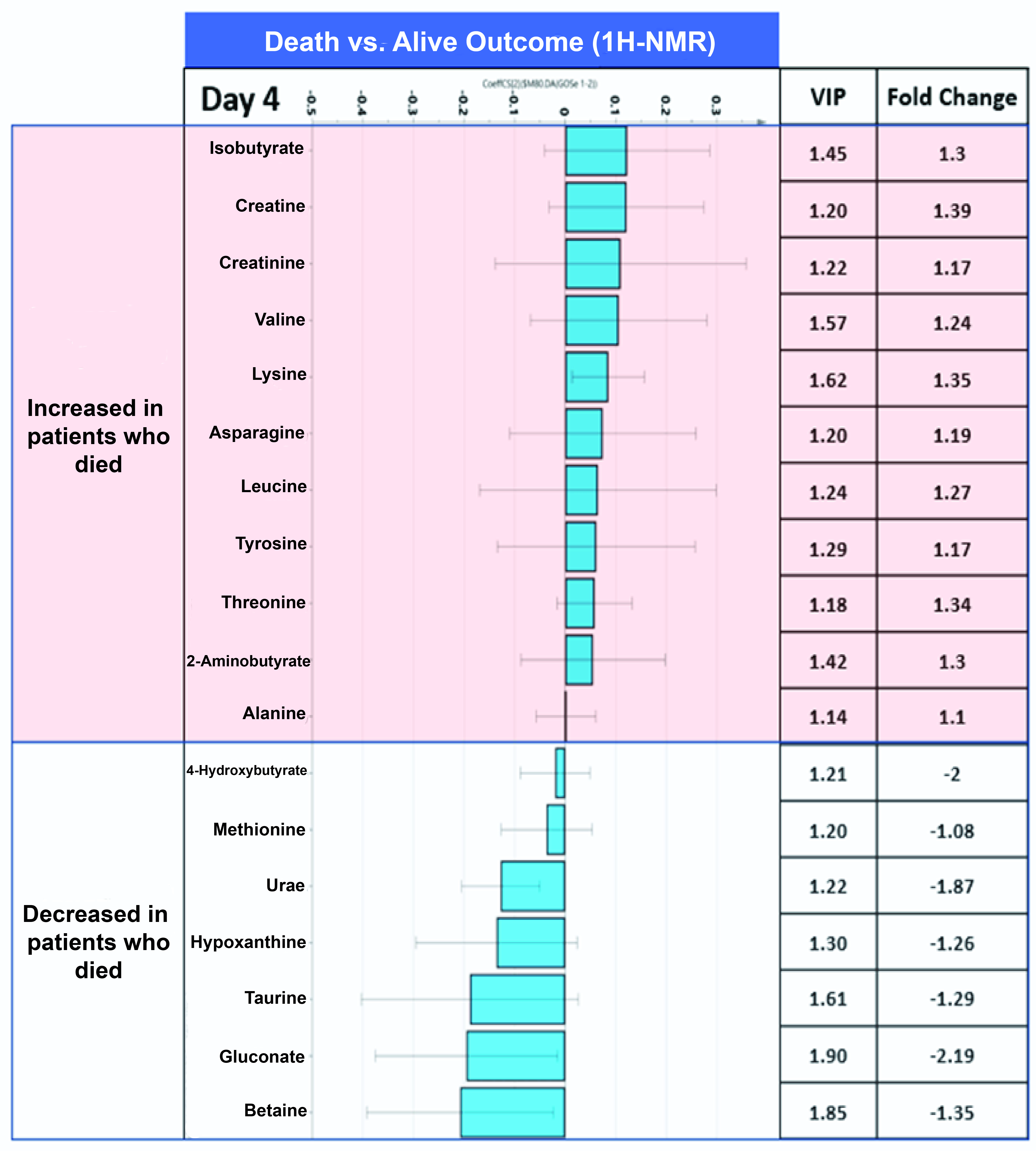


**Figure S16A.** The coefficient plot illustrates relative concentration correlation of the metabolite alterations between non-survivor and survivor cohorts at 3 months-based day 4 post injury ^1^H-NMR data. VIP score and fold change are also displayed for each metabolite. Only metabolites with a VIP score of > |1| regardless of sign are shown.

| **Mortality outcome (Day 4) NMR** | | | | | |
| --- | --- | --- | --- | --- | --- |
| **Name** | **Mean (SD) of Alive (**µM) | **Mean (SD) of Died (**µM) | **p-value** | **Fold Change** | **Alive/Died** |
| Valine | 5.110 (1.184) | 6.349 (1.320) | 0.0121 | -1.24 | Up |
| Lysine | 3.668 (1.206) | 4.961 (1.535) | 0.0154 | -1.35 | Up |
| Isobutyrate | 0.183 (0.056) | 0.238 (0.068) | 0.0223 | -1.3 | Up |
| 2-Aminobutyrate | 1.478 (0.426) | 1.918 (0.632) | 0.0301 | -1.3 | Up |
| Gluconate | 1.458 (1.041) | 0.667 (0.376) | 0.0016 (W) | 2.19 | Down |
| Betaine | 1.125 (0.387) | 0.830 (0.359) | 0.0346 (W) | 1.35 | Down |
| 3-Hydroxyisovalerate | 0.096 (0.216) | 0.067 (0.029) | 0.0387 (W) | 1.44 | Down |


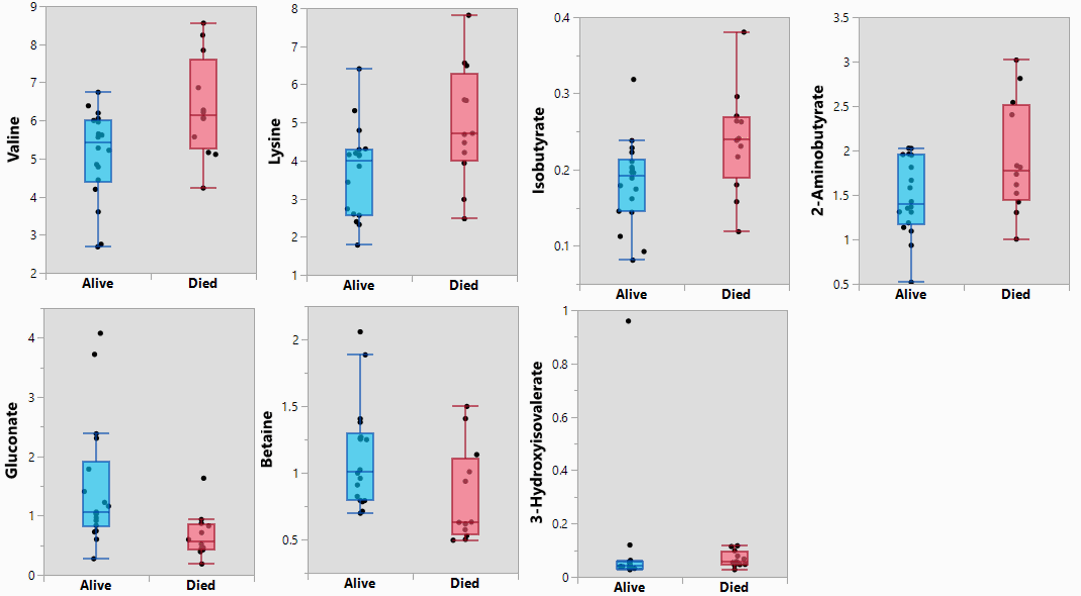


**Fig S16B**. Univariate analysis (t-test analysis) shows the significant (p< 0.05) metabolite between non-survivor and survivor cohorts at 3 months on day 4 post-injury based on the NMR data. The lower plot displays the same metabolites using dot and whisker plots showing the specific concentrations of each of the metabolites. Y-axis shows concentration in µM. (W) p-value is calculated by the Wilcoxon Mann Whitney test, the rest of the p-value are calculated with t-test.


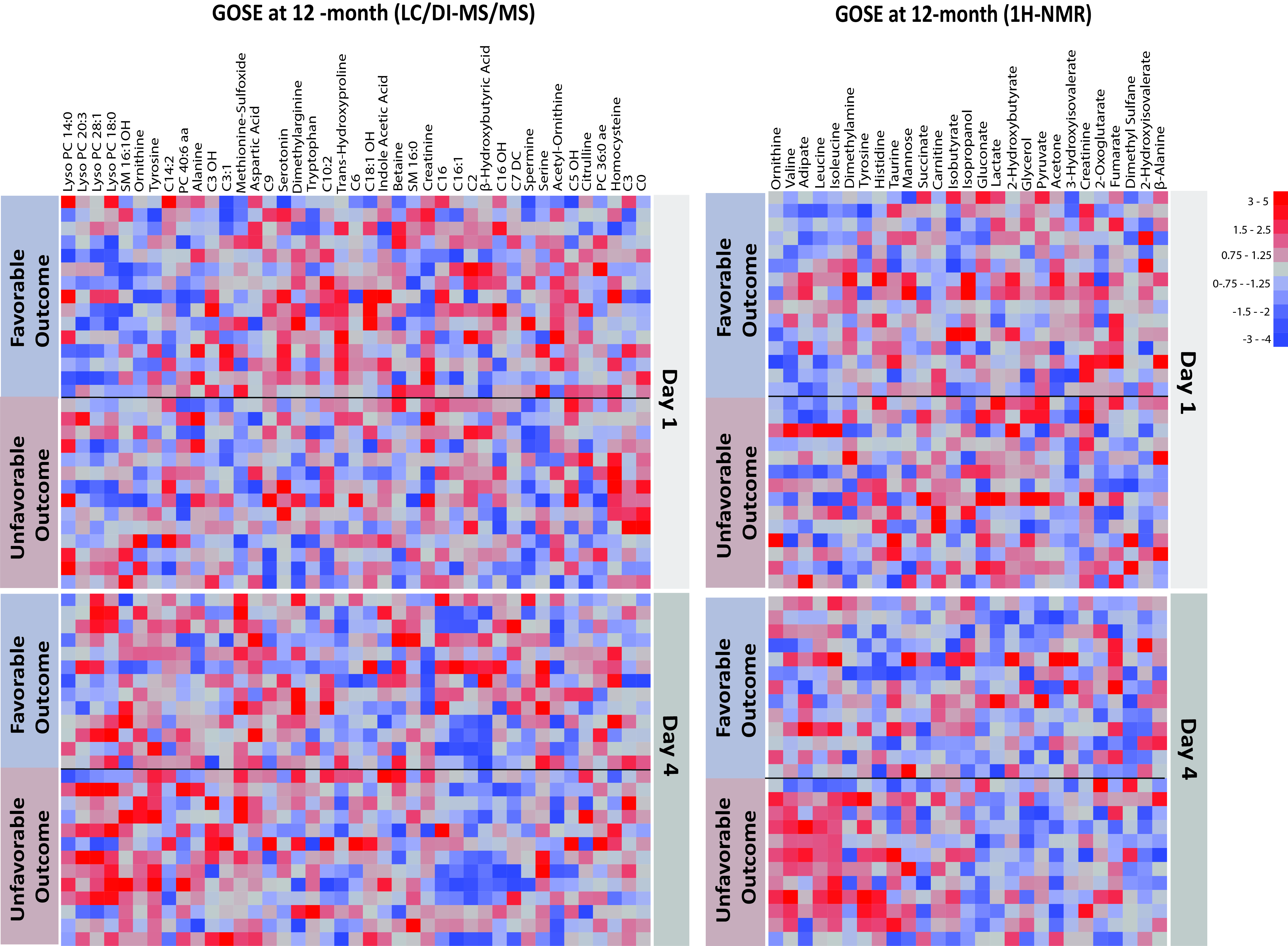


**Figure S17**. Heatmap metabolite plots show the metabolite alterations at day 1 and day 4 among the most differentiating metabolites to predict unfavorable and favorable outcomes at 12-month using DI/LC-MS/MS and ^1^H-NMR. The heatmap key shows the normalized and transformed concentration for each metabolite. Since each metabolite has its own concentration in the cell plots the key shows the range of concentration.


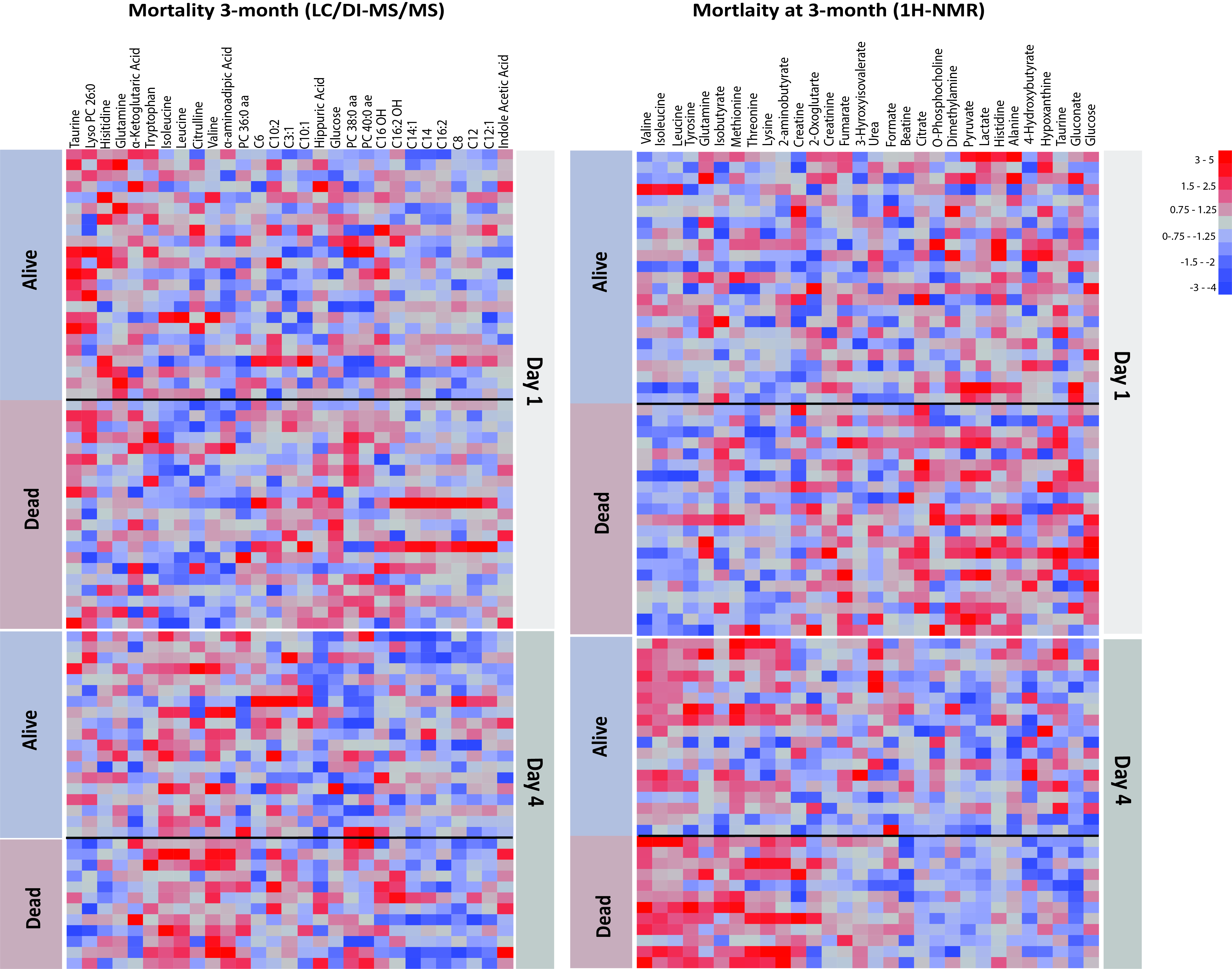


**Figure S18**. Heatmap metabolite plots show the metabolite alterations at day 1 and day 4 among the most differentiating metabolites to predict mortality outcomes at 3-month using DI/LC-MS/MS and ^1^H-NMR. The heatmap key shows the normalized and transformed concentration for each metabolite. Since each metabolite has its own concentration in the cell plots the key shows the range of concentration.

| **Analytical Platform** | **Sample time** | **GOSE outcome 3-month** | **Predicted Rate Training Set** | **Predicted Rate Validation Set** | **Training AUC** | **Validation**  **AUC** |
| --- | --- | --- | --- | --- | --- | --- |
| DI-MS/MS | Day 1 | Poor outcome | 1.0 | 0.917 | 1.0 | 0.97 |
|  |  | Good outcome | 1 | 0.66 |  |  |
|  | Day 4 | Poor outcome | 1.0 | 0.87 | 1.0 | 0.95 |
|  |  | Good outcome | 1.0 | 1.0 |  |  |
| ^1^H-NMR | Day 1 | Poor outcome | 1.0 | 1.0 | 0.95 | 0.97 |
|  |  | Good outcome | 0.83 | 0.66 |  |  |
|  | Day 4 | Poor outcome | 0.929 | 1.0 | 1.0 | 0.95 |
|  |  | Good outcome | 1.0 | 0.667 |  |  |

**Table S4.** ANN analysis indicates the prognosis of unfavorable outcome is more predictive than the prognosis of favorable GOSE outcome at 3 months. In addition, there is a higher predictability using day 4 data compared to day 1 data according to both training and validation sets.

| **Analytical Platform** | **Sample time** | **GOSE outcome 12-month** | **Predicted Rate Training Set** | **Predicted Rate Validation Set** | **Training AUC** | **Validation AUC** |
| --- | --- | --- | --- | --- | --- | --- |
| DI-MS/MS | Day 1 | Poor outcome | 0.778 | 0.8 | 0.97 | 0.96 |
|  |  | Good outcome | 1.0 | 1.0 |  |  |
|  | Day 4 | Poor outcome | 0.87 | 0.75 | 0.91 | 0.96 |
|  |  | Good outcome | 0.89 | 1.0 |  |  |
| ^1^H-NMR | Day 1 | Poor outcome | 1.0 | 0.6 | 1.0 | 1.0 |
|  |  | Good outcome | 1.0 | 1.0 |  |  |
|  | Day 4 | Poor outcome | 0.714 | 0.70 | 0.84 | 0.85 |
|  |  | Good outcome | 0.7 | 0.8 |  |  |

**Table S5.** ANN analysis indicates the prognosis of favorable GOSE outcome is more predictive than the prognosis of unfavorable outcome at 12 months as well as higher predictability of day 4 samples post sTBI compared to day 1 samples post sTBI.

| **Models** | **Q2** | **R2Y** | **Sensitivity** | **Specificity** | **AUC** | **Most Differentiating Clinical Variables (#)** | **Cohort's study** |
| --- | --- | --- | --- | --- | --- | --- | --- |
| Day 1 GOSE outcome at 3-month | 0.22 | 0.25 | 86 | 66 | 0.85 | Age, severity, Marshall Score, Hypoxemia | Poor outcome n= 35 Good outcome n=9 |
| Day 1 GOSE outcome at 12-month | 0.13 | 0.30 | 78 | 73 | 0.79 | Age, GCS, Hypoxemia, Loss of Consciousness | Poor outcome n= 14 Good outcome n= 15 |
| Day 4 GOSE outcome at 3-month | 0.26 | 0.40 | 75 | 82 | 0.82 | Age, Marshall score, Hypoxemia | Poor outcome n= 23 Good outcome n= 8 |
| Day 4 GOSE outcome at 12-month | 0.31 | 0.36 | 71 | 78 | 0.79 | Age, GCS, Gender, loss of conscious | Poor outcome n= 13 Good outcome n= 13 |
| Day 1 GOSE outcome (Mortality) | 0.37 | 0.44 | 66 | 78 | 0.87 | Age, severity | Died, n= 21 Alive, n= 23 |
| Day 4 GOSE outcome (Mortality) | 0.17 | 0.34 | 90 | 83 | 0.91 | Age, severity | Died, n= 12 Alive, n= 19 |

**Table S6.** The application of clinical variables for predicting GOSE outcome (unfavorable vs favorable) using SIMPLS at 3 months, 12 months and mortality (at 3 months). Note, there are a different number of patients (n, shown) in each analyzed cohort at different times; the prediction models are based on the GOSE outcome at 3 months, 12 months and mortality (at 3 months) as well as two sampling times; day 1 and day 4.

| **Models** | **Analytical Approach** | **Q2** | **R2Y** | **Sensitivity** | **Specificity** | **AUC** | **Most Differentiating Clinical Variables (#)** | **Cohort's study** |
| --- | --- | --- | --- | --- | --- | --- | --- | --- |
| DI-MS/MS  Day 1 GOSE outcome at 3-month | Metabolomics | 0.45 | 0.61 | 98 | 99 | 1 | 48 metabolites | Poor outcome n= 35  Good outcome n=9 |
|  | Metabolomics and clinical variables | 0.56 | 0.68 | 100 | 92 | 1 | 48 Metabolites + 4 Clinical Variables |  |
| DI-MS/MS  Day 4 GOSE outcome at 3-month | Metabolomics | 0.45 | 0.75 | 98 | 97 | 1 | 54 metabolites | Poor outcome n= 23  Good outcome n= 8 |
|  | Metabolomics and clinical variables | 0.56 | 0.80 | 100 | 92 | 0.98 | 54 Metabolites + 3 Clinical Variables |  |
| ^1^H-NMR  Day 1 GOSE outcome at 3-month | Metabolomics | 0.35 | 0.55 | 91 | 89 | 1 | 22 metabolites | Poor outcome n= 35  Good outcome n=9 |
|  | Metabolomics and clinical variables | 0.44 | 0.64 | 100 | 70 | 0.95 | 22 Metabolites + 4 Clinical variables |  |
| DI-MS/MS  Day 1 GOSE outcome (Mortality) | Metabolomics | 0.71 | 0.82 | 94 | 96 | 1 | 45 metabolites | Died, n= 21  Alive, n= 23 |
|  | Metabolomics and clinical variables | 0.82 | 0.86 | 93 | 100 | 0.99 | 45 Metabolites + 2 Clinical variables |  |
| ^1^H-NMR  Day 4 GOSE outcome (Mortality) | Metabolomics | 0.33 | 0.50 | 87 | 93 | 1 | 22 metabolites |  |
|  | Metabolomics and clinical variables | 0.47 | 0.60 | 81 | 95 | 0.91 | 20 Metabolites + 2 Clinical variables |  |

**Table S7.** SIMPLS analysis for the predicting GOSE outcome using the most differentiating metabolites (VIP > 1) (metabolomics) and a combination of the most differentiating metabolites (with VIP >1) and the most differentiating clinical variables (VIP > 1) shown in table **S6**. The table shows only prediction models where the combined use of clinical variables and metabolomics could improve the predictability compared to metabolomics only prediction models; notably the 12 month prediction models were not improved with the clinical variables therefore are not shown here.

| **Day 1 Samples (GOSE-3 month) LC/DIMS-MS** | | | | **Day 4 Samples (GOSE-3 month) LC/DIMS-MS** | | | |
| --- | --- | --- | --- | --- | --- | --- | --- |
| **Predictor** | **Contribution** | **Portion** | **Rank** | **Predictor** | **Contribution** | **Portion** | **Rank** |
| C14 | 1.51 | 0.083 | 1 | Β-hydroxybutyric | 2.46 | 0.136 | 1 |
| Methionine Sulfo | 1.26 | 0.069 | 2 | SM 22:2 OH | 2.00 | 0.110 | 2 |
| LysoPC 17:0 | 1.11 | 0.061 | 3 | Uric acid | 1.30 | 0.071 | 3 |
| Threonine | 1.09 | 0.059 | 4 | Glycine | 0.79 | 0.043 | 4 |
| Isoleucine | 0.94 | 0.052 | 5 | Glutamine | 0.71 | 0.039 | 5 |
| C18:2 | 0.89 | 0.048 | 6 | LysoPC 18:0 | 0.64 | 0.035 | 6 |
| LysoPC 18:1 | 0.84 | 0.046 | 7 | Betaine | 0.61 | 0.033 | 7 |
| Dimethylarginine | 0.67 | 0.036 | 8 | Methylhistidine | 0.51 | 0.028 | 8 |
| Leucine | 0.63 | 0.034 | 9 | SM 24:1 OH | 0.48 | 0.026 | 9 |
| C18:1 | 0.59 | 0.032 | 10 | Fumaric acid | 0.47 | 0.026 | 10 |
| LysoPC18:0 | 0.59 | 0.032 | 11 | Serine | 0.47 | 0.024 | 11 |
| Phenylalanine | 0.43 | 0.24 | 12 | Glutamate | 0.44 | 0.024 | 12 |
| Glutamate | 0.42 | 0.023 | 13 | Alanine | 0.44 | 0.023 | 13 |
| C18 | 0.41 | 0.022 | 14 | Age | 0.42 | 0.023 | 14 |
| Glutamine | 0.39 | 0.021 | 15 | Methionine | 0.41 | 0.022 | 15 |
| Age | 0.37 | 0.020 | 16 | C16:2 | 0.41 | 0.019 | 16 |
| Histidine | 0.36 | 0.020 | 17 | Marshall score | 0.35 | 0.018 | 17 |
| Citrulline | 0.35 | 0.019 | 18 | C16:1 | 0.33 | 0.018 | 18 |
| PC32:0 aa | 0.32 | 0.017 | 19 | C2 | 0.32 | 0.017 | 19 |
| C18:1 | 0.31 | 0.017 | 20 | C3OH | 0.32 | 0.015 | 20 |
| C3:1 | 0.29 | 0.016 | 21 | C14:1 | 0.28 | 0.015 | 21 |
| Marshall Score | 0.28 | 0.015 | 22 | C4:1 | 0.27 | 0.141 | 22 |
| PC38:6 aa | 0.27 | 0.014 | 23 | C4 | 0.27 | 0.013 | 23 |
| C16 | 0.26 | 0.014 | 24 | C3:1 | 0.25 | 0.013 | 24 |
| Asparagine | 0.25 | 0.014 | 25 | PC40:2 aa | 0.24 | 0.012 | 25 |
| SM 20:2 | 0.23 | 0.013 | 26 | C6:1 | 0.23 | 0.012 | 26 |
| PC36:6 aa | 0.23 | 0.012 | 27 | ɑ-aminoadipic acid | 0.23 | 0.012 | 27 |
| Citric acid | 0.21 | 0.011 | 28 | C4OH | 0.22 | 0.009 | 28 |
| C16:1 | 0.20 | 0.011 | 29 | Homocysteine | 0.17 | 0.008 | 29 |
| C16:1 OH | 0.20 | 0.011 | 30 | Pyruvic acid | 0.16 | 0.007 | 30 |
| Lactate | 0.20 | 0.011 | 31 | Taurine | 0.14 | 0.007 | 31 |
| Spermine | 0.20 | 0.011 | 32 | LysoPC 18:2 | 0.13 | 0.007 | 32 |
| Tyrosine | 0.18 | 0.010 | 33 | LysoPC 17:0 | 0.13 | 0.006 | 33 |
| Proline | 0.18 | 0.010 | 34 | LysoPC 26:0 | 0.12 | 0.006 | 34 |
| C4 OH | 0.15 | 0.008 | 235 | Trimethylamine | 0.12 | 0.006 | 35 |
| PC36:0 ae | 0.15 | 0.008 | 36 | hydroxyproline | 0.11 | 0.005 | 36 |
| C16:2 | 0.14 | 0.008 | 37 | Ornithine | 0.11 | 0.004 | 37 |
| C2 | 0.13 | 0.007 | 38 | C18:2 | 0.10 | 0.004 | 38 |
| LysoPC 16:0 | 0.13 | 0.007 | 39 | Indole acetic acid | 0.07 | 0.004 | 39 |
| C14:1 OH | 0.12 | 0.006 | 40 | Histidine | 0.07 | 0.004 | 40 |
| C14:2 | 0.11 | 0.006 | 41 | Hypoxemia | 0.06 | 0.004 | 41 |
| Hypoxemia | 0.10 | 0.005 | 42 | C14:2 OH | 0.06 | 0.003 | 42 |
| Valine | 0.07 | 0.004 | 43 | C5 | 0.05 | 0.003 | 43 |
| Methionine | 0.05 | 0.003 | 44 | ɑ-ketoglutaric acid | 0.05 | 0.002 | 44 |
| C5 MDC | 0.04 | 0.002 | 45 | C10:1 | 0.04 | 0.002 | 45 |
| LysoPC 18:2 | 0.001 | 0.001 | 46 | LysoPC 18:1 | 0.03 | 0.002 | 46 |
|  |  |  |  | PC36:0aa | 0.01 | 0.002 | 47 |
|  |  |  |  |  |  |  |  |
| **Day 1 Samples (GOSE-12 month) LC/DIMS-MS** | | | | **Day 4 Samples (GOSE-12 month) LC/DIMS-MS** | | | |
| **Predictor** | **Contribution** | **Portion** | **Rank** | **Predictor** | **Contribution** | **Portion** | **Rank** |
| LysoPC 20:4 | 1.65 | 0.079 | 1 | C3:1 | 1.70 | 0.097 | 1 |
| Trans-hydroxyproline | 1.36 | 0.066 | 2 | C18 | 1.00 | 0.058 | 2 |
| Ornithine | 1.06 | 0.051 | 3 | Β-hydroxybutyric | 0.92 | 0.053 | 3 |
| Spermine | 1.03 | 0.049 | 4 | C7DC | 0.71 | 0.041 | 4 |
| LysoPC 18:2 | 0.90 | 0.043 | 5 | Creatinine | 0.64 | 0.037 | 5 |
| Age | 0.87 | 0.042 | 6 | PC40:2 aa | 0.63 | 0.036 | 6 |
| C18:2 | 0.77 | 0.040 | 7 | Tryptophan | 0.61 | 0.035 | 7 |
| LysoPC 14:0 | 0.71 | 0.37 | 8 | C6 | 0.52 | 0.030 | 8 |
| C3 | 0.70 | 0.034 | 9 | Serotonin | 0.52 | 0.029 | 9 |
| Maximum GCS | 0.68 | 0.033 | 10 | ɑ-Ketoglutaric | 0.50 | 0.027 | 10 |
| Methionine Sulfo | 0.62 | 0.030 | 11 | Aspartate | 0.47 | 0.026 | 11 |
| Serine | 0.59 | 0.028 | 12 | LysoPC 17:0 | 0.46 | 0.026 | 12 |
| Lactate | 0.54 | 0.026 | 13 | LysoPC 28:1 | 0.46 | 0.025 | 13 |
| LysoPC 16:0 | 0.52 | 0.025 | 14 | Age | 0.44 | 0.024 | 14 |
| SM 16:1 | 0.48 | 0.023 | 15 | C14:2 | 0.41 | 0.023 | 15 |
| LysoPC 20:3 | 0.48 | 0.023 | 16 | SM 16:0 | 0.41 | 0.022 | 16 |
| C7 DC | 0.43 | 0.021 | 17 | Indole acetic acid | 0.38 | 0.021 | 17 |
| C6 | 0.43 | 0.020 | 18 | Spermidine | 0.38 | 0.021 | 18 |
| LysoPC 18:0 | 0.41 | 0.020 | 19 | Loss of Consciousness | 0.37 | 0.019 | 19 |
| Acetyl-ornithine | 0.36 | 0.017 | 20 | PC36:0 aa | 0.36 | 0.019 | 20 |
| SM 18:1 | 35 | 0.017 | 21 | Alanine | 0.33 | 0.017 | 21 |
| C14:2 | 0.34 | 0.016 | 22 | C16OH | 0.33 |  | 22 |
| Dimethylarginine | 0.34 | 0.016 | 23 | C16:2 | 0.30 | 0.016 | 23 |
| Trimethylamine | 0.33 | 0.016 | 24 | Trimethylamine | 0.28 | 0.016 | 24 |
| Methylhistidine | 0.33 | 0.015 | 25 | Valine | 0.28 | 0.015 | 25 |
| C16 | 0.32 | 0.015 | 26 | C6:1 | 0.27 | 0.015 | 26 |
| C14 | 0.32 | 0.015 | 27 | Betaine | 0.26 | 0.015 | 27 |
| Homocysteine | 0.31 | 0.014 | 28 | C4:1 | 0.26 | 0.013 | 28 |
| LysoPC 26:1 | 0.30 | 0.013 | 29 | Gender | 0.23 | 0.013 | 29 |
| C4:1 | 0.27 | 0.011 | 30 | C14 | 0.23 | 0.012 | 30 |
| C9 | 0.24 | 0.011 | 31 | C18:1 OH | 0.23 | 0.012 | 31 |
| LysoPC 17:0 | 0.23 | 0.009 | 32 | Spermine | 0.21 | 0.011 | 32 |
| Methylmalonic | 0.20 | 0.009 | 33 | C3 | 0.21 | 0.011 | 33 |
| Betaine | 0.19 | 0.009 | 34 | PC40:1 aa | 0.20 | 0.010 | 34 |
| SM 16:1 OH | 0.19 | 0.009 | 35 | Methylhistidine | 0.19 | 0.010 | 35 |
| Glutamate | 0.13 | 0.006 | 36 | LysoPC 20:3 | 0.17 | 0.009 | 36 |
| Serotonin | 0.11 | 0.005 | 37 | Homocysteine | 0.17 | 0.009 | 37 |
| C18:1 | 0.10 | 0.005 | 38 | PC38:0 aa | 0.17 | 0.008 | 38 |
| LysoPC 28:1 | 0.08 | 0.004 | 39 | PC40:6ae | 0.16 | 0.006 | 39 |
| C0 | 0.08 | 0.003 | 40 | C14:1 OH | 0.13 | 0.006 | 40 |
| PC36:0 ae | 0.07 | 0.002 | 41 | C16:1 | 0.11 | 0.005 | 41 |
| C5 | 0.05 | 0.001 | 42 | C16 | 0.11 | 0.005 | 42 |
| Hypoxemia | 0.03 | 0.001 | 43 | C2 | 0.11 | 0.003 | 43 |
| C4 | 0.02 | 0.001 | 44 | C3OH | 0.11 | 0.003 | 44 |
| C14:1 | 0.02 | 0.001 | 45 | Maximum GCS | 0.10 | 0.003 | 45 |

B

**Table S8A& B.** Predictor screening analysis shows the importance of clinical variables in the prediction models for the prognosis of GOSE outcome at 3 months and 12 months using DI/LC-MS/MS data. The Tables present the ranking of metabolites and clinical variables in each prediction model.

| **Sample Name** | **GOSe 3** | **GOSe 12** | **Gender** | **Age** | **Type of Injury** | **MAXGCS** | **Located Injuries in Head** | **Fractures** | **Abdominal Injuries** | **Thoracic Injuries** | **Spinal Fractures** | **Long bone injury** |
| --- | --- | --- | --- | --- | --- | --- | --- | --- | --- | --- | --- | --- |
| **TBI 84** | **5** | **5** | Male | 62 | Crashed to Cyclist | 7 | Right side of the head | Yes | Yes | No | No | No |
| **TBI 4** | **5** | **8** | Female | 24 | Falling | 4 | right side SDH | No | No | No | No | No |
| **TBI 93** | **5** | **6** | Male | 42 | Falling backward | 3 | Back of head | Yes | No | No | No | No |
| **TBI 58** | **6** | **7** | Male | 19 | Bumped Head accident | 8 | Along the tentorium (left and right) | No | No | No | No | No |
| **TBI 142** | **7** | **6** | Male | 20 | MVA | 3 | right front temporoparietal acute subdural | Yes | No | No | No | No |
| **TBI 144** | **5** | **5** | Female | 38 | Falling | 8 |  | No | No | No | No | No |
| **TBI 29** | **6** | **7** | Male | 76 | Falling | 8 | Right fronto-parietal | No | No | No | No | No |
| **TBI 129** | **5** | **6** | Male | 61 | Motorcycle accident | 3 | left frontotemporal (hematoma) | Yes | No | No | No | Yes |
| **TBI 146** | **5** | **N/A** | Female | 23 | Struck by Car | 3 |  | Yes | No | No | Yes | Yes |
| **TBI 110** | **4** | **4** | Male | 79 | Falling Bathroom | 8 | Left orbit | No | No | No | No | No |
| **TBI 115** | **3** | **7** | Male | 22 | falling 40 feet | 8 | Diffuse injury; most severe to the right frontal/temporal lobes | Yes | No | No | No | No |
| **TBI 118** | **3** | **3** | Male | 22 | MVA Polytrauma | 3 | Diffuse HI | Yes | No | Yes | Yes | Yes |
| **TBI 108** | **1** | **N/A** | Male | 79 | MVA | 8 | L orbit, L cheek, occipital | Yes | No | Yes | Yes | Yes |
| **TBI 121** | **1** | **N/A** | Male | 84 | MVA | 8 | Mostly to left side of head - global head injury | Yes | No | No | No | No |
| **TBI 114** | **1** | **N/A** | Female | 78 | Falling | 8 | R side | Yes | No | No | No | No |
| **TBI 72** | **1** | **N/A** | Male | 23 | Falling 3 story | 5 | Extensive skull fractures | Yes | No | No | No | No |
| **TBI 8** | **1** | **N/A** | Male | 62 | Falling | 8 | frontal/cerebellar | Yes | No | No | No | No |
| **TBI 82** | **3** | **3** | Male | 68 | falling 30 stairs | 7 | Back of head | Yes | No | No | Yes | No |
| **TBI 2** | **4** | **5** | Male | 27 |  | 7 | entire - multi area | Yes | No | Yes | No | No |
| **TBI 12** | **1** | **N/A** | Male | 66 | Falling | 8 | Right fronto-temporal | Yes | No | No | No | No |
| **TBI 96** | **3** | **3** | Male | 46 | penetrate head | 3 | left occipital | No | No | No | No | No |
| **TBI 91** | **1** | **N/A** | Male | 49 | MVA | 4 | occipital | Yes | No | Yes | No | No |
| **TBI 63** | **1** | **N/A** | Male | 61 | MVC | 6 | DAI - multi-compartmental intracranial hemorrhage | Yes | Yes | Yes | No | No |
| **TBI 138** | **3** | **3** | Female | 48 | MVA | 3 |  | Yes | Yes | Yes | Yes | Yes |
| **TBI 56** | **1** | **N/A** | Male | 61 | Falling | 8 | Occiput | No | No | No | No | No |
| **TBI 101** | **1** | **N/A** | Female | 70 | Bumped Head MVA | 3 |  | Yes | Yes | No | No | Yes |
| **TBI 99** | **1** | **N/A** | Male | 83 | Falling | 8 | No physical trauma seen, but nose was bleeding | No | No | No | No | No |
| **TBI 24** | **3** | **3** | Male | 47 | Motor Accident | 3 | bi hemispheric cortical sulci, basal cisterns, left frontal | Yes | No | No | Yes | No |
| **TBI 21** | **1** | **N/A** | Male | 51 | MVA | 3 | Right fronto-parietal | Yes | Yes | Yes | No | No |
| **TBI 17** | **1** | **N/A** | Male | 74 | Falling | 3 | Multifocal: extra axial SAH, subdural hemorrhage | Yes | No | No | No | No |
| **TBI 31** | **1** | **N/A** | Male | 71 | Falling | 7 | Brain Stem, Rt SDH, Lt SAH, Rt intraparenchymal/cerebellar | Yes | No | Yes | No | No |
| **TBI 102** | **1** | **N/A** | Male | 31 | Falling 4 story | 3 | occipital head lac. | Yes | Yes | Yes | Yes | Yes |
| **TBI 27** | **5** | **6** | Male | 20 | fall from standing height | 8 | Left frontal | Yes | No | Yes | Yes | No |
| **TBI 25** | **3** | **3** | Male | 35 | Falling | 3 | Focal=SDH/SAH, Diffuse=Hypoxic ischemic encephalopathy | Yes | No | Yes | Yes | No |
| **TBI 39** | **1** | **N/A** | Male | 50 | Struck by Car | 5 | Right Subdural hematoma, SAH, contusions | Yes | No | No | No | No |
| **TBI 36** | **1** | **N/A** | Female | 72 | Struck by Car | 3 | Diffuse SAH with IVH, hemorrhagic contusions/shear injury, SDH | Yes | No | Yes | No | Yes |
| **TBI 106** | **4** | **5** | Male | 49 | falling | 6 | Occiput | Yes | No | No | Yes | No |
| **TBI 125** | **1** | **N/A** | Female | 75 | fall from standing height | 3 | left occiput | Yes | No | No | No | No |
| **TBI 46** | **3** | **3** | Male | 24 | Falling | 7 | left frontal/temporal, shear injury | Yes | No | Yes | No | No |
| **TBI 44** | **3** | **3** | Female | 38 | MVA | 7 |  | Yes | Yes | Yes | Yes | Yes |
| **TBI 123** | **1** | **N/A** | Male | 80 | Struck by Car | 3 | right parietal - temporal (skull fracture) | Yes | No | Yes | Yes | Yes |
| **TBI 124** | **1** | **N/A** | Male | 50 | Falling in elevator | 6 | Back of head | Yes | No | No | No | No |
| **TBI 127** | **3** | **3** | Male | 71 | Slipped | 3 | Back of head | No | No | No | No | No |
| **TBI 137** | **1** | **N/A** | Male | 23 | MVA | 4 | Open head injury to forehead | Yes | No | Yes | Yes | No |
| **TBI 74** | **N/A** | **5** | Male | 22 | MVA | 6 | right left frontal lobe, left parietal/occipital lobe, corpus callosum | Yes | No | Yes | No | No |
| **TBI 76** | **N/A** | **3** | Female | 68 | MVA with SAH, AI | 7 | Right temporal bone, and left scalp | Yes | Yes | No | No | No |
| **TBI 51** | **N/A** | **3** | Male | 49 | fell | 3 | occipital | No | No | No | No | No |
| **TBI 119** | **N/A** | **7** | Male | 64 | Falling Alcohol | 8 | Frontal lobe and back of head | No | No | No | No | No |
| **TBI 112** | **N/A** | **6** | Male | 52 | Collided to tree | 3 | Back of head | Yes | No | No | No | No |
| **TBI 107** | **N/A** | **N/A** | Male | 55 | Falling Bumped Head | 9 | global head injury | Yes | No | No | No | No |
| **TBI 49** | **N/A** | **N/A** | Female | 31 | MVA | 7 | Left frontal and right parietal region | Yes | Yes | No | No | No |
| **TBI 83** | **N/A** | **N/A** | Male | 60 | MVA | 8 |  | Yes | Yes | Yes | No | No |
| **TBI 86** | **N/A** | **N/A** | Male | 45 | MVA | 3 | Forehead, left and right sides | Yes | No | Yes | Yes | No |
| **TBI 88** | **N/A** | **3** | Female | 42 | Falling Polytrauma | 3 | back of the head | Yes | Yes | Yes | No | Yes |
| **TBI 14** | **N/A** | **N/A** | Male | 18 | MVA | 3 | bifrontal, corpus callosum, left medial thalamus | Yes | Yes | Yes | Yes | Yes |
| **TBI 60** | **N/A** | **5** | Male | 22 | penetrate head | 3 | frontal & temporal lobes. Type of TBI: crush | Yes | No | No | No | No |
| **TBI 52** | **N/A** | **N/A** | Male | 65 | head bump | 7 | Right side of head | No | No | No | No | No |
| **TBI 140** | **N/A** | **N/A** | Male | 67 | MVA | 8 |  | Yes | No | Yes | Yes | Yes |
| **TBI 122** | **N/A** | **N/A** | Male | 29 | Falling | 8 | Right face/temporal area; Right eye is swollen shut | Yes | No | No | No | No |

**TableS9**, Patients characteristics based on the type of injury to the brain and other associated injuries, thoracic, and abdominal , long bone injuries

|  | sTBI (n=59) | |
| --- | --- | --- |
|  | No | Yes |
| Fractures | 14 | 45 |
| Abdominal injuries | 47 | 12 |
| Thoracic injuries | 37 | 22 |
| Spinal Fractures | 43 | 16 |
| Long Bone Fractures | 46 | 13 |

Table S10A

|  | sTBI (n=59) |
| --- | --- |
| Patients had no fractures, no abdominal and thoracic injuries | 9 |
| Patients had only facial and skull fractures without abdominal and thoracic injuries | 32 |

Table S10B

Tables S10 A and B, Summarized the polytrauma condition among sTBI

CanTBI collaborators:

The following investigators participated in this study: **Steering Committee** — J.S. Hutchison, B. Winston, J. Lacroix, W. Panenka, A. Colantonio, N. Silverberg, D. Clarke, **Biobanking task force** — H. Frndova, S. Vercauteren, J. Wong, L. Fernandes, B. Winston, J. Lacroix, V. Tram, A. Baker, M. Esser, D. Clarke, J. Hutchison, **CRF and Procedure manual task force** — J. Hutchison, J. Lacroix, B. Winston, L. Fernandes, E. Wilson, **Healthcare utilization task force** — A. Colantonio, B. Swaine, I. Gagnon, K. Yeates, L. Moore, J. Kutsogiannis, J. Hutchison, E. Wilson, C. Stapf, D. Clarke, F. Bernanrd, T. Tarling, **Outcomes task force** — A. Aquino, A. Baker, D. Clarke, M. Esser, L. Fenerty, L. Fernandes, H. Frndova, E. Gilfoyle, R. Green, A. Guerguerian, J. Hutchison, J. Lacroix, W. Panenka, N. Silverberg, V. Tram, S. Vercauteren, A. Wheeler, E. Wilson, B. Winston, J. Wong,

| Name | Location | Email |
| --- | --- | --- |
| Ari Joffe | University of Alberta, Stollery Children's Hospital, Alberta, Canada, | [Ari.joffe@albertahealthservices.ca](mailto:Ari.joffe@albertahealthservices.ca) |
| Karen Barlow | University of Queensland, Australia | [k.barlow@uq.edu.au](mailto:k.barlow@uq.edu.au) |
| Keith Yeates | Alberta Children’s Hospital, University of Calgary, Alberta, Canada | [kyeates@ucalgary.ca](mailto:kyeates@ucalgary.ca) |
| Michael Esser | Alberta Children’s Hospital, University of Calgary, Alberta, Canada | [michael.esser@albertahealthservices.ca](mailto:michael.esser@albertahealthservices.ca) |
| Brent Winston | University of Calgary, Foothill Medical Centre, Calgary, AB, Canada | [bwinston@ucalgary.ca](mailto:bwinston@ucalgary.ca) |
| Cheryl Wellington | University of British Colombia, BC, Canada | [cwell@mail.ubc.ca](mailto:cwell@mail.ubc.ca) |
| Ivan Torres | University of British Colombia, BC Children’s Hospital, BC, Canada | [ivan.torres@ubc.ca](mailto:ivan.torres@ubc.ca) |
| Keith Walley | University of British Colombia, St Paul’s Hospital, BC, Canada | [keith.walley@hli.ubc.ca](mailto:keith.walley@hli.ubc.ca) |
| Noah Silverberg | University of British Columbia, BC, Canada | [Noah.silverberg@ubc.ca](mailto:Noah.silverberg@ubc.ca) |
| Prescilla Carrion | University of British Colombia, BC Children’s Hospital, BC,Canada | [prescilla.carrion@ubc.ca](mailto:prescilla.carrion@ubc.ca) |
| Quynh Doan | University of British Columbia, BC Children’s Hospital, BC, Canada | [QDoan@cw.bc.ca](mailto:QDoan@cw.bc.ca) |
| Sophie Stukas | University of British Colombia, BC, Canada | [sophie.stukaTs@ubc.ca](mailto:sophie.stukaTs@ubc.ca) |
| Susan Vercauteren | University of British Colombia BC Children’s Hospital, BC, Canada | [SVercauteren2@cw.bc.ca>;](mailto:SVercauteren2@cw.bc.ca) |
| Will Panenka | University of British Colombia, BC Children’s Hospital, BC, Canada | [wpanenka@mail.ubc.ca](mailto:wpanenka@mail.ubc.ca) |
| Angela Aquino | University of British Columbia, BC, Canada | [angela.aquino@ubc.ca](mailto:angela.aquino@ubc.ca) |
| Audas Lorelei | Queen Elizabeth II Health Sciences Centre, NS, Canada | [Lorelei.audas@nshealth.ca](mailto:Lorelei.audas@nshealth.ca) |
| David Clarke | Queen Elizabeth II Health Sciences Centre, NS, Canada | [d.clarke@dal.ca](mailto:d.clarke@dal.ca) |
| Kelly Martin | Queen Elizabeth II Health Sciences Centre, NS, Canada | [Kelly.Martin@cdha.nshealth.ca](mailto:Kelly.Martin@cdha.nshealth.ca) |
| Adrienne Davis | Hospital for Sick Children | [adrienne.davis@sickkids.ca](mailto:adrienne.davis@sickkids.ca) |
| Angela Colantonio | Toronto Rehabilitation Institute, ON, Canada | [angela.colantonio@utoronto.ca](mailto:angela.colantonio@utoronto.ca) |
| Anne Wheeler | Hospital for Sick Children, ON, Canada | [anne.wheeler@sickkids.ca](mailto:anne.wheeler@sickkids.ca) |
| Anne-Marie Guerguerian | Hospital for Sick Children, ON, Canada | [anne-marie.guerguerian@sickkids.ca](mailto:anne-marie.guerguerian@sickkids.ca) |
| Andrew Baker | St Michael’s Hospital, ON, Canada | [BakerA@smh.ca](mailto:BakerA@smh.ca) |
| Elaine Gilfoyle | Hospital for Sick Children, ON, Canada | [elaine.gilfoyle@sickkids.ca](mailto:elaine.gilfoyle@sickkids.ca) |
| Jamie Hutchison | Hospital for Sick Children, ON, Canada | [Jamie.hutchison@sickkids.ca](mailto:Jamie.hutchison@sickkids.ca) |
| Lili-Naz Hazrati | Hospital for Sick Children, ON, Canada | [lili-naz.hazrati@sickkids.ca](mailto:lili-naz.hazrati@sickkids.ca) |
| Robin Green | Toronto Rehabilitation Institute, ON, Canada | [Robin.Green@uhn.ca](mailto:Robin.Green@uhn.ca) |
| Shannon Scratch | Holland Bloorview, ON, Canada | [sscratch@hollandbloorview.ca](mailto:sscratch@hollandbloorview.ca) |
| Elisa Wilson | Hospital for Sick Children, ON, Canada | [Elisa.wilson@sickkida.ca](mailto:Elisa.wilson@sickkida.ca) |
| Arash Khosroawshahi | Hospital for Sick Children, ON, Canada | [Arash.khosroawshahi@sickkids.ca](mailto:Arash.khosroawshahi@sickkids.ca) |
| Catherine Farrell | CHU Ste Justine, QC, Canada | [catherine.farrell.hsj@ssss.gouv.qc.ca](mailto:catherine.farrell.hsj@ssss.gouv.qc.ca) |
| Jacques Lacroix | CHU Ste Justine, QC, Canada | [jacques.lacroix.hsj@ssss.gouv.qc.ca](mailto:jacques.lacroix.hsj@ssss.gouv.qc.ca) |
